# Cancer risks in recipients of renal transplants: a meta-analysis of cohort studies

**DOI:** 10.18632/oncotarget.23841

**Published:** 2017-12-16

**Authors:** Yu Wang, Gong-Bin Lan, Feng-Hua Peng, Xu-Biao Xie

**Affiliations:** ^1^ Department of Urological Transplantation, The Second Xiangya Hospital of Central South University, Changsha, Hunan, China

**Keywords:** renal transplantation, cancer risk, meta-analysis

## Abstract

Renal transplantation is associated with an increased risk of cancers at multiple sites; however, the relationships between increased cancer risk and participant characteristics remain unclear. We searched PubMed, Embase, and the Cochrane Library to identify prospective observational studies performed up to July 2017. Totally 11 prospective studies reported data on 79,988 renal transplant recipients were included. Renal transplant recipients were found to display a higher risk of all cancers (standard incidence ratio [SIR]: 2.89; 95% CI: 2.13–3.91; *P* < 0.001), gastric cancer (SIR: 1.93; 95% CI: 1.60–2.34; *P* < 0.001), colon cancer (SIR: 1.85; 95% CI: 1.53–2.23; *P* < 0.001), pancreatic cancer (SIR: 1.53; 95% CI: 1.23–1.91; *P* < 0.001), hepatocellular carcinoma (SIR: 2.45; 95% CI: 1.63–3.66; *P* < 0.001), lung cancer (SIR: 1.68; 95% CI: 1.29–2.19; *P* < 0.001), thyroid cancer (SIR: 5.04; 95% CI: 3.79–6.71; *P* < 0.001), urinary bladder cancer (SIR: 3.52; 95% CI: 1.48–8.37; *P* = 0.004), renal cell cancer (SIR: 10.77; 95% CI: 6.40–18.12; *P* < 0.001), non-melanoma skin cancer (SIR: 12.14; 95% CI: 6.37–23.13; *P* < 0.001), melanoma (SIR: 2.48; 95% CI: 1.08–5.67; *P* = 0.032), Hodgkin's lymphoma (SIR: 4.90; 95% CI: 3.09–7.78; *P* < 0.001), non-Hodgkin lymphoma (SIR: 10.66; 95% CI: 8.54–13.31; *P* < 0.001), lip cancer (SIR: 29.45; 95% CI: 17.85–48.59; *P* < 0.001), breast cancer (SIR: 1.11; 95% CI: 1.00–1.24; *P* = 0.046), and ovarian cancer (SIR: 1.60; 95% CI: 1.23–2.07; *P* < 0.001). However, renal transplantation did not significantly influence the risks of uterine cancer (*P* = 0.171), and prostate cancers (*P* = 0.188). Our findings suggest that patients who receive renal transplantation have an increased risk of cancer at most sites, apart from uterine and prostate cancers patients.

## INTRODUCTION

Renal transplantation is considered the best treatment of choice to improve the survival and quality of life of patients with end-stage renal disease (ESRD) [1–2]. Previous studies have demonstrated that solid organ transplant recipients are known to have a higher risk of a variety of malignancies, in comparison with the general population [3–6]. While numerous studies have proposed reasons for the greater risk of cancer observed in transplant recipients, several scholars have specifically suggested that the increased cancer risk might due to long-term immunosuppressive agent exposure [7]. The associated risks of various cancers across a spectrum of renal transplant recipients remains poorly understood.

Several prospective studies have suggested that renal transplant recipients display an increased risk of cancer at multiple sites, although the associated findings are generally not consistent, especially across recipients that display a variety of characteristics [8–18]. It remains particularly important to determine the risk of cancer in renal transplant recipients, according to specific patient characteristics. Here, we performed a large-scale examination of available prospective cohort studies and determined relationships between patients who received renal transplantation and were diagnosed with cancer at different sites. We further compared these associations across participants with different baseline characteristics.

## RESULTS

### Literature search and study selection

**Figure 1:**
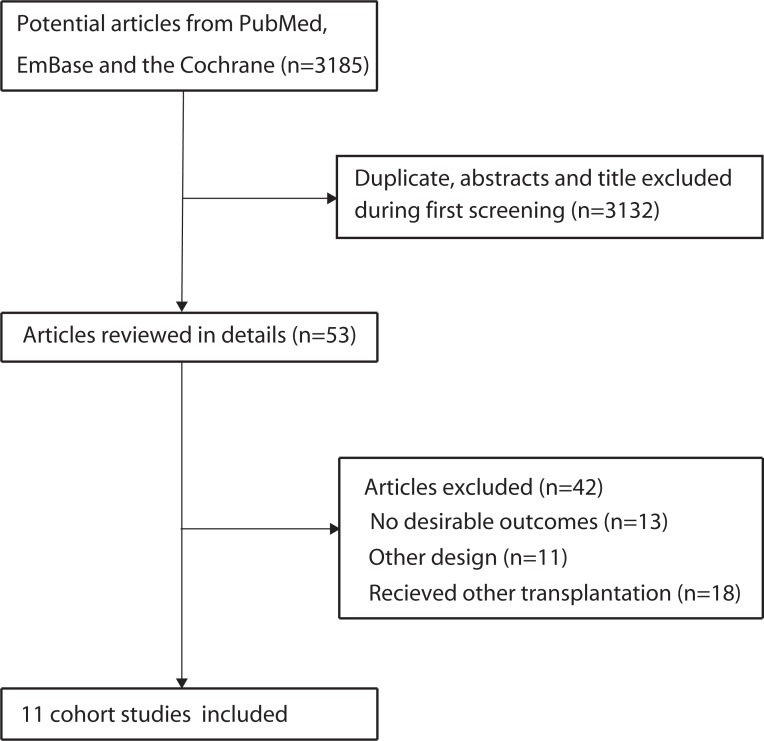
Flow diagram of the literature search and trials selection process

The study selection process is presented in Figure 1. There was a total of 3,185 relevant articles under the search words (PubMed: 418, Embase: 2,738, and Cochrane library: 29), of which 326 were excluded as duplicates. A total of 2,859 articles were identified through literature searches and screenings of the title and/or abstract, of which 2,806 were not related to the topic and excluded. After assessment of full-text articles (*n* = 53), 42 studies were excluded due to the absence of relevant data, other designs, or the presence of participants who received a non-renal solid organ transplant. Finally, 11 articles met all inclusion criteria and were included in our meta-analysis [8–18]. A manual search of the reference lists contained within these studies did not yield additional eligible studies, and general characteristics and qualities of included studies are displayed in Table 1.

**Table 1:** Baseline characteristic of studies included in the systematic review and meta-analysis

Study	Country	Type of transplant	Mean or median age (yr)	Sample size	Number of renal transplant cases	Number of all cancers	Reported outcomes	Follow-up duration (yr)	NOS score
Hoshida 1997 [8]	Japan	Renal	40.0	1744	1744	45	All cancers, GC, HCC, BC, UC, UBC, RCC, TC	7.4	8
Birkeland 2000 [9]	Denmark	Renal	38.9	1821	1821	209	All cancers, BC, UBC, RCC, Non-melanoma SC, LC, melanoma, TC, Hodgkin’s lymphoma, Non-Hodgkin lymphoma	7.5	8
Kyllonen 2000 [10]	Finland	Renal	41.5	2890	3440	230	All cancers, BC, UBC, RCC, TC, CC, lymphoma, leukaemia, LC, Non-melanoma SC	7.2	7
Vajdic 2006 [11]	Australia and New Zealand	Renal	41.0	10180	10180	1236	All cancers, BC, CC, HCC, LC, OC, PC, RCC, UBC, TC, UC, Hodgkin’s lymphoma, Non-Hodgkin lymphoma	8.5	8
Vegso 2007 [12]	Hungary	Renal	53.1	2535	2852	193	All cancers, BC, lung cancer, Non-melanoma SC, PC, UBC, GC, RCC, melanoma, HCC, Non-Hodgkin’s lymphoma, TC	9.8	7
Villeneuve 2007 [13]	Canada	Renal	NA	11 155	11 391	778	All cancers, LC, GC, CC, HCC, pancreatic cancer, lung cancer, BC, OC, UC, PC, RCC, UBC, TC, Hodgkin’s lymphoma, Non-Hodgkin lymphoma	7.3	7
Collett 2010 [14]	UK	Multiorgan	NA	25104	25104	4422	All cancers, LC, GC, CC, HCC, pancreatic cancer, lung cancer, Non-melanoma SC, BC, OC, PC, RCC, UBC, TC, UC	NA	8
Li 2012 [15]	China	Renal	44.1	4716	4716	320	All cancers, GC, CC, HCC, lung cancer, melanoma, Non-melanoma SC, OC, UBC, TC, UC	4.8	7
Cheung 2012 [16]	China	Renal	43.7	4674	4895	299	All cancers, GC, CC, HCC, pancreatic cancer, lung cancer, melanoma, BC, OC, PC, RCC, UBC, TC, UC	8.2	7
Krynitz 2013 [17]	Sweden	Multiorgan	NA	7952	7952	2774	All cancers, LC, GC, CC, HCC, pancreatic cancer, lung cancer, Non-melanoma SC, BC, OC, PC, RCC, UBC, TC, UC	9.7	8
Piselli 2013 [18]	Italy	Renal	NA	7217	7299	395	All cancers, LC, GC, HCC, pancreatic cancer, lung cancer, melanoma, BC, OC, PC, RCC, UBC, TC, UC, Hodgkin’s lymphoma,	5.5	8

^*^GC: gastric cancer; HCC: hepatocellular carcinoma; BC: breast cancer; UC: uterine cancer; UBC: urinary bladder cancer; RCC: renal cell cancer; TC: thyroid cancer; SC: skin cancer; CC: colon cancer; LC: lip cancer; OC: ovarian cancer; PC: prostate cancer.

### Study characteristics

Our study was performed on eleven prospective cohorts, which involved a total of 79,988 renal transplant recipients. The follow-up period for participants was 4.8–9.8 years, and 1, 744–25, 104 patients were included in each study. 8 studies were conducted in Western countries [9–14, 17, 18], and the remaining 3 were conducted in Eastern countries [8, 15, 16]. The search terms all cancer were reported in 11 studies, gastric cancer in 8 studies, colon cancer in 8 studies, pancreatic cancer in 6 studies, hepatocellular carcinoma in 9 studies, lung cancer in 7 studies, thyroid cancer in 11 studies, urinary bladder cancer in 11 studies, renal cell cancer in 11 studies, non-melanoma skin cancer in 7 studies, melanoma in 5 studies, Hodgkin's lymphoma in 5 studies, non-Hodgkin's lymphoma in 6 studies, lip cancer in 7 studies, breast cancer in 11 studies, ovarian cancer in 6 studies, uterine cancer in 8 studies, and prostate cancer in 8 studies. All included studies showed moderate and high qualities with acceptable and moderate risks of bias (Table 1). Six cohorts had a score of 8, while the remaining 5 studies had a score of 7.

### Cancer risk in renal transplant recipients

**Figure 2:**
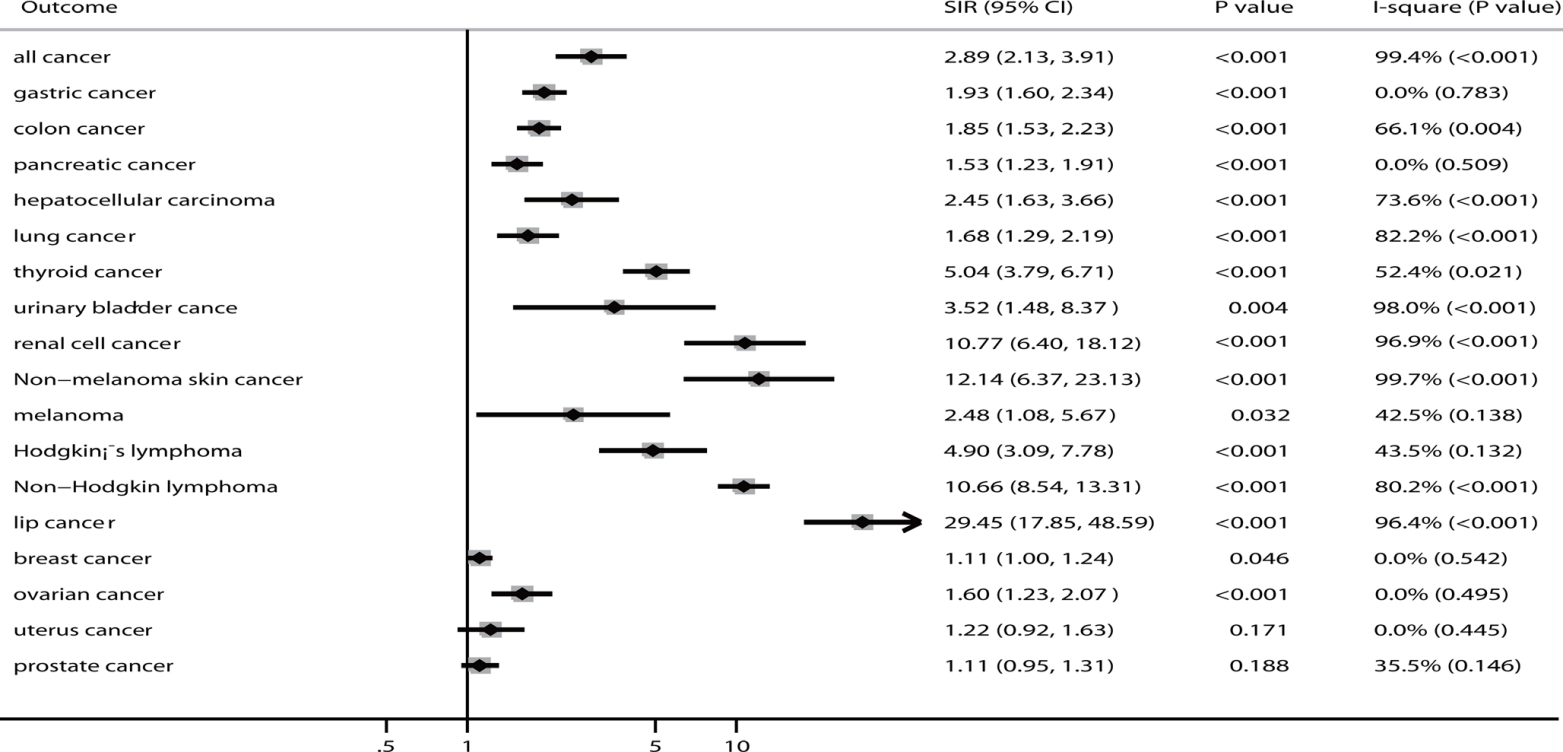
The summary results for cancer risk in recipients of renal transplants

The summary of results for cancer at different sites is presented in Figure 2 and Supplementary Figure 1. We found that renal transplant recipients were associated with a higher risk of all cancer (SIR: 2.89; 95% CI: 2.13–3.91; *P* < 0.001), gastric cancer (SIR: 1.93; 95% CI: 1.60–2.34; *P* < 0.001), colon cancer (SIR: 1.85; 95% CI: 1.53–2.23; *P* < 0.001), pancreatic cancer (SIR: 1.53; 95% CI: 1.23–1.91; *P* < 0.001), hepatocellular carcinoma (SIR: 2.45; 95% CI: 1.63–3.66; *P* < 0.001), lung cancer (SIR: 1.68; 95% CI: 1.29–2.19; *P* < 0.001), thyroid cancer (SIR: 5.04; 95% CI: 3.79–6.71; *P* < 0.001), urinary bladder cancer (SIR: 3.52; 95% CI: 1.48–8.37; *P* = 0.004), renal cell cancer (SIR: 10.77; 95% CI: 6.40–18.12; *P* < 0.001), non-melanoma skin cancer (SIR: 12.14; 95% CI: 6.37–23.13; *P* < 0.001), melanoma (SIR: 2.48; 95% CI: 1.08–5.67; *P* = 0.032), Hodgkin's lymphoma (SIR: 4.90; 95% CI: 3.09–7.78; *P* < 0.001), non-Hodgkin's lymphoma (SIR: 10.66; 95% CI: 8.54–13.31; *P* < 0.001), lip cancer (SIR: 29.45; 95% CI: 17.85–48.59; *P* < 0.001), breast cancer (SIR: 1.11; 95% CI: 1.00–1.24; *P* = 0.046), and ovarian cancer (SIR: 1.60; 95% CI: 1.23–2.07; *P* < 0.001). However, there was no significant effect of renal transplants on the risk of uterine cancer (SIR: 1.22; 95% CI: 0.92–1.63; *P* = 0.171) and prostate cancer (SIR: 1.11; 95% CI: 0.95–1.31; *P* = 0.188). We identified that substantial heterogeneity existed among the results, with the exceptions of gastric cancer, pancreatic cancer, breast cancer, ovarian cancer, and uterine cancer.

The results of our sensitivity analyses are listed in Supplementary Figure 2 and indicate that our conclusions were not affected for all cancer, gastric cancer, colon cancer, pancreatic cancer, hepatocellular carcinoma, lung cancer, thyroid cancer, urinary bladder cancer, renal cell cancer, non-melanoma skin cancer, Hodgkin's lymphoma, non-Hodgkin's lymphoma, lip cancer, ovarian cancer, uterine cancer, and prostate cancer, with respect to exclusion of any specific study. However, the variable findings observed for melanoma and breast cancer may be attributed to the smaller number of included cohorts or because renal transplantation effects, in these cases, were mild and require further verification across a large-scale study.

### Cancer risk in men and women respectively

**Figure 3:**
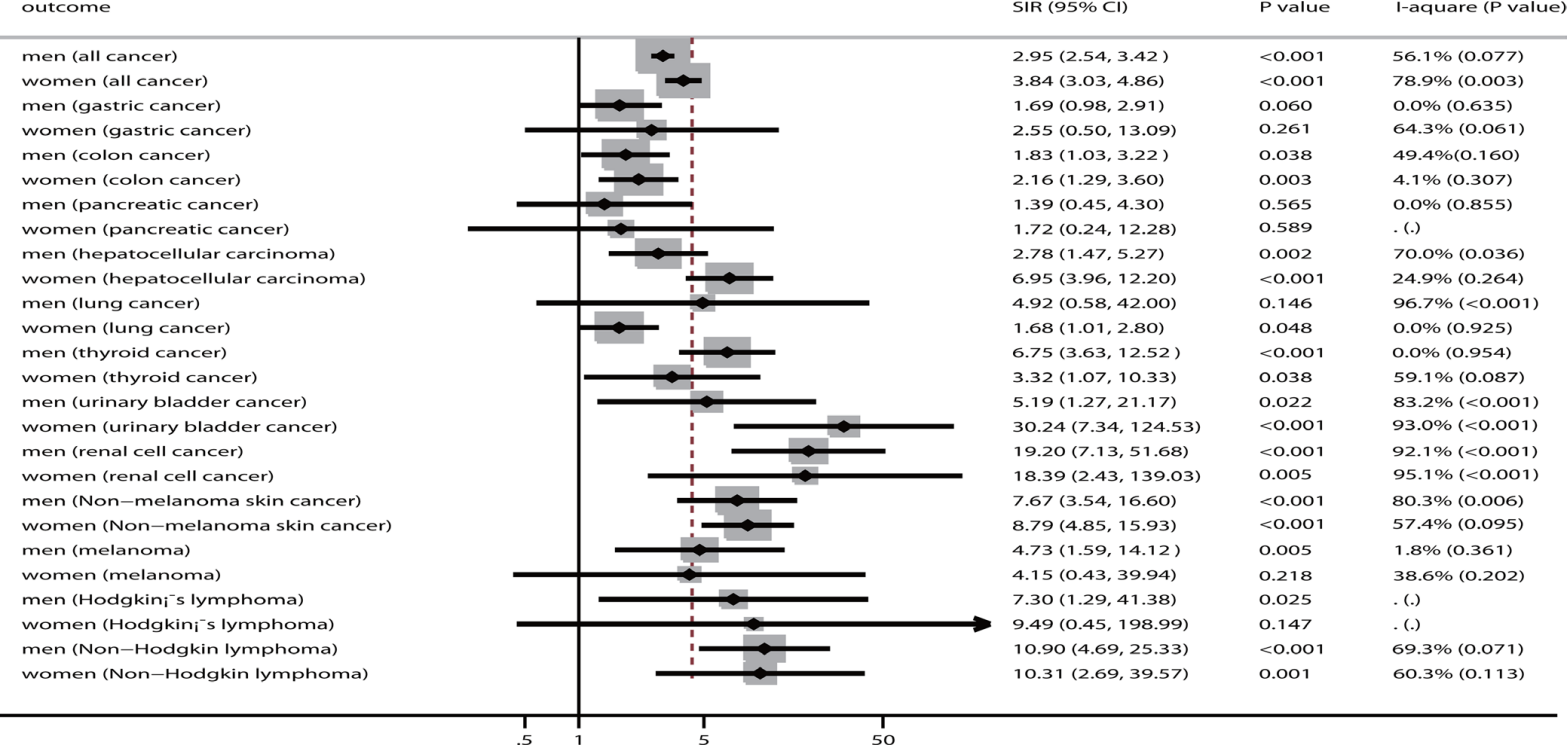
The summary results for cancer risk in men and women, respectively

The findings of cancer risks in men and women are presented in Figure 3 and Supplementary Figure 3. The summary results for men who received renal transplantation reveal that this group has a greater risk of all cancer (SIR: 2.95; 95% CI: 2.54–3.42; *P* < 0.001), colon cancer (SIR: 1.83; 95% CI: 1.03–3.22; *P* = 0.038), hepatocellular carcinoma (SIR: 2.78; 95% CI: 1.47–5.27; *P* = 0.002), thyroid cancer (SIR: 6.75; 95% CI: 3.63–12.52; *P* < 0.001), urinary bladder cancer (SIR: 5.19; 95% CI: 1.27–21.17; *P* = 0.022), renal cell cancer (SIR: 19.20; 95% CI: 7.13–51.68; *P* < 0.001), non-melanoma skin cancer (SIR: 7.67; 95% CI: 3.54–16.60; *P* < 0.001), melanoma (SIR: 4.73; 95% CI: 1.59–14.12; *P* = 0.005), Hodgkin's lymphoma (SIR: 7.30; 95% CI: 1.29–41.38; *P* = 0.025), and non-Hodgkin's lymphoma (SIR: 10.90; 95% CI: 4.69–25.33; *P* < 0.001) compared with the general population. Furthermore, women who received renal transplants displayed elevated risks of all cancer (SIR: 3.84; 95% CI: 3.03–4.86; *P* < 0.001), colon cancer (SIR: 2.16; 95% CI: 1.29–3.60; *P* = 0.003), hepatocellular carcinoma (SIR: 6.95; 95% CI: 3.96–12.20; *P* < 0.001), lung cancer (SIR: 1.68; 95% CI: 1.01–2.80; *P* = 0.048), thyroid cancer (SIR: 3.32; 95% CI: 1.07–10.33; *P* = 0.038), urinary bladder cancer (SIR: 30.24; 95% CI: 7.34–124.53; *P* < 0.001), renal cell cancer (SIR: 18.39; 95% CI: 2.43–139.03; *P* = 0.005), non-melanoma skin cancer (SIR: 8.79; 95% CI: 4.85–15.93; *P* < 0.001), and non-Hodgkin's lymphoma (SIR: 10.31; 95% CI: 2.69–39.57; *P* = 0.001). Additionally, men who underwent renal transplants displayed lower risks of hepatocellular carcinoma than women who received renal transplants (ratio of SIR: 0.40; 95% CI: 0.17–0.94; *P* = 0.035; Table 2).

**Table 2:** Subgroup analysis for cancer risks in recipients of renal transplants

Cancer	Factor	Groups	Number of studies	SIR and 95% CI	*P* value	Heterogeneity (%)	*P* value for heterogeneity	Ratio between subgroups	*P* value between subgroups
All cancer	Publication year	2010 or after	5	3.11 (1.79–5.43)	< 0.001	99.7	< 0.001	1.12 (0.62–2.01)	0.707
		Before 2010	6	2.78 (2.30–3.35)	< 0.001	93.5	< 0.001		
	Country	Eastern	3	3.19 (2.62–3.89)	< 0.001	80.9	0.005	1.14 (0.75–1.74)	0.547
		Western	8	2.80 (1.92–4.07)	< 0.001	99.6	< 0.001		
	Gender	Male	4	2.95 (2.54–3.42)	< 0.001	56.1	0.077	0.77 (0.58–1.02)	0.064
		Female	4	3.84 (3.03–4.86)	< 0.001	78.9	0.003		
	Follow-up duration (yrs)	8 or greater	4	3.10 (1.91–5.04)	< 0.001	99.4	< 0.001	1.09 (0.62–1.91)	0.759
		< 8	6	2.84 (2.15–3.75)	< 0.001	97.1	< 0.001		
Gastric cancer	Publication year	2010 or after	5	1.94 (1.57–2.40)	< 0.001	0.0	0.497	1.02 (0.63–1.66)	0.933
		Before 2010	3	1.90 (1.23–2.94)	0.004	0.0	0.742		
	Country	Eastern	3	2.19 (1.45–3.29)	< 0.001	0.0	0.371	1.17 (0.74–1.86)	0.503
		Western	5	1.87 (1.51–2.32)	< 0.001	0.0	0.817		
	Gender	Male	3	1.69 (0.98–2.91)	0.060	0.0	0.635	0.66 (0.12–3.70)	0.639
		Female	3	2.55 (0.50–13.09)	0.261	64.3	0.061		
	Follow-up duration (yrs)	8 or greater	3	2.22 (1.52–3.24)	< 0.001	0.0	0.507	1.31 (0.80–2.15)	0.276
		< 8	4	1.69 (1.24–2.32)	0.001	0.0	0.712		
Colon cancer	Publication year	2010 or after	4	1.92 (1.69–2.17)	< 0.001	9.9	0.343	1.16 (0.71–1.90)	0.564
		Before 2010	4	1.66 (1.03–2.68)	0.036	80.9	0.001		
	Country	Eastern	2	1.83 (1.35–2.48)	< 0.001	0.0	0.657	0.99 (0.68–1.46)	0.978
		Western	6	1.84 (1.45–2.33)	< 0.001	75.5	0.001		
	Gender	Male	2	1.83 (1.03–3.22)	0.038	49.4	0.160	0.85 (0.39–1.82)	0.672
		Female	2	2.16 (1.29–3.60)	0.003	4.1	0.307		
	Follow-up duration (yrs)	8 or greater	4	1.74 (1.27–2.40)	0.001	71.4	0.015	0.81 (0.40–1.61)	0.539
		< 8	3	2.16 (1.17–3.98)	0.014	79.8	0.007		
Pancreatic cancer	Publication year	2010 or after	5	1.57 (1.25–1.97)	< 0.001	0.0	0.454	1.43 (0.59–3.45)	0.429
		Before 2010	1	1.10 (0.47–2.58)	0.827	-	-		
	Country	Eastern	2	1.33 (0.50–3.53)	0.569	0.0	0.560	0.87 (0.31–2.41)	0.788
		Western	4	1.53 (1.14–2.07)	0.005	22.2	0.277		
	Gender	Male	2	1.39 (0.45–4.30)	0.565	0.0	0.855	0.81 (0.08–7.81)	0.854
		Female	1	1.72 (0.24–12.28)	0.589	-	-		
	Follow-up duration (yrs)	8 or greater	2	2.08 (1.32–3.28)	0.002	0.0	0.591	2.12 (1.00–4.51)	0.051
		< 8	3	0.98 (0.54–1.80)	0.959	0.0	0.931		
Hepatocellular carcinoma	Publication year	2010 or after	5	2.44 (1.43–4.16)	0.001	85.1	< 0.001	0.99 (0.48–2.05)	0.974
		Before 2010	4	2.47 (1.51–4.06)	< 0.001	0.0	0.586		
	Country	Eastern	3	3.11 (1.60–6.03)	0.001	80.2	0.006	1.45 (0.65–3.21)	0.365
		Western	6	2.15 (1.38–3.35)	0.001	50.2	0.074		
	Gender	Male	3	**2.78 (1.47–5.27)**	**0.002**	**70.0**	**0.036**	**0.40 (0.17–0.94)**	**0.035**
		Female	3	**6.95 (3.96–12.20)**	**< 0.001**	**24.9**	**0.264**		
	Follow-up duration (yrs)	8 or greater	4	2.73 (2.05–3.63)	< 0.001	0.0	0.931	1.67 (0.50–5.66)	0.406
		< 8	4	1.63 (0.50–5.33)	0.421	86.7	< 0.001		
Lung cancer	Publication year	2010 or after	5	1.71 (1.26–2.33)	0.001	81.3	< 0.001	1.35 (0.41–4.44)	0.625
		Before 2010	2	1.27 (0.40–4.02)	0.682	83.0	0.015		
	Country	Eastern	2	2.78 (0.99–7.78)	0.052	89.3	0.002	1.88 (0.65–5.44)	0.245
		Western	5	1.48 (1.14–1.91)	0.003	79.0	0.001		
	Gender	Male	2	4.92 (0.58–42.00)	0.146	96.7	< 0.001	2.93 (0.32–26.46)	0.339
		Female	2	1.68 (1.01–2.80)	0.048	0.0	0.925		
	Follow-up duration (yrs)	8 or greater	3	1.52 (1.08–2.14)	0.016	48.9	0.141	0.71 (0.34–1.46)	0.349
		< 8	3	2.15 (1.13–4.07)	0.019	90.4	< 0.001		
Thyroid cancer	Publication year	2010 or after	5	3.78 (2.30–6.19)	< 0.001	71.9	0.007	0.58 (0.34–1.02)	0.057
		Before 2010	6	6.48 (5.05–8.32)	< 0.001	0.0	0.697		
	Country	Eastern	3	4.37 (2.10–9.08)	< 0.001	54.8	0.109	0.82 (0.37–1.82)	0.625
		Western	8	5.33 (3.90–7.28)	< 0.001	52.5	0.040		
	Gender	Male	4	6.75 (3.63–12.52)	< 0.001	0.0	0.954	2.03 (0.56–7.40)	0.282
		Female	3	3.32 (1.07–10.33)	0.038	59.1	0.087		
	Follow-up duration (yrs)	8 or greater	4	5.59 (4.24–7.36)	< 0.001	0.0	0.403	1.24 (0.66–2.33)	0.499
		< 8	6	4.50 (2.56–7.92)	< 0.001	65.1	0.014		
Urinary bladder cancer	Publication year	2010 or after	5	4.51 (1.04–19.56)	0.044	99.1	< 0.001	1.60 (0.35–7.24)	0.542
		Before 2010	6	2.82 (1.98–4.03)	< 0.001	36.3	0.164		
	Country	Eastern	3	**14.74 (3.66–59.35)**	**< 0.001**	**93.7**	**< 0.001**	**6.70 (1.62–27.73)**	**0.009**
		Western	8	**2.20 (1.67–2.91)**	**< 0.001**	**67.2**	**0.003**		
	Gender	Male	4	5.19 (1.27–21.17)	0.022	83.2	< 0.001	0.17 (0.02–1.26)	0.083
		Female	3	30.24 (7.34–124.53)	< 0.001	93.0	< 0.001		
	Follow-up duration (yrs)	8 or greater	4	3.10 (1.61–5.98)	0.001	86.7	< 0.001	0.69 (0.12–4.05)	0.677
		< 8	6	4.52 (0.87–23.57)	0.074	98.4	< 0.001		
Renal cell cancer	Publication year	2010 or after	5	10.62 (4.56–24.73)	< 0.001	98.3	< 0.001	0.97 (0.33–2.85)	0.957
		Before 2010	6	10.94 (5.61–21.32)	< 0.001	93.1	< 0.001		
	Country	Eastern	3	**35.03(13.70–89.56)**	**< 0.001**	**95.2**	**< 0.001**	**4.98 (1.93–12.80)**	**0.001**
		Western	8	**7.04 (6.31–7.85)**	**< 0.001**	**10.7**	**0.347**		
	Gender	Male	4	19.20 (7.13–51.68)	< 0.001	92.1	< 0.001	1.04 (0.11–9.93)	0.970
		Female	3	18.39 (2.43–139.03)	0.005	95.1	< 0.001		
	Follow-up duration (yrs)	8 or greater	4	7.98 (5.79–10.98)	< 0.001	66.9	0.028	0.59 (0.22–1.63)	0.311
		< 8	6	13.47 (5.15–35.22)	< 0.001	97.9	< 0.001		
Non-melanoma skin cancer	Publication year	2010 or after	4	12.49 (5.27–29.63)	< 0.001	99.8	< 0.001	1.08 (0.27–4.29)	0.909
		Before 2010	3	11.53 (3.95–33.69)	< 0.001	97.0	< 0.001		
	Country	Eastern	2	4.50 (1.45–13.92)	0.009	78.2	0.032	0.26 (0.07–1.00)	0.051
		Western	5	17.31 (8.26–36.27)	< 0.001	99.8	< 0.001		
	Gender	Male	3	7.67 (3.54–16.60)	< 0.001	80.3	0.006	0.87 (0.33–2.31)	0.784
		Female	3	8.79 (4.85–15.93)	< 0.001	57.4	0.095		
	Follow-up duration (yrs)	8 or greater	3	10.25 (1.61–65.23)	0.014	98.7	< 0.001	0.86 (0.10–7.14)	0.887
		< 8	3	11.96 (4.25–33.65)	< 0.001	96.5	< 0.001		
Melanoma	Publication year	2010 or after	3	3.75 (1.20–11.73)	0.023	59.1	0.087	3.64 (0.65–20.25)	0.140
		Before 2010	2	1.09 (0.30–3.90)	0.894	0.0	0.439		
	Country	Eastern	2	**7.64 (2.46–23.69)**	**< 0.001**	**0.0**	**0.671**	**4.72 (1.32–16.83)**	**0.017**
		Western	3	**1.62 (0.91–2.90)**	**0.101**	**0.0**	**0.586**		
	Gender	Male	3	4.73 (1.59–14.12)	0.005	1.8	0.361	1.14 (0.09–14.10)	0.919
		Female	2	4.15 (0.43–39.94)	0.218	38.6	0.202		
	Follow-up duration (yrs)	8 or greater	2	2.51 (0.15–42.24)	0.524	76.9	0.037	1.30 (0.07–23.11)	0.858
		< 8	3	1.93 (1.09–3.41)	0.024	0.0	0.547		
Hodgkin’s lymphoma	Publication year	2010 or after	2	4.93 (1.66–14.67)	0.004	65.5	0.089	1.24 (0.37–4.11)	0.726
		Before 2010	3	3.98 (2.41–6.56)	< 0.001	0.0	0.650		
	Country	Eastern	0	-	-	-	-	-	-
		Western	5	4.90 (3.09–7.78)	< 0.001	43.5	0.132		
	Gender	Male	1	7.30 (1.29–41.38)	0.025	-	-	0.77 (0.02–25.60)	0.883
		Female	1	9.49 (0.45–198.99)	0.147	-	-		
	Follow-up duration (yrs)	8 or greater	1	3.74 (1.66–8.45)	0.002	-	-	1.01 (0.37–2.74)	0.979
		< 8	3	3.69 (2.08–6.53)	< 0.001	0.0	0.484		
Non-Hodgkin lymphoma	Publication year	2010 or after	2	**13.52 (10.89–16.78)**	**< 0.001**	**56.7**	**0.129**	**1.50 (1.13–2.00)**	**0.005**
		Before 2010	4	**9.01 (7.47–10.86)**	**< 0.001**	**36.9**	**0.191**		
	Country	Eastern	1	**15.79 (11.90–20.95)**	**< 0.001**	**-**	**-**	**1.62 (1.12–2.33)**	**0.010**
		Western	5	**9.77 (7.74–12.34)**	**< 0.001**	**79.5**	**0.001**		
	Gender	Male	2	10.90 (4.69–25.33)	< 0.001	69.3	0.071	1.06 (0.22–5.17)	0.945
		Female	2	10.31 (2.69–39.57)	0.001	60.3	0.113		
	Follow-up duration (yrs)	8 or greater	3	11.42 (6.92–18.84)	< 0.001	81.6	0.004	1.34 (0.77–2.32)	0.295
		< 8	2	8.52 (6.82–10.64)	< 0.001	6.6	0.301		
Lip cancer	Publication year	2010 or after	3	38.45 (21.03–70.32)	< 0.001	87.1	< 0.001	1.49 (0.58–3.82)	0.410
		Before 2010	4	25.86 (12.53–53.38)	< 0.001	97.7	< 0.001		
	Country	Eastern	0	-	-	-	-	-	-
		Western	7	29.45 (17.85–48.59)	< 0.001	96.4	< 0.001		
	Follow-up duration (yrs)	8 or greater	2	**46.83 (42.04–52.16)**	**< 0.001**	**0.0**	**0.883**	**2.56 (1.42–4.62)**	**0.002**
		< 8	4	**18.27 (10.23–32.63)**	**< 0.001**	**89.7**	**< 0.001**		
Breast cancer	Publication year	2010 or after	5	1.09 (0.91–1.32)	0.336	31.3	0.213	0.93 (0.73–1.20)	0.578
		Before 2010	6	1.17 (0.99–1.38)	0.072	0.0	0.762		
	Country	Eastern	3	1.40 (0.98–2.01)	0.068	0.0	0.607	1.28 (0.88–1.87)	0.191
		Western	8	1.09 (0.98–1.22)	0.128	0.0	0.513		
	Follow-up duration (yrs)	8 or greater	4	1.16 (0.96–1.39)	0.123	9.2	0.347	0.99 (0.76–1.29)	0.949
		< 8	6	1.17 (0.97–1.42)	0.092	0.0	0.543		
Ovarian cancer	Publication year	2010 or after	4	1.72 (1.22–2.42)	0.002	17.7	0.302	1.30 (0.67–2.55)	0.439
		Before 2010	2	1.32 (0.74–2.34)	0.348	0.0	0.651		
	Country	Eastern	1	3.29 (1.37–7.90)	0.008	-	-	2.21 (0.88–5.53)	0.091
		Western	5	1.49 (1.13–1.95)	0.004	0.0	0.825		
	Follow-up duration (yrs)	8 or greater	3	1.90 (1.15–3.13)	0.012	32.0	0.230	1.37 (0.58–3.22)	0.474
		< 8	2	1.39 (0.69–2.77)	0.357	0.0	0.702		
Uterine cancer	Publication year	2010 or after	5	1.08 (0.77–1.51)	0.658	0.0	0.967	0.59 (0.24–1.44)	0.246
		Before 2010	3	1.83 (0.80–4.17)	0.152	51.9	0.125		
	Country	Eastern	3	1.97 (0.89–4.38)	0.094	24.7	0.265	1.79 (0.76–4.22)	0.183
		Western	5	1.10 (0.80–1.51)	0.552	0.0	0.775		
	Follow-up duration (yrs)	8 or greater	3	1.25 (0.78–1.98)	0.352	0.0	0.525	0.84 (0.38–1.87)	0.678
		< 8	4	1.48 (0.78–2.84)	0.232	33.9	0.209		
Prostate cancer	Publication year	2010 or after	5	1.22 (1.00–1.49)	0.052	42.8	0.136	1.34 (0.98–1.83)	0.065
		Before 2010	3	0.91 (0.72–1.16)	0.459	0.0	0.787		
	Country	Eastern	2	1.18 (0.60–2.35)	0.628	16.9	0.273	1.06 (0.53–2.15)	0.865
		Western	6	1.11 (0.93–1.32)	0.251	48.0	0.087		
	Follow-up duration (yrs)	8 or greater	4	1.04 (0.89–1.22)	0.593	0.0	0.662	0.78 (0.46–1.32)	0.357

### Subgroup analyses

The summary of results from our subgroup analyses are shown in Table 2. First, we noted that the risk of all cancers persisted increased in pre-defined subsets, and we did not observe a significant SIR ratio between subgroups. Second, we found that renal transplants were not associated with a greater risk of gastric cancer in men and women, respectively. Third, renal transplant recipients have a greater risk of colon cancer than the general population across all subsets. Fourth, we observed an increased risk of pancreatic cancer in patients who received renal transplantation when the study published in 2010 or after, and in patients from study conducted in Western countries, and in patients with follow-up durations greater than 8.0 years. Fifth, we did not find a significant relationship between renal transplantation and risk of hepatocellular carcinoma if follow-up duration was less than 8.0 years, and women have greater risk of hepatocellular carcinomas than men, respectively, who received renal transplantation. Sixth, renal transplants were not associated with lung cancer risk in studies published before 2010, those conducted in Eastern countries, and the study that only included men. Seventh, the risk of thyroid cancer was observed to increased in all subsets.

Eighth, the risk of urinary bladder cancer was not statistically significant when follow-up duration was less than 8.0 years, and patients who received renal transplantation in Eastern countries had a greater risk of urinary bladder cancer than those in Western countries. Ninth, the risk of renal cell cancer increased in all pre-defined subsets, and SIR ratios showed a significant increase between renal transplants and risk of renal cell cancer in Eastern countries compared with Western counties. Tenth, the risk of non-melanoma skin cancer increased in all subsets, and no significant differences were observed between subgroups. Eleventh, renal transplant recipients displayed greater risks of melanoma in studies conducted during or after 2010, the study conducted in Eastern countries, the study that only included men, and when the duration of follow-up was less than 8.0 years. Furthermore, the summary SIR ratio (Eastern countries to Western countries) of renal transplant recipients suggested a greater risk for melanoma.

Twelfth, renal transplants were not observed to have a significant effect on Hodgkin's lymphoma, in studies that only included women. Thirteenth, renal transplant recipients displayed an increased non-Hodgkin lymphoma risk in all pre-defined subsets, and patients in studies published in 2010 or after and those conducted in Eastern countries were found to have greater risk of non-Hodgkin lymphoma than those in other subsets. Fourteenth, the risk of lip cancer increased for renal transplant patients, and patients with longer follow-up durations had an elevated risk compared to those with shorter follow-up durations. Fifteenth, renal transplants were not observed to have a significant effect on breast cancer in all pre-defined subsets. Sixteenth, renal transplants were not associated with risk of ovarian cancer in studies published before 2010 and those with shorter follow-up duration. Finally, there were no significant relationships between renal transplantation and the risk of uterine or prostate cancers.

### Publication biases

Our review of funnel plots could not rule out the potential for publication bias for cancer at different sites (Supplementary Figure 4). The Egger and Begg test results showed no evidence of publication bias for all cancer, gastric cancer, colon cancer, pancreatic cancer, lung cancer, thyroid cancer, urinary bladder cancer, renal cell cancer, non-melanoma skin cancer, melanoma, Hodgkin's lymphoma, non-Hodgkin's lymphoma, lip cancer, breast cancer, ovarian cancer, uterine cancer, and prostate cancer. Interestingly, while the Begg test showed no evidence of publication bias for hepatocellular carcinoma (*P* = 0.917), the Egger test suggested potential evidence of publication bias (*P* = 0.027) for this cancer. Our conclusions remained unchanged after adjustment for publication bias using the trim and fill method [19].

## DISCUSSION

Our current study was based on prospective cohort studies and explored all possible correlations between renal transplantation and the risk of cancer at different sites. This large quantitative study included 79,988 patients from 11 prospective cohort studies that covered a broad range of individuals. The findings from our current meta-analysis suggest that renal transplant recipients, in comparison with the general population, do not have different incidences of uterine cancer and prostate cancer. However, renal transplant recipients displayed a significantly elevated risk of all cancer, gastric cancer, colon cancer, pancreatic cancer, hepatocellular carcinoma, lung cancer, thyroid cancer, urinary bladder cancer, renal cell cancer, non-melanoma skin cancer, melanoma, Hodgkin's lymphoma, non-Hodgkin's lymphoma, lip cancer, breast cancer, and ovarian cancer. Furthermore, the findings of our stratified analyses indicate that the risk of cancer at specific sites is influenced by publication year, country, sex, and follow-up duration. Finally, we found that renal transplant recipients in studies published in 2010 or after have a greater risk of non-Hodgkin lymphoma than in those published before 2010. Renal transplant recipients in Eastern countries were found to have a higher risk of urinary bladder cancer, renal cell cancer, melanoma, and non-Hodgkin's lymphoma than those in Western countries. The pooled SIR ratio (female to male) was significantly high for hepatocellular carcinoma risk, and renal transplant recipients with longer follow-up durations displayed an increased risk of lip cancer than recipients with shorter follow-up durations.

A previous meta-analysis suggested that renal transplantation is associated with a significantly increased risk of bladder cancer [5], and that the risk of bladder cancer is lower among Europeans (SIR: 2.00) and greater among Asian (SIR: 14.74) ethnicities. However, the inherent limitation of this previous review is that analyses of cancer risks, according to study or patient characteristics, were not conducted. Furthermore, the SIR ratio between subgroups was not calculated, and therefore we lack knowledge pertaining to the role of publication year, ethnicity, sex, and follow-up duration on the risk of cancer at different sites. In our case, all included studies reported the SIR ratio of renal transplant recipients compared with the general population. In previous studies that failed to report relative risks, the non-exposed cohort may introduce uncontrolled biases, due to the inclusion of renal transplant recipients with cancer in the general population cohort. Therefore, we conducted this comprehensive meta-analysis to evaluate any potential role of renal transplantation on the risk of cancer, and to compare associated relationships according to study or patient characteristics.

Several mechanisms may explain the increased cancer risk for renal transplant recipients, and both viral and nonviral factors are involved in cancer progression after renal transplantation. Previous studies have demonstrated patients with kidney transplantations have a 3- to 4-fold increase in cancer risk [9, 20], and infection might contribute to the cancer development. Infection with hepatitis C virus, hepatitis B virus, and liver flukes are considered risk factors for biliary tract cancer [21, 22], and additionally, long-term immunosuppressive therapy, which is used after renal transplantation, may promote cancer at multiple sites [23–27]. We hypothesize that the underlying mechanism is most likely immunosuppressive therapy, as this contributes a direct role on cellular damage and impairs the ability of the body to repair damage to cellular DNA or destroy damaged cells [28]. However, we noted a lack of correlation between renal transplant and prostate or uterine cancers, and we suspect that this could be attributed to the strength of surveillance for cancers, and these 2 cancers are commonly diagnosed by opportunistic screening. Furthermore, these conclusions may be variable and require large-scale prospective studies to verify our observed relationships.

Our subgroup analyses suggest that renal transplant is associated with an increased risk of urinary bladder cancer, renal cell cancer, melanoma, and non-Hodgkin's lymphoma in Eastern countries. Possible reason for this observation could include different genetic backgrounds and environmental factors. Furthermore, women who received renal transplantation have a greater risk of hepatocellular carcinoma than in men. Our study included patients with a mean age of approximately 40.0 years, and hormone replacements and oophorectomies might affect the incidence of hepatocellular carcinoma [29]. Recipients with longer-follow-up durations have a higher risk of lip cancer than those with shorter follow-up durations. This effect might be attributable to the long-term use of immunosuppressive therapies, which play important roles in the progression of cancer at multiple sites.

Renal transplantation correlates with a higher risk of various cancers, with the exception of uterine and prostate cancers, and our findings persist across most cancers in patients with specific characteristics. Furthermore, although potential heterogeneity exists among the included studies, our sensitivity analyses, through sequentially excluding each study from the overall analysis, revealed that our observations remain stable, except in melanoma and breast cancer cases. The variable findings of melanoma and breast cancers could be due to a small number of cohorts. Finally, the cancer risks in renal transplant recipients have stratified in our study across patients with specific characteristics, and we suggest that screening strategies for specific cancers should be modified for renal transplant recipients with the greatest cancer risk.

Three strengths of our study should be highlighted. First, only prospective cohort studies were included, which eliminates the recall bias associated with retrospective studies. Second, the large sample size allowed us to quantitatively assess the impact of renal transplantation on the risk of cancer at multiple sites, and thus, our findings are more robust than those of any individual study. Third, the SIR ratio was calculated between subgroups, which allows evaluation of the impact of renal transplants within specific populations.

We acknowledge several limitations in regards to our meta-analysis. First, several important factors, including mean patient age, body mass index [30], immunosuppressive drugs [31], diabetes mellitus [32], alcohol use [33], smoking status [34], and renal source may correlate with cancer development at multiple sites, although these factors were not available in detail in most of the included studies. Second, publication bias is inevitable since the included studies were published, and third, summary results of the pooled and individual data were not available.

In conclusion, renal transplant recipients display a greater risk of cancer at multiple sites with the exception of uterine and prostate cancers. Our observations may be influenced by publication year, country, sex, and follow-up duration, and future studies are required to clarify the interactions of multiple confounding risk factors and investigate the interaction of renal transplantation with these factors and subsequent cancer risks.

## MATERIALS AND METHODS

### Data sources, search strategy, and selection criteria

This study was conducted and reported according to the Meta-analysis of Observational Studies in Epidemiology protocol [35]. Relevant prospective cohort studies that evaluated cancer risks in renal transplant recipients were identified. Briefly, we searched PubMed, Embase, and the Cochrane library for studies published up to July 2, 2017 using the following search terms: “renal” OR “kidney” AND “transplantation” AND “cancer” AND “cohort”, without language and publication status restrictions. All analyses were based on previously published studies, and as such, no ethical approvals or patient consents were required. Manual searches were also conducted to identify additional studies.

Studies that we included in our meta-analysis met the following criteria: (1) The study had a prospective cohort design, (2) all participants ≥ 18 years old and received renal transplantation, (3) the study reported at least 1 of the following cancer risks in renal transplant recipients: all cancer, gastric cancer, colon cancer, pancreatic cancer, hepatocellular carcinoma, lung cancer, thyroid cancer, urinary bladder cancer, renal cell cancer, non-melanoma skin cancer, melanoma, Hodgkin's lymphoma, non-Hodgkin's lymphoma, lip cancer, breast cancer, ovarian cancer, uterine cancer, and prostate cancer. Studies were excluded for the following reasons: (1) Participants received another solid organ transplant without renal transplantation, (2) follow-up time of < 3.0 years, and (3) lack of available data with appropriate statistics.

### Data extraction and quality assessment

The following data was independently extracted from each study, by two authors, and inconsistencies were settled by group discussion. The following information was collected: first author's name, year of publication, country, type of transplant, mean or median age, sample size, number of renal transplant cases, number of all cancers, reported outcomes, and follow-up durations. The quality of the trials was assessed according to the 9-star system Newcastle-Ottawa Scale [36], which includes selection (4 items), comparability (1 item), and outcome (3 items). Quality assessments were conducted by 2 authors and adjudicated by a third author when disagreements occurred.

### Statistical analysis

The meta-analysis was conducted using STATA Software (version 10.0; Stata Corporation, College Station, TX, USA). We examined cancer risk in renal transplant recipients on the basis of the standard incidence ratio (SIR) and its 95% confidence interval (CI) published in each study. We used the random-effects model to calculate SIRs and 95% CIs for renal transplant recipients versus the general population and to determine the risk of all cancer, gastric cancer, colon cancer, pancreatic cancer, hepatocellular carcinoma, lung cancer, thyroid cancer, urinary bladder cancer, renal cell cancer, non-melanoma skin cancer, melanoma, Hodgkin's lymphoma, non-Hodgkin's lymphoma, lip cancer, breast cancer, ovarian cancer, uterine cancer, and prostate cancer [37, 38]. Potential heterogeneity across studies was examined using the Cochran's Q-statistic [39] and I^2^ statistic [40]. Heterogeneity *P*-values < 0.05 or I^2^ > 50% indicates significant heterogeneity across respective studies.

Sensitivity analyses were conducted for the risk of cancer at different sites, to investigate the influence of a single study in the meta-analysis on the overall risk [41]. To explore potential associations among studies with different characteristics, a subgroup analysis was conducted according to publication year (2010 or after, before 2010), country (Eastern, Western), sex (male, female), and follow-up duration (8.0 years or greater, < 8.0 years) for each specific cancer, which allowed effect estimations in specific subpopulations. Furthermore, SIR ratios and corresponding 95% CIs between subgroups were also calculated. Publication bias was evaluated using funnel plots, Egger [42], and Begg's test [43], and *P* < 0.05 represents a statistically significant publication bias.

## SUPPLEMENTARY MATERIALS FIGURES AND TABLES



## References

[1] Suthanthiran M, Strom TB (1994). Renal transplantation. N Engl J Med.

[2] Wolfe RA, Ashby VB, Milford EL, Ojo AO, Ettenger RE, Agodoa LY, Held PJ, Port FK (1999). Comparison of mortality in all patients on dialysis, patients on dialysis awaiting transplantation, and recipients of a first cadaveric transplant. N Engl J Med.

[3] Zhu X, Wang JZ, Zhang Y, Xu M, Chen P, Wang CZ (2016). Risk of renal cancer in liver transplant recipients: A systematic review and meta-analysis. Int J Surg.

[4] Ulrich C, Arnold R, Frei U, Hetzer R, Neuhaus P, Stockfleth E (2014). Skin changes following organ transplantation: an interdisciplinary challenge. Dtsch Arztebl Int.

[5] Yan L, Chen P, Chen EZ, Gu A, Jiang ZY (2014). Risk of bladder cancer in renal transplant recipients: a meta-analysis. Br J Cancer.

[6] Karamchandani D, Arias-Amaya R, Donaldson N, Gilbert J, Schulte KM (2010). Thyroid cancer and renal transplantation: a meta-analysis. Endocr Relat Cancer.

[7] Zeier M, Hartschuh W, Wiesel M, Lehnert T, Ritz E (2002). Malignancy after renal transplantation. Am J Kidney Dis.

[8] Hoshida Y, Tsukuma H, Yasunaga Y, Xu N, Fujita MQ, Satoh T, Ichikawa Y, Kurihara K, Imanishi M, Matsuno T, Aozasa K (1997). Cancer risk after renal transplantation in Japan. Int J Cancer.

[9] Birkeland SA, Løkkegaard H, Storm HH (2000). Cancer risk in patients on dialysis and after renal transplantation. Lancet.

[10] Kyllönen L, Salmela K, Pukkala E (2000). Cancer incidence in a kidney-transplanted population. Transpl Int.

[11] Vajdic CM, McDonald SP, McCredie MR, van Leeuwen MT, Stewart JH, Law M, Chapman JR, Webster AC, Kaldor JM, Grulich AE (2006). Cancer Incidence Before and After Kidney Transplantation. JAMA.

[12] Végso G, Tóth M, Hídvégi M, Toronyi E, Langer RM, Dinya E, Tóth A, Perner F, Járay J (2007). Malignancies after Renal Transplantation during 33 Years at a Single Center. Pathol Oncol Res.

[13] Villeneuve PJ, Schaubel DE, Fenton SS, Shepherd FA, Jiang Y, Mao Y (2007). Cancer Incidence Among Canadian Kidney Transplant Recipients. Am J Transplant.

[14] Collett D, Mumford L, Banner NR, Neuberger J, Watson C (2010). Comparison of the Incidence of Malignancy in Recipients of Different Types of Organ: A UK Registry Audit. Am J Transplant.

[15] Li WH, Chen YJ, Tseng WC, Lin MW, Chen TJ, Chu SY, Hwang CY, Chen CC, Lee DD, Chang YT, Wang WJ, Liu HN (2012). Malignancies after renal transplantation in Taiwan: a nationwide population-based study. Nephrol Dial Transplant.

[16] Cheung CY, Lam MF, Chu KH, Chow KM, Tsang KY, Yuen SK, Wong PN, Chan SK, Leung KT, Chan CK, Ho YW, Chau KF (2012). Malignancies after kidney transplantation: Hong Kong renal registry. Am J Transplant.

[17] Krynitz B, Edgren G, Lindelof B, Baecklund E, Brattström C, Wilczek H, Smedby KE (2013). Risk of skin cancer and other malignancies in kidney, liver, heart and lung transplant recipients 1970 to 2008—A Swedish population-based study. Int J Cancer.

[18] Piselli P, Serraino D, Segoloni GP, Sandrini S, Piredda GB, Scolari MP, Rigotti P, Busnach G, Messa P, Donati D, Schena FP, Maresca MC, Tisone G (2013). Risk ofde novocancers after transplantation: Results from a cohort of 7217 kidney transplant recipients, Italy 1997–2009. Eur J Cancer.

[19] Duvall S, Tweedie R (2000). A nonparametric “trim and fill” method for assessing publication bias in meta-analysis. J Am Stat Assoc.

[20] Birkeland SA, Storm JJ, Lamm LU, Barlow L, Blohmé I, Forsberg B, Eklund B, Fjeldborg O, Friedberg M, Frödin L (1995). Cancer risk after renal transplantation in the Nordic countries, 1964–1986. Int J Cancer.

[21] Shaib Y, el-Serag HB (2004). The epidemiology of cholangiocarcinoma. Semin Liver Dis.

[22] International Agency for Research on Cancer (IARC) (1994). IARC Monographs on the Evaluation of Carcinogenic Risks to Humans: Schistosomes, Liver Flukes and Helicobacter pylori.

[23] Heijl C, Harper L, Flossmann O, Stücker I, Scott DG, Watts RA, Höglund P, Westman K, Mahr A, European Vasculitis Study Group (EUVAS) (2011). Incidence of malignancy in patients treated for antineutrophil cytoplasm antibody-associated vasculitis: follow-up data from European Vasculitis Study Group clinical trials. Ann Rheum Dis.

[24] Penninga L, Penninga EI, Moller CH, Iversen M, Steinbrüchel DA, Gluud C (2013). Tacrolimus versus cyclosporin as primary immunosuppression for lung transplant recipients. Cochrane Database Syst Rev.

[25] Penninga L, Moller CH, Penninga EI, Iversen M, Gluud C, Steinbrüchel DA (2013). Antibody induction therapy for lung transplant recipients. Cochrane Database Syst Rev.

[26] Penninga L, Moller CH, Gustafsson F, Gluud C, Steinbrüchel DA (2013). Immunosuppressive T-cell antibody induction for heart transplant recipients. Cochrane Database Syst Rev.

[27] Tian SY, Feldman BM, Beyene J, Brown PE, Uleryk EM, Silverman ED (2014). Immunosuppressive therapies for the induction treatment of proliferative lupus nephritis: a systematic review and network metaanalysis. J Rheumatol.

[28] Buzzeo BD, Heisey DM, Messing EM (1997). Bladder cancer in renal transplant recipients. Urology.

[29] McGlynn KA, Sahasrabuddhe VV, Campbell PT, Graubard BI, Chen J, Schwartz LM, Petrick JL, Alavanja MC, Andreotti G, Boggs DA, Buring JE, Chan AT, Freedman ND (2015). Reproductive factors, exogenous hormone use and risk of hepatocellular carcinoma among US women: results from the Liver Cancer Pooling Project. Br J Cancer.

[30] Freisling H, Arnold M, Soerjomataram I, O’Doherty MG, Ordóñez-Mena JM, Bamia C, Kampman E, Leitzmann M, Romieu I, Kee F, Tsilidis K, Tjønneland A, Trichopoulou A (2017). Comparison of general obesity and measures of body fat distribution in older adults in relation to cancer risk: meta-analysis of individual participant data of seven prospective cohorts in Europe. Br J Cancer.

[31] Yanik EL, Siddiqui K, Engels EA (2015). Sirolimus effects on cancer incidence after kidney transplantation: a meta-analysis. Cancer Med.

[32] Sasazuki S, Charvat H, Hara A, Wakai K, Nagata C, Nakamura K, Tsuji I, Sugawara Y, Tamakoshi A, Matsuo K, Oze I, Mizoue T, Tanaka K (2013). Diabetes mellitus and cancer risk: pooled analysis of eight cohort studies in Japan. Cancer Sci.

[33] Bagnardi V, Rota M, Botteri E, Tramacere I, Islami F, Fedirko V, Scotti L, Jenab M, Turati F, Pasquali E, Pelucchi C, Galeone C, Bellocco R (2015). Alcohol consumption and site-specific cancer risk: a comprehensive dose-response meta-analysis. Br J Cancer.

[34] Ordóñez-Mena JM, Schöttker B, Mons U, Jenab M, Freisling H, Bueno-de-Mesquita B, O’Doherty MG, Scott A, Kee F, Stricker BH, Hofman A, de Keyser CE, Ruiter R (2016). Quantification of the smoking-associated cancer risk with rate advancement periods: meta-analysis of individual participant data from cohorts of the CHANCES consortium. BMC Med.

[35] Stroup DF, Berlin JA, Morton SC, Olkin I, Williamson GD, Rennie D, Moher D, Becker BJ, Sipe TA, Thacker SB, Meta-analysis Of Observational Studies in Epidemiology (MOOSE) group (2000). Meta-analysis of observational studies in epidemiology: a proposal for reporting. JAMA.

[36] Wells GA, Shea BJ, O’Connell D, Robertson J, Peterson J, Welch V, Losos M, Tugwell P (2014). The Newcastle–Ottawa Scale (NOS) for Assessing the Quality of Non-Randomized Studies in Meta-Analysis. Applied Engineering in Agriculture.

[37] DerSimonian R, Laird N (1986). Meta-analysis in clinical trials. Control Clin Trials.

[38] Ades AE, Lu G, Higgins JP (2005). The interpretation of random-effects metaanalysis in decision models. Med Decis Making.

[39] Lau J, Ioannidis JP, Schmid CH (1997). Quantitative synthesis in systematic reviews. Ann Intern Med.

[40] Higgins JP, Thompson SG, Deeks JJ, Altman DG (2003). Measuring inconsistency in meta-analyses. BMJ.

[41] Tobias A (1999). Assessing the influence of a single study in meta-analysis. Stata Tech Bull.

[42] Egger M, Davey Smith G, Schneider M, Minder C (1997). Bias in meta-analysis detected by a simple, graphical test. BMJ.

[43] Begg CB, Mazumdar M (1994). Operating characteristics of a rank correlation test for publication bias. Biometrics.

