# Supplementary material for: Cancer risks in recipients of renal transplants: a meta-analysis of cohort studies
Source: Oncotarget. 2017 Dec 16;9(20):15375–85. doi: 10.18632/oncotarget.23841 (PMC5880611; doi:10.18632/oncotarget.23841)
Supplement: Supplementary file 1 [file oncotarget-09-15375-s001.pdf]

Cancer risks in recipients of renal transplants: a meta-analysis of cohort studies

SUPPLEMENTARY MATERIALS

Supplementary 1

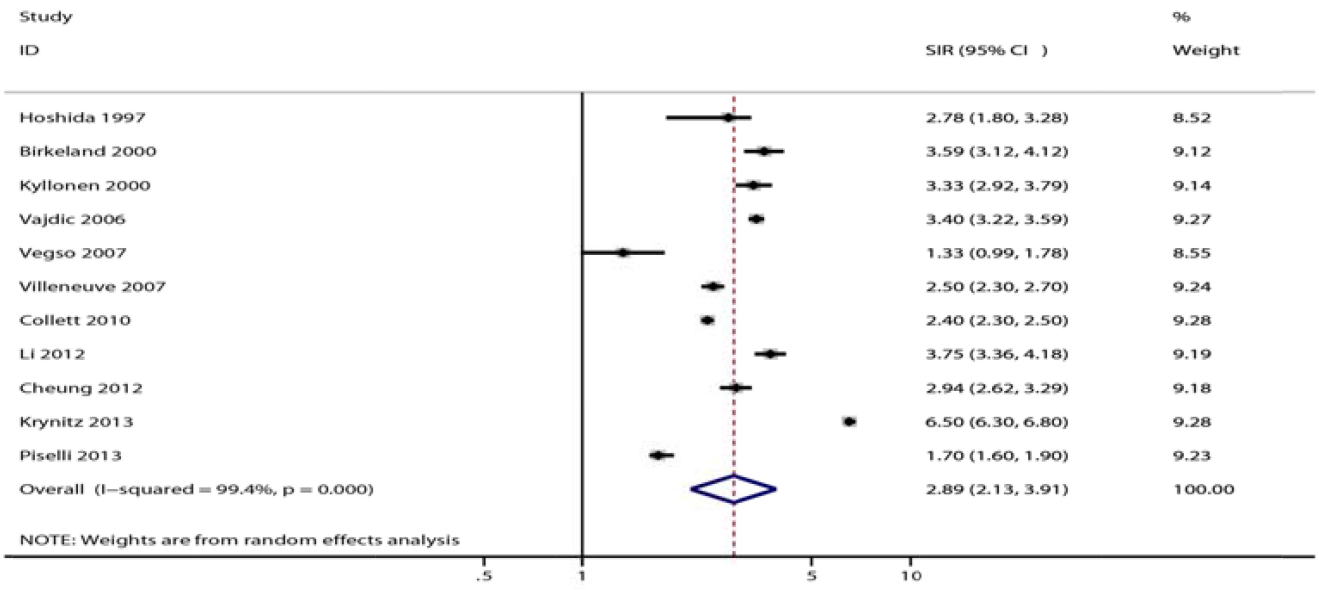

Supplementary Figure 1: All cancer risk in renal transplant recipients.

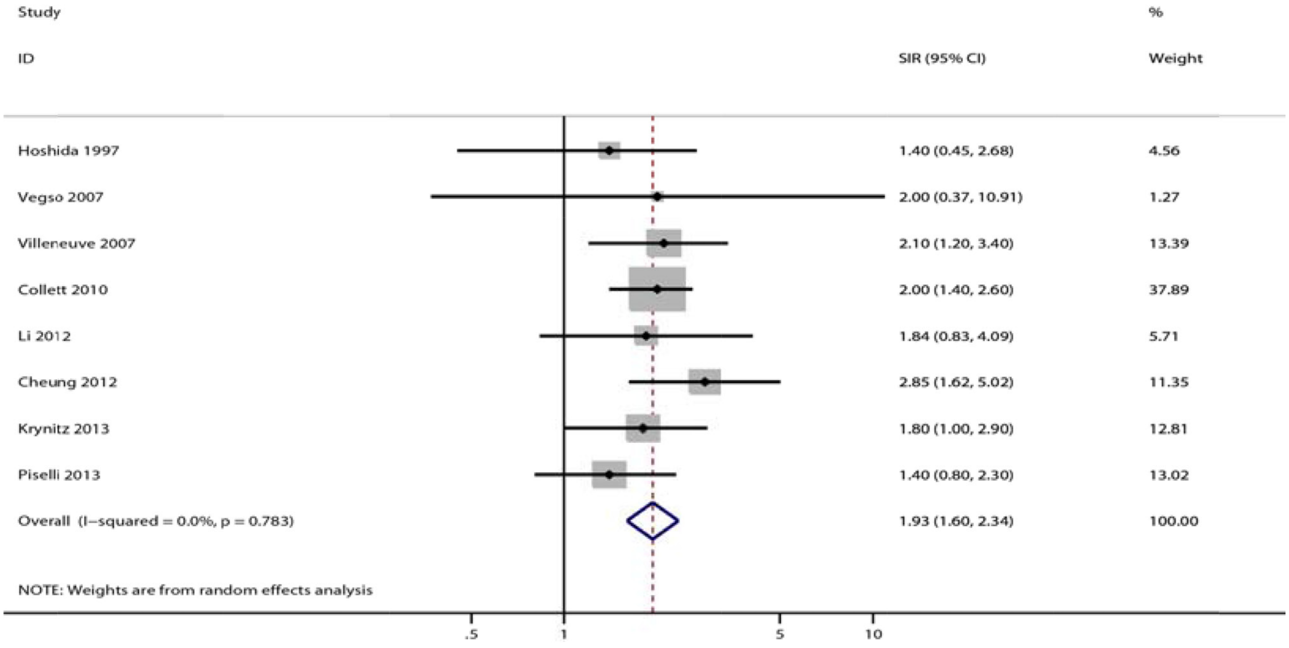

Supplementary Figure 2: Gastric cancer risk in renal transplant recipients.

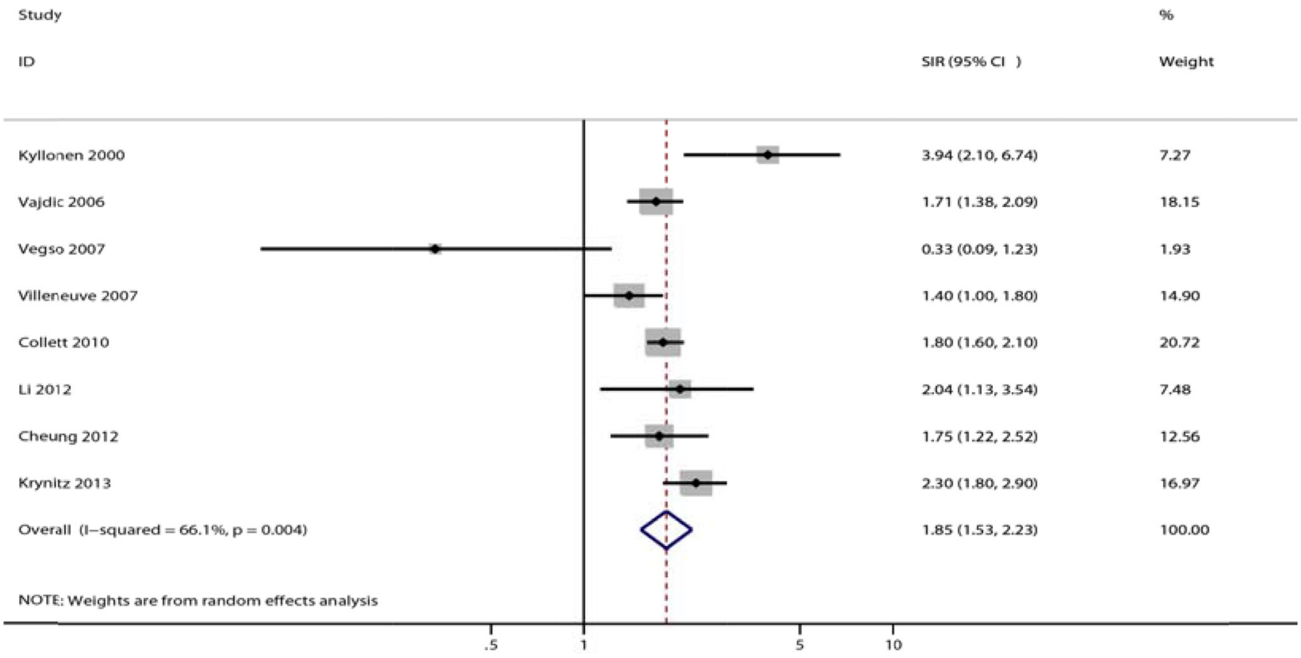

Supplementary Figure 3: Colon cancer risk in renal transplant recipients.

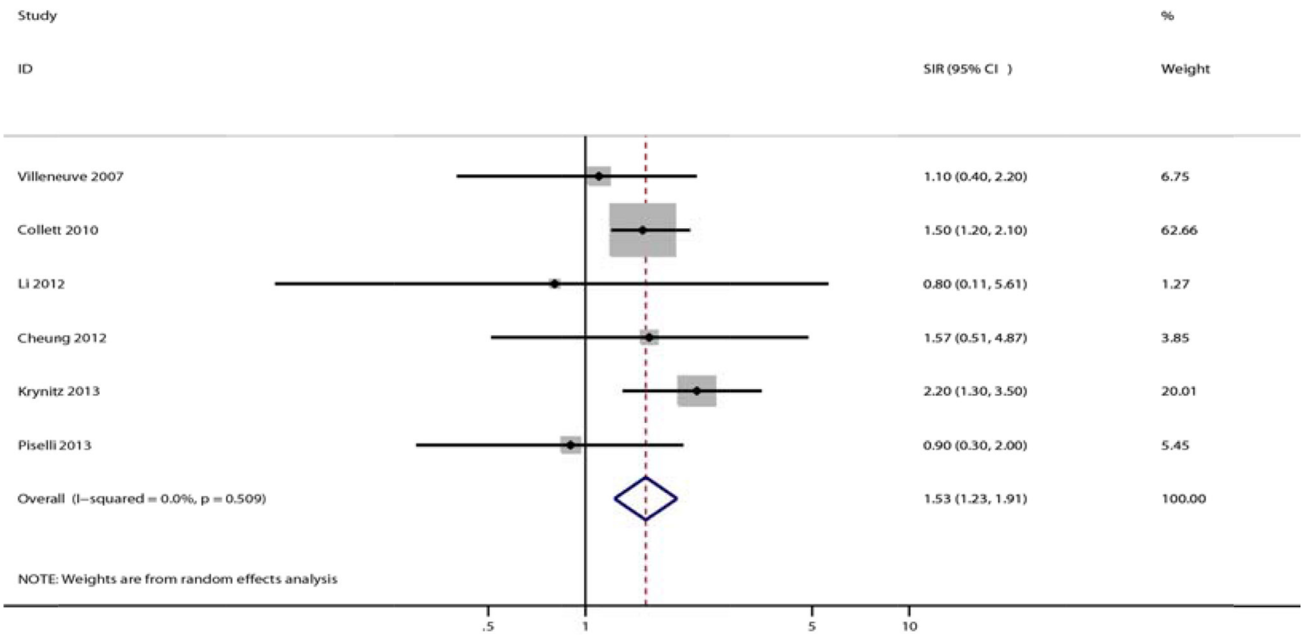

Supplementary Figure 4: Pancreatic cancer risk in renal transplant recipients.

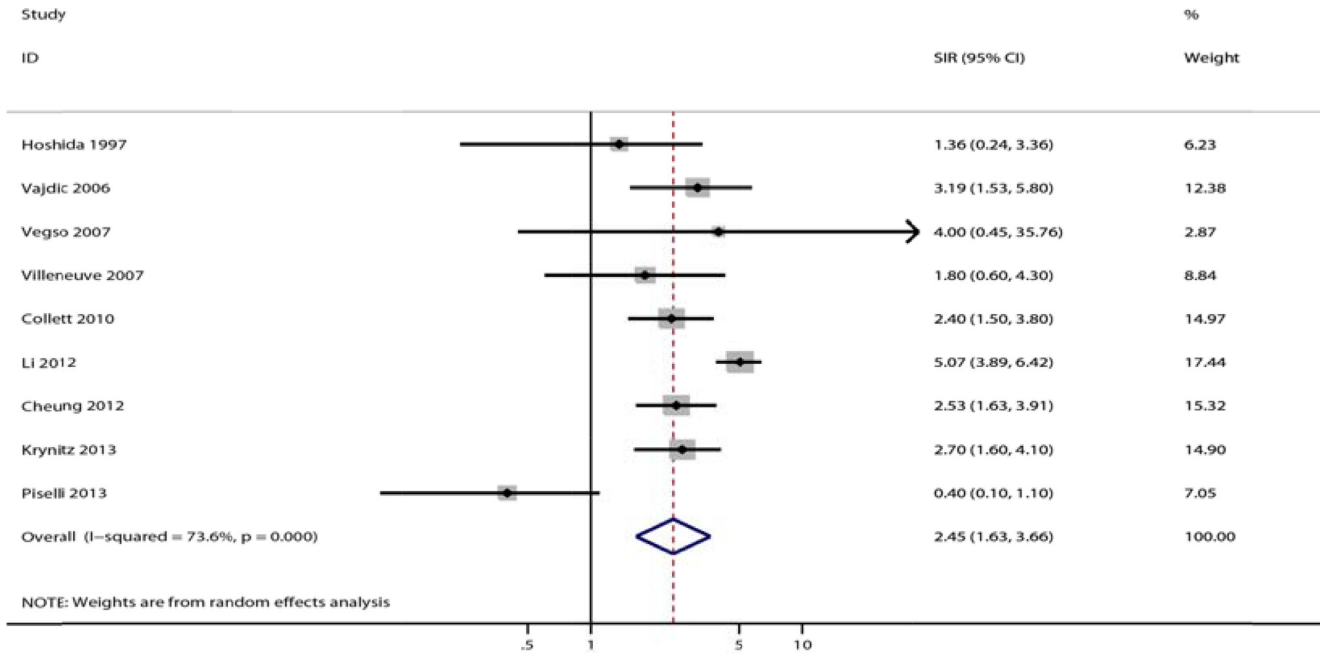

Supplementary Figure 5: Hepatocellular carcinoma risk in renal transplant recipients.

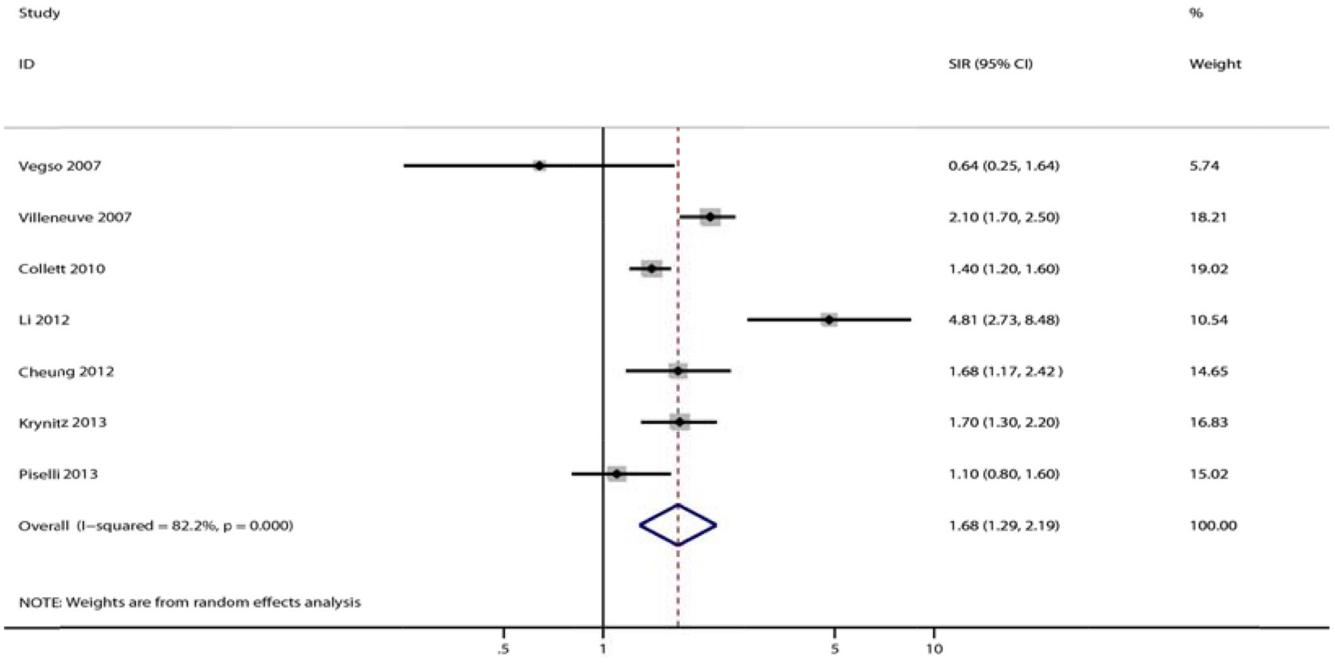

Supplementary Figure 6: Lung cancer risk in renal transplant recipients.

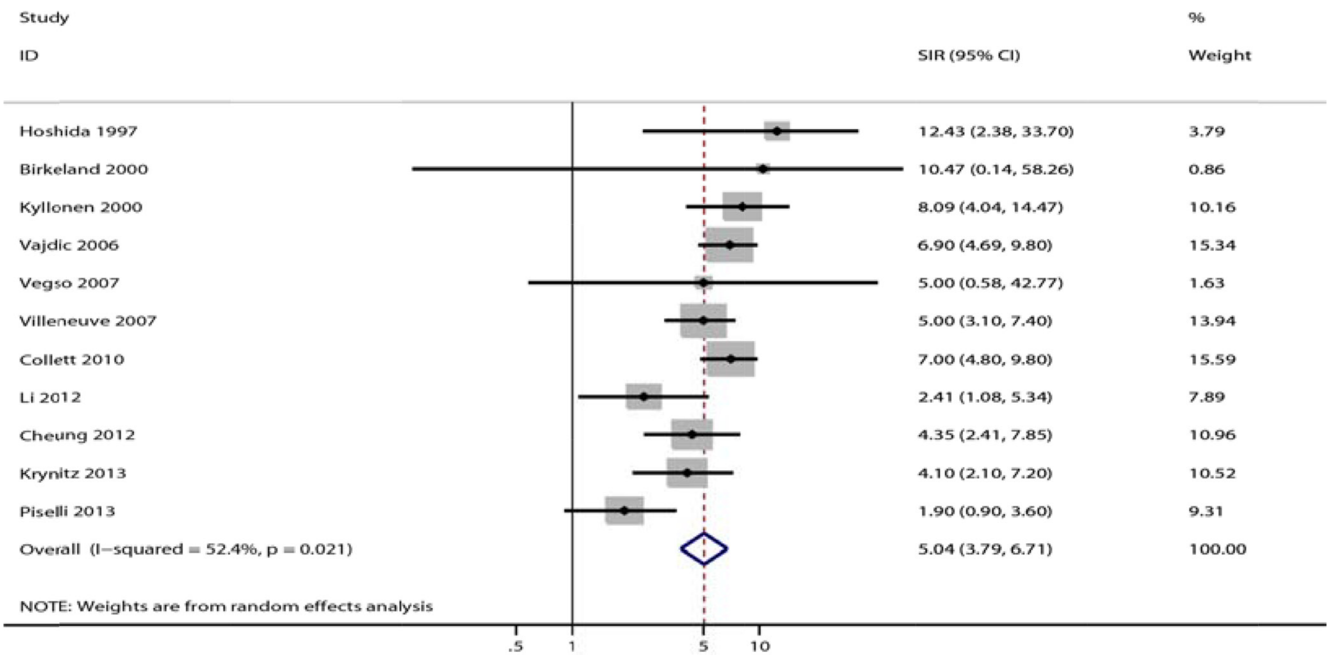

Supplementary Figure 7: Thyroid cancer risk in renal transplant recipients.

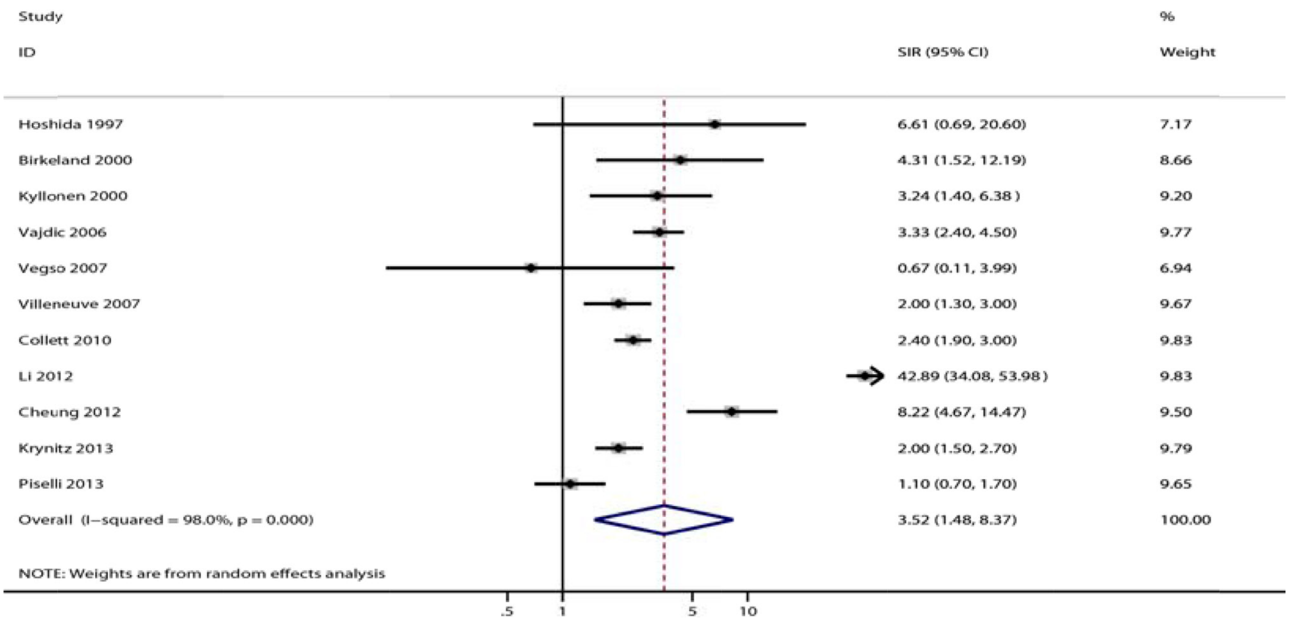

Supplementary Figure 8: Urinary bladder cancer risk in renal transplant recipients.

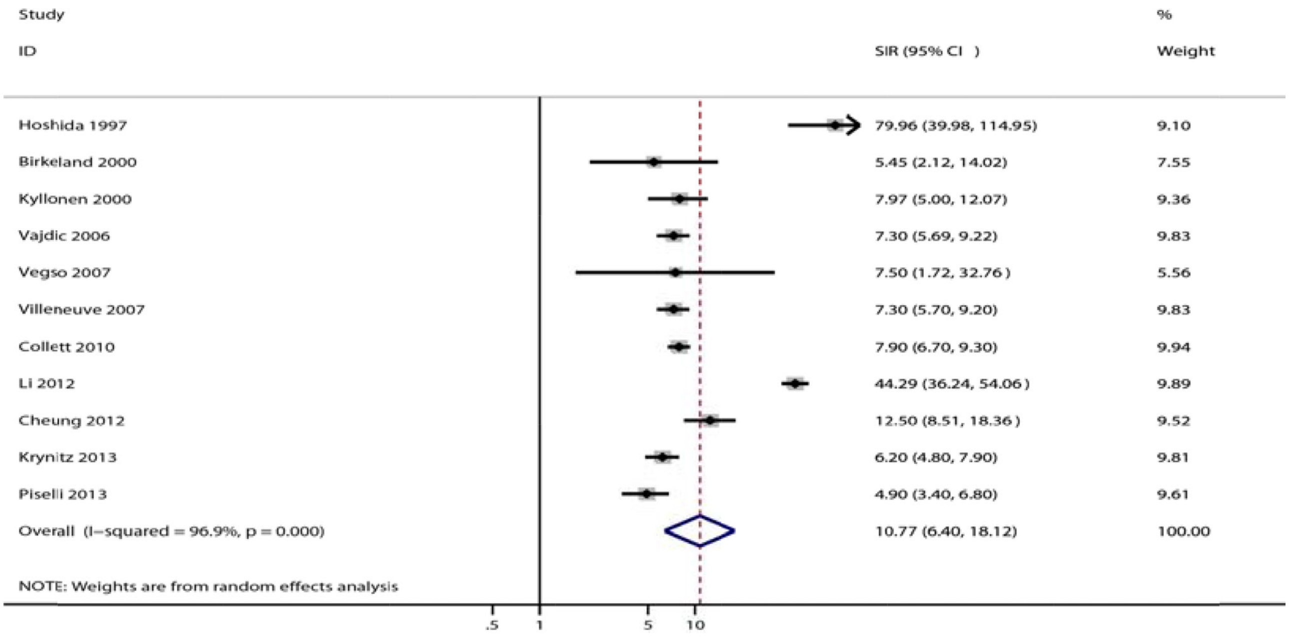

Supplementary Figure 9: Renal cell cancer risk in renal transplant recipients.

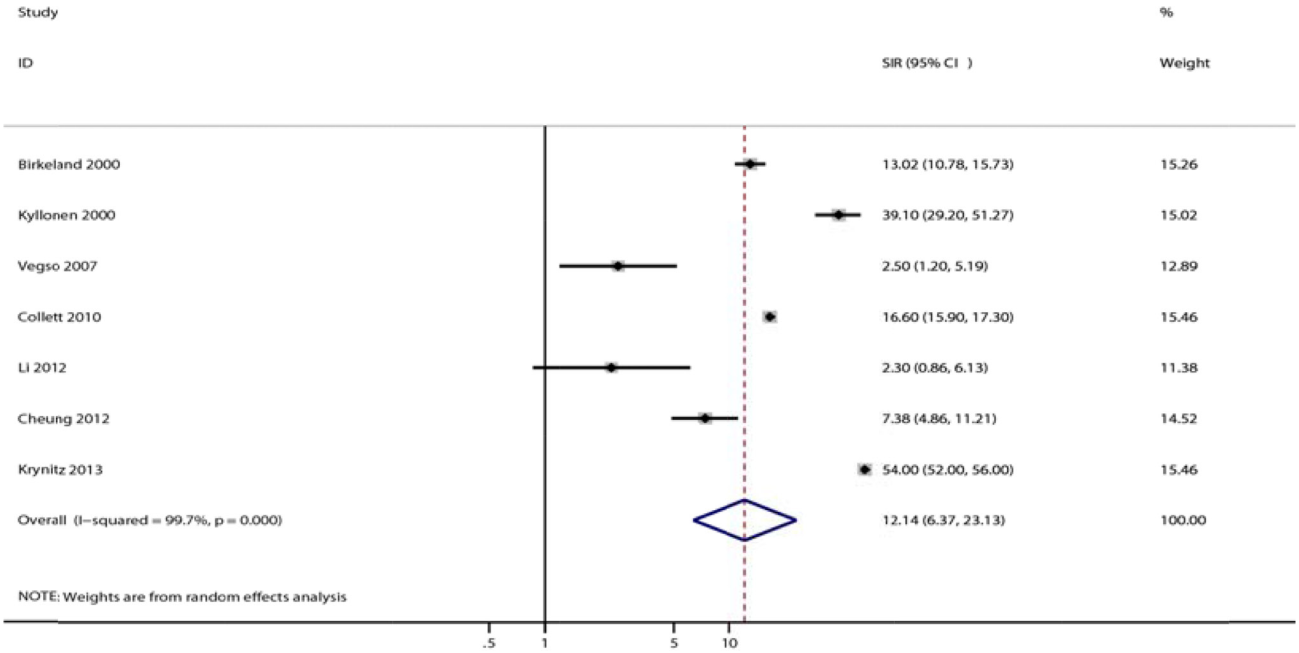

Supplementary Figure 10: Non-melanoma skin cancer risk in renal transplant recipients.

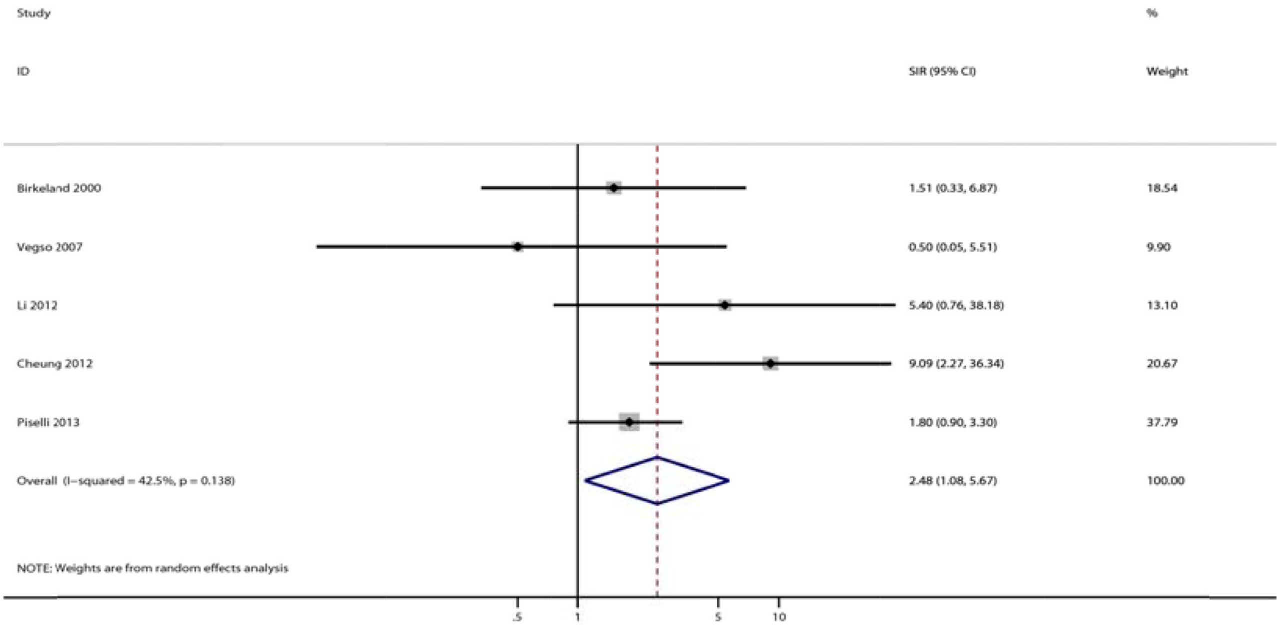

Supplementary Figure 11: Melanoma risk in renal transplant recipients.

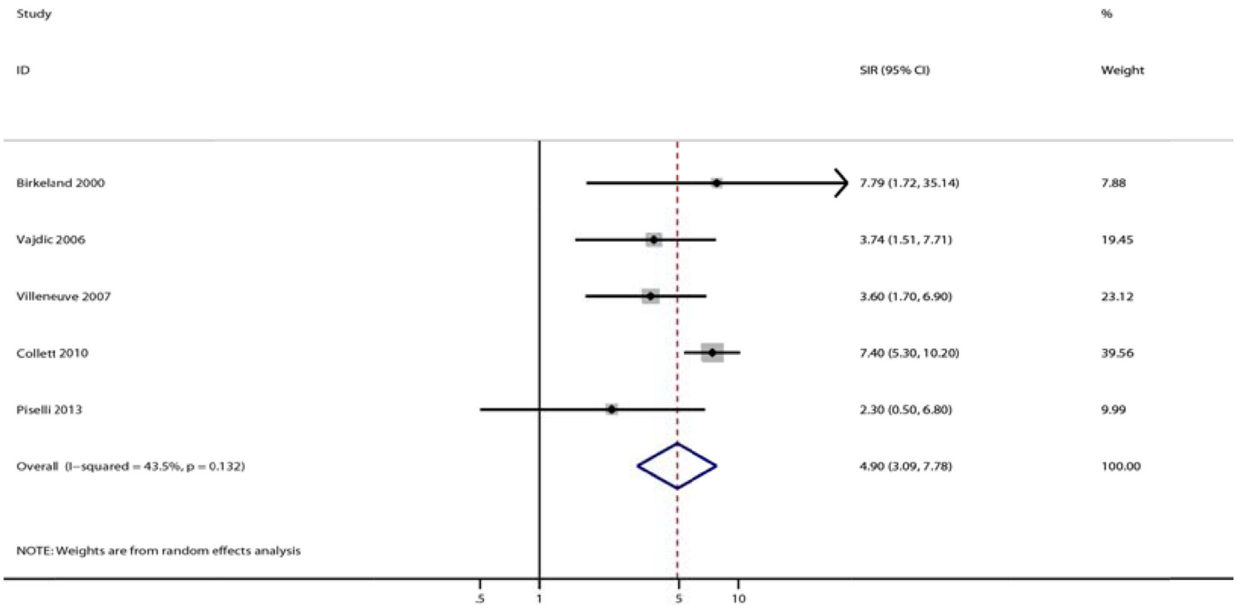

Supplementary Figure 12: Hodgkin’s lymphoma risk in renal transplant recipients.

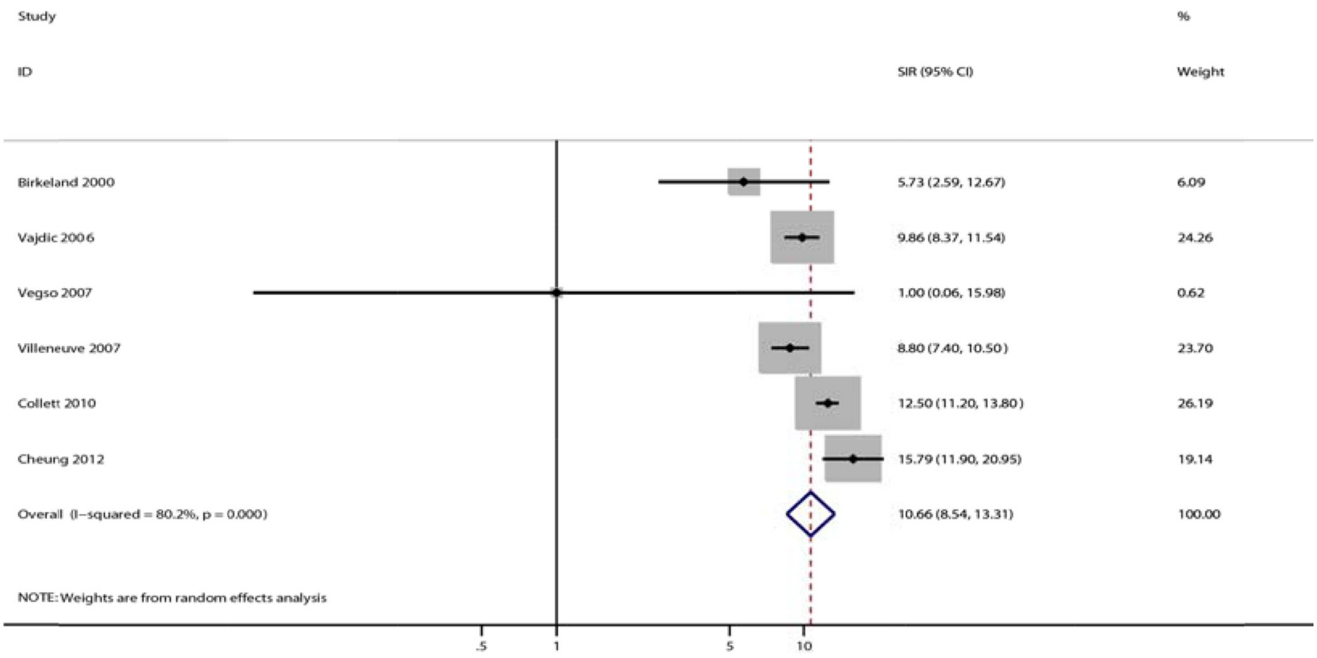

Supplementary Figure 13: Non-Hodgkin lymphoma risk in renal transplant recipients.

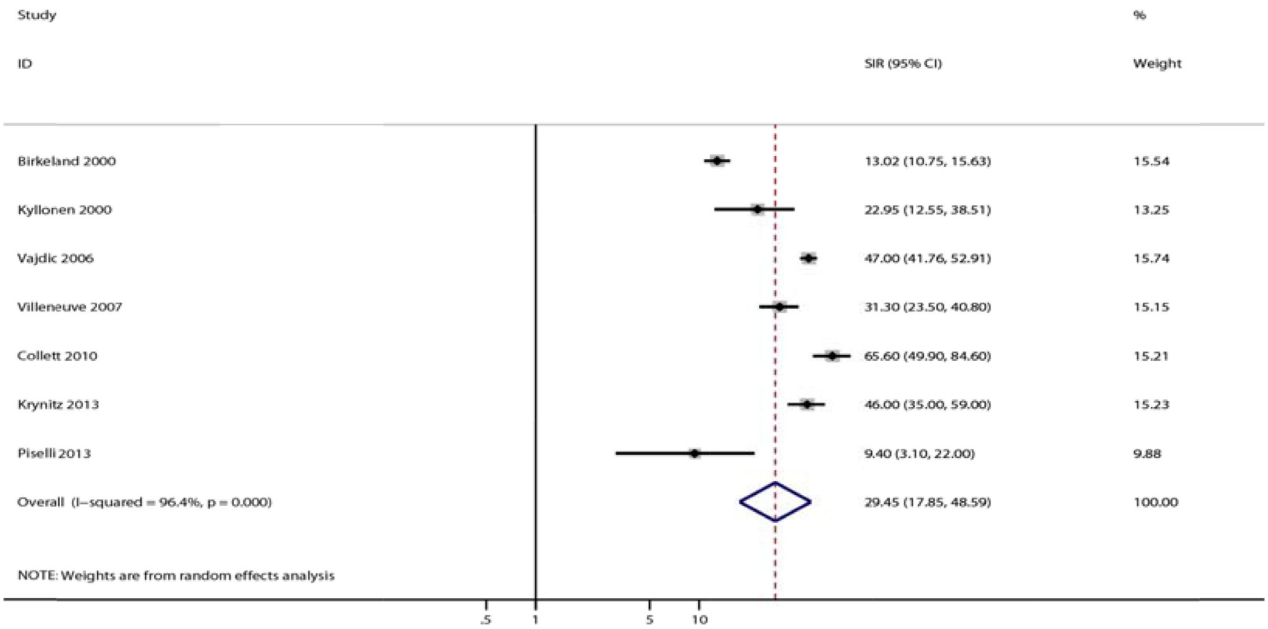

Supplementary Figure 14: Lip cancer risk in renal transplant recipients.

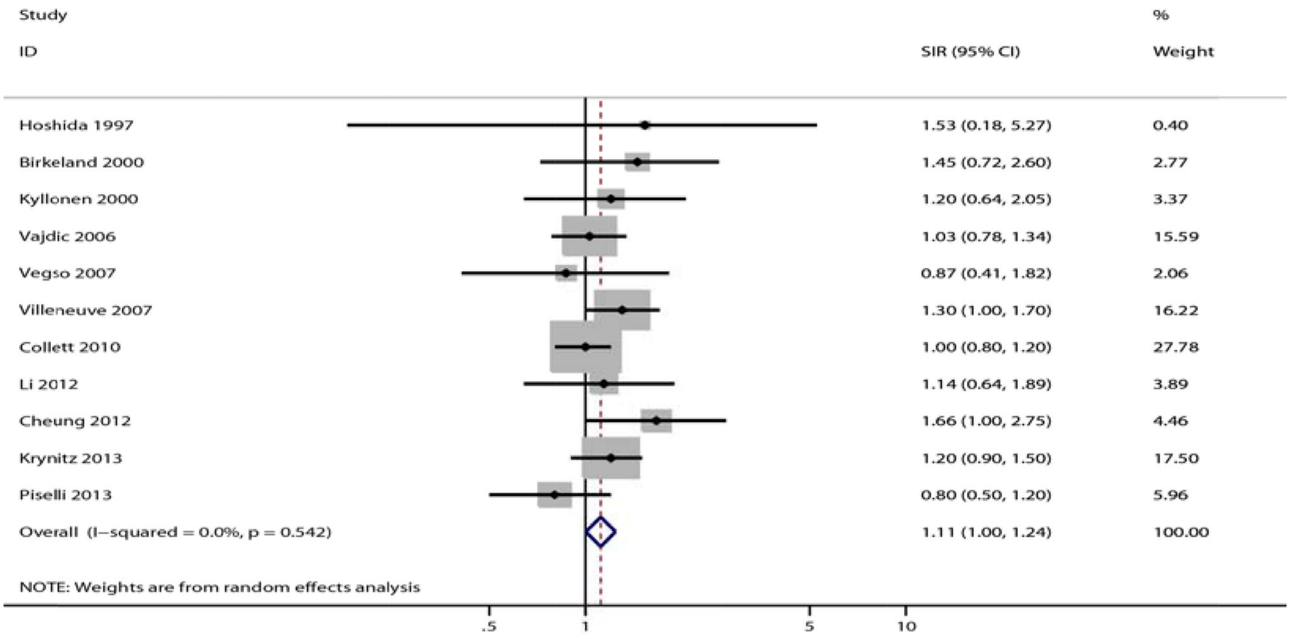

Supplementary Figure 15: Breast cancer risk in renal transplant recipients.

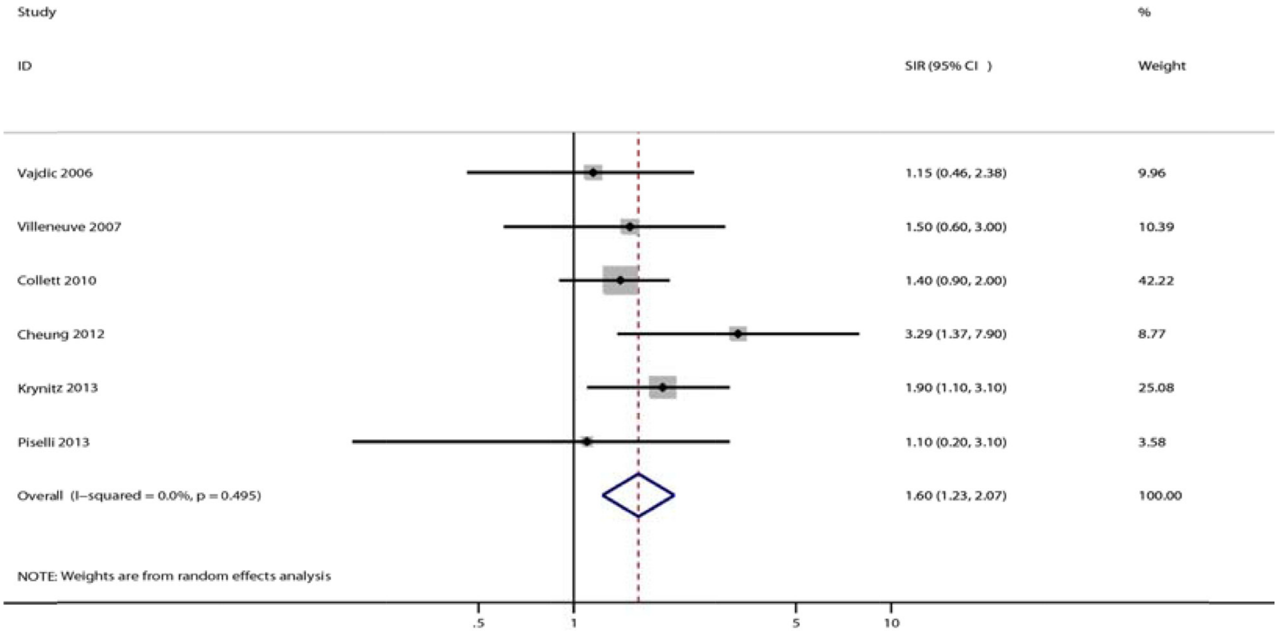

Supplementary Figure 16: Ovarian cancer risk in renal transplant recipients.

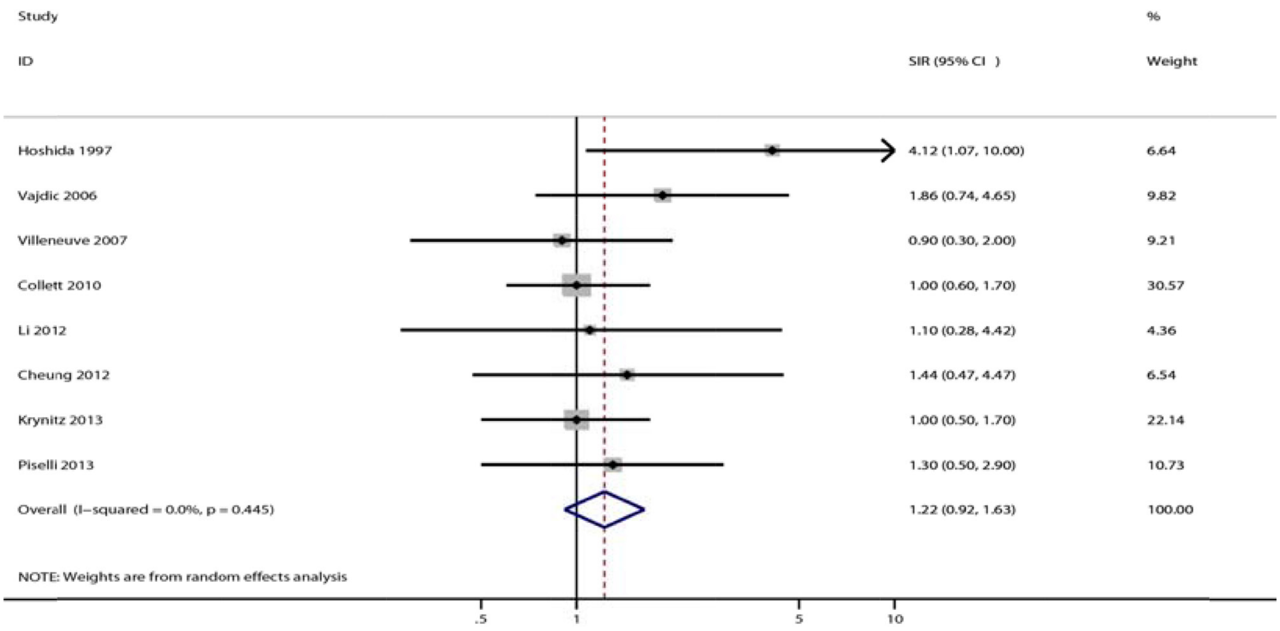

Supplementary Figure 17: Uterus cancer risk in renal transplant recipients.

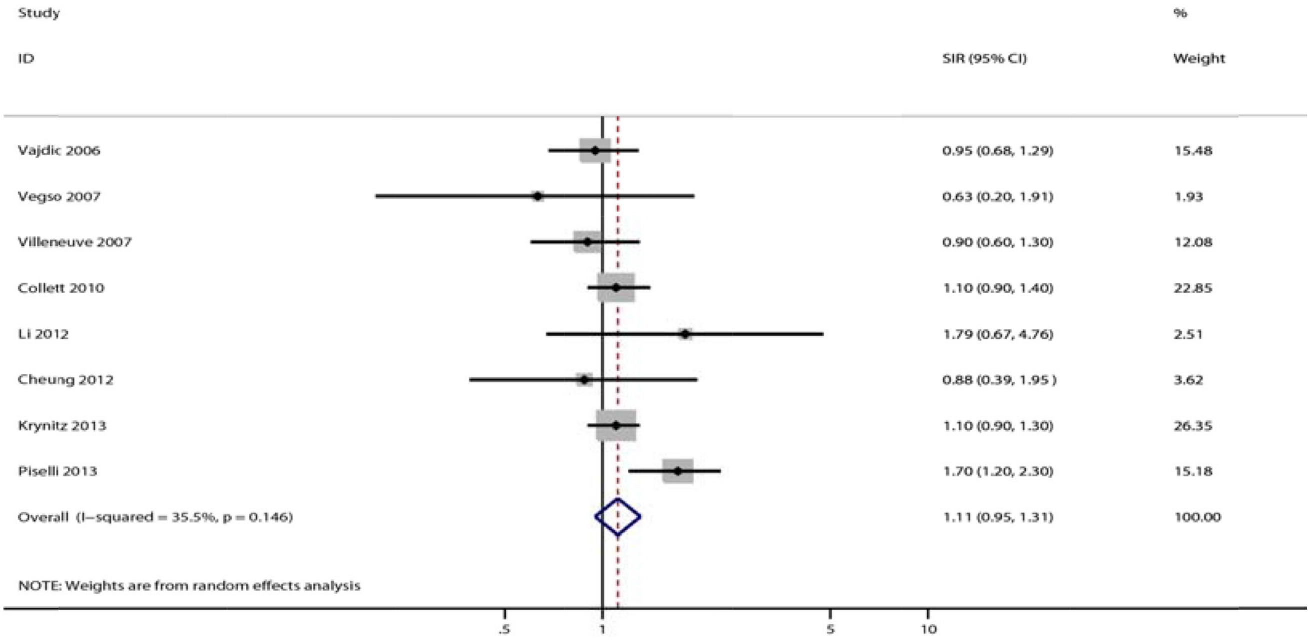

Supplementary Figure 18: Prostate cancer risk in renal transplant recipients.

Supplementary 2

Supplementary Table 1: The details of sensitivity analysis for all cancers

Excluding study	SIR and 95% CI	*P* value	Heterogeneity (%)	*P* value for heterogeneity
Hoshida 1997	2.90 (2.11–3.98)	< 0.001	99.5	< 0.001
Birkeland 2000	2.83 (2.05–3.90)	< 0.001	99.5	< 0.001
Kyllonen 2000	2.85 (2.06–3.93)	< 0.001	99.5	< 0.001
Vajdic 2006	2.84 (1.99–4.05)	< 0.001	99.5	< 0.001
Vegso 2007	3.11 (2.27–4.25)	< 0.001	99.5	< 0.001
Villeneuve 2007	2.93 (2.11–4.06)	< 0.001	99.5	< 0.001
Collett 2010	2.94 (2.11–4.08)	< 0.001	99.3	< 0.001
Li 2012	2.81 (2.03–3.90)	< 0.001	99.5	< 0.001
Cheung 2012	2.88 (2.08–3.99)	< 0.001	99.5	< 0.001
Krynitz 2013	2.68 (2.27–3.16)	< 0.001	97.0	< 0.001
Piselli 2013	3.05 (2.25–4.13)	< 0.001	99.4	< 0.001

Supplementary Table 2: The details of sensitivity analysis for gastric cancer

Excluding study	SIR and 95% CI	*P* value	Heterogeneity (%)	*P* value for heterogeneity
Hoshida 1997	1.96 (1.61–2.39)	< 0.001	0.0	0.751
Vegso 2007	1.93 (1.59–2.34)	< 0.001	0.0	0.680
Villeneuve 2007	1.91 (1.55–2.34)	< 0.001	0.0	0.695
Collett 2010	1.89 (1.49–2.41)	< 0.001	0.0	0.690
Li 2012	1.94 (1.59–2.36)	< 0.001	0.0	0.682
Cheung 2012	1.84 (1.50–2.25)	< 0.001	0.0	0.926
Krynitz 2013	1.95 (1.59–2.40)	< 0.001	0.0	0.691
Piselli 2013	2.03 (1.65–2.49)	< 0.001	0.0	0.887

Supplementary Table 3: The details of sensitivity analysis for colon cancer

Excluding study	SIR and 95% CI	*P* value	Heterogeneity (%)	*P* value for heterogeneity
Kyllonen 2000	1.76 (1.49–2.07)	< 0.001	56.4	0.032
Vajdic 2006	1.87 (1.48–2.38)	< 0.001	70.2	0.003
Vegso 2007	1.89 (1.61–2.22)	< 0.001	57.2	0.029
Villeneuve 2007	1.94 (1.58–2.37)	< 0.001	65.0	0.009
Collett 2010	1.85 (1.43–2.40)	< 0.001	70.8	0.002
Li 2012	1.83 (1.49–2.25)	< 0.001	70.7	0.002
Cheung 2012	1.86 (1.50–2.31)	< 0.001	70.8	0.002
Krynitz 2013	1.76 (1.43–2.17)	< 0.001	63.4	0.012

Supplementary Table 4: The details of sensitivity analysis for pancreatic cancer

Excluding study	SIR and 95% CI	*P* value	Heterogeneity (%)	*P* value for heterogeneity
Villeneuve 2007	1.57 (1.25–1.97)	< 0.001	0.0	0.454
Collett 2010	1.56 (1.06–2.28)	0.024	5.3	0.377
Li 2012	1.55 (1.24–1.93)	< 0.001	0.0	0.426
Cheung 2012	1.53 (1.19–1.96)	0.001	6.6	0.369
Krynitz 2013	1.40 (1.09–1.79)	0.008	0.0	0.786
Piselli 2013	1.58 (1.26–1.98)	< 0.001	0.0	0.557

Supplementary Table 5: The details of sensitivity analysis for hepatocellular carcinoma

Excluding study	SIR and 95% CI	*P* value	Heterogeneity (%)	*P* value for heterogeneity
Hoshida 1997	2.54 (1.68–3.85)	< 0.001	75.4	< 0.001
Vajdic 2006	2.32 (1.47–3.66)	< 0.001	76.9	< 0.001
Vegso 2007	2.40 (1.58–3.64)	< 0.001	76.9	< 0.001
Villeneuve 2007	2.51 (1.64–3.85)	< 0.001	75.7	< 0.001
Collett 2010	2.41 (1.52–3.84)	< 0.001	75.1	< 0.001
Li 2012	2.24 (1.63–3.07)	< 0.001	35.2	0.147
Cheung 2012	2.38 (1.48–3.82)	< 0.001	75.4	< 0.001
Krynitz 2013	2.35 (1.46–3.78)	< 0.001	76.2	< 0.001
Piselli 2013	2.89 (2.07–4.03)	< 0.001	61.2	0.012

Supplementary Table 6: The details of sensitivity analysis for lung cancer

Excluding study	SIR and 95% CI	*P* value	Heterogeneity (%)	*P* value for heterogeneity
Vegso 2007	1.78 (1.36–2.32)	< 0.001	83.3	< 0.001
Villeneuve 2007	1.60 (1.18–2.17)	0.003	79.7	< 0.001
Collett 2010	1.75 (1.25–2.44)	0.001	81.3	< 0.001
Li 2012	1.51 (1.21–1.89)	< 0.001	74.0	0.002
Cheung 2012	1.68 (1.24–2.28)	0.001	85.2	< 0.001
Krynitz 2013	1.67 (1.21–2.32)	0.002	85.1	< 0.001
Piselli 2013	1.81 (1.36–2.41)	< 0.001	82.5	< 0.001

Supplementary Table 7: The details of sensitivity analysis for thyroid cancer

Excluding study	SIR and 95% CI	*P* value	Heterogeneity (%)	*P* value for heterogeneity
Hoshida 1997	4.87 (3.64–6.50)	< 0.001	53.8	0.021
Birkeland 2000	5.00 (3.73–6.70)	< 0.001	56.8	0.013
Kyllonen 2000	4.77 (3.52–6.47)	< 0.001	53.6	0.022
Vajdic 2006	4.75 (3.43–6.58)	< 0.001	52.5	0.026
Vegso 2007	5.03 (3.74–6.77)	< 0.001	57.2	0.013
Villeneuve 2007	5.01 (3.59–7.01)	< 0.001	56.9	0.013
Collett 2010	4.74 (3.43–6.54)	< 0.001	51.3	0.030
Li 2012	5.39 (4.09–7.10)	< 0.001	46.6	0.051
Cheung 2012	5.11 (3.73–7.02)	< 0.001	55.9	0.015
Krynitz 2013	5.15 (3.77–7.04)	< 0.001	55.3	0.017
Piselli 2013	5.69 (4.56–7.09)	< 0.001	22.5	0.236

Supplementary Table 8: The details of sensitivity analysis for urinary bladder cancer

Excluding study	SIR and 95% CI	*P* value	Heterogeneity (%)	*P* value for heterogeneity
Hoshida 1997	3.36 (1.36–8.27)	0.009	98.2	< 0.001
Birkeland 2000	3.46 (1.39–8.61)	0.008	98.2	< 0.001
Kyllonen 2000	3.55 (1.41–8.93)	0.007	98.2	< 0.001
Vajdic 2006	3.53 (1.32–9.47)	0.012	98.1	< 0.001
Vegso 2007	3.99 (1.63–9.77)	0.002	98.2	< 0.001
Villeneuve 2007	3.74 (1.46–9.56)	0.006	98.1	< 0.001
Collett 2010	3.66 (1.34–9.97)	0.011	98.0	< 0.001
Li 2012	2.63 (1.89–3.68)	< 0.001	78.3	< 0.001
Cheung 2012	3.22 (1.27–8.18)	0.014	98.2	< 0.001
Krynitz 2013	3.74 (1.43–9.77)	0.007	98.0	< 0.001
Piselli 2013	3.99 (1.61–9.89)	0.003	98.0	< 0.001

Supplementary Table 9: The details of sensitivity analysis for renal cell cancer

Excluding study	SIR and 95% CI	*P* value	Heterogeneity (%)	*P* value for heterogeneity
Hoshida 1997	8.83 (5.32–14.67)	< 0.001	96.6	< 0.001
Birkeland 2000	11.38 (6.61–19.60)	< 0.001	97.2	< 0.001
Kyllonen 2000	11.10 (6.35–19.42)	< 0.001	97.2	< 0.001
Vajdic 2006	11.21 (6.26–20.08)	< 0.001	97.1	< 0.001
Vegso 2007	11.00 (6.42–18.83)	< 0.001	97.2	< 0.001
Villeneuve 2007	11.21 (6.26–20.09)	< 0.001	97.1	< 0.001
Collett 2010	11.10 (5.99–20.58)	< 0.001	97.1	< 0.001
Li 2012	9.16 (6.54–12.83)	< 0.001	90.2	< 0.001
Cheung 2012	10.59 (6.02–18.63)	< 0.001	97.2	< 0.001
Krynitz 2013	11.42 (6.45–20.21)	< 0.001	97.0	< 0.001
Piselli 2013	11.70 (6.74–20.13)	< 0.001	97.0	< 0.001

Supplementary Table 10: The details of sensitivity analysis for non-melanoma skin cancer

Excluding study	SIR and 95% CI	*P* value	Heterogeneity (%)	*P* value for heterogeneity
Birkeland 2000	11.97 (5.92–24.23)	< 0.001	99.7	< 0.001
Kyllonen 2000	9.85 (4.86–19.99)	< 0.001	99.7	< 0.001
Vegso 2007	15.36 (7.75–30.44)	< 0.001	99.7	< 0.001
Collett 2010	11.07 (4.69–26.16)	< 0.001	98.7	< 0.001
Li 2012	15.04 (7.62–29.71)	< 0.001	99.7	< 0.001
Cheung 2012	13.23 (6.62–26.46)	< 0.001	99.7	< 0.001
Krynitz 2013	10.25 (6.53–16.08)	< 0.001	94.9	< 0.001

Supplementary Table 11: The details of sensitivity analysis for melanoma

Excluding study	SIR and 95% CI	*P* value	Heterogeneity (%)	*P* value for heterogeneity
Birkeland 2000	2.81 (0.97–8.19)	0.058	55.0	0.084
Vegso 2007	2.93 (1.26–6.81)	0.013	43.5	0.150
Li 2012	2.19 (0.85–5.67)	0.105	51.0	0.106
Cheung 2012	1.79 (1.03–3.12)	0.040	0.0	0.494
Piselli 2013	2.89 (0.86–9.72)	0.087	48.2	0.122

Supplementary Table 12: The details of sensitivity analysis for Hodgkin's lymphoma

Excluding study	SIR and 95% CI	*P* value	Heterogeneity (%)	*P* value for heterogeneity
Birkeland 2000	4.57 (2.68–7.79)	< 0.001	56.8	0.074
Vajdic 2006	5.14 (2.96–8.93)	< 0.001	47.7	0.125
Villeneuve 2007	5.38 (3.18–9.11)	< 0.001	38.2	0.183
Collett 2010	3.71 (2.32–5.92)	< 0.001	0.0	0.693
Piselli 2013	5.41 (3.46–8.46)	< 0.001	40.0	0.172

Supplementary Table 13: The details of sensitivity analysis for non-Hodgkin lymphoma

Excluding study	SIR and 95% CI	*P* value	Heterogeneity (%)	*P* value for heterogeneity
Birkeland 2000	11.10 (8.89–13.86)	< 0.001	82.2	< 0.001
Vajdic 2006	10.74 (7.97–14.48)	< 0.001	81.9	< 0.001
Vegso 2007	10.83 (8.75–13.42)	< 0.001	82.1	< 0.001
Villeneuve 2007	11.35 (8.88–14.51)	< 0.001	75.4	0.003
Collett 2010	9.99 (7.49–13.32)	< 0.001	75.9	0.002
Cheung 2012	9.77 (7.74–12.34)	< 0.001	79.2	0.001

Supplementary Table 14: The details of sensitivity analysis for lip cancer

Excluding study	SIR and 95% CI	*P* value	Heterogeneity (%)	*P* value for heterogeneity
Birkeland 2000	38.06 (28.42–50.98)	< 0.001	83.5	< 0.001
Kyllonen 2000	30.55 (17.70–52.72)	< 0.001	97.0	< 0.001
Vajdic 2006	26.67 (14.29–49.77)	< 0.001	96.0	< 0.001
Villeneuve 2007	28.89 (16.03–52.05)	< 0.001	97.0	< 0.001
Collett 2010	25.46 (14.63–44.31)	< 0.001	96.5	< 0.001
Krynitz 2013	26.97 (14.97–48.60)	< 0.001	96.9	< 0.001
Piselli 2013	33.38 (19.82–56.21)	< 0.001	96.9	< 0.001

Supplementary Table 15: The details of sensitivity analysis for breast cancer

Excluding study	SIR and 95% CI	*P* value	Heterogeneity (%)	*P* value for heterogeneity
Hoshida 1997	1.11 (1.00–1.24)	0.049	0.0	0.459
Birkeland 2000	1.11 (0.99–1.23)	0.067	0.0	0.510
Kyllonen 2000	1.11 (1.00–1.24)	0.055	0.0	0.452
Vajdic 2006	1.13 (1.01–1.27)	0.038	0.0	0.484
Vegso 2007	1.12 (1.01–1.25)	0.039	0.0	0.488
Villeneuve 2007	1.08 (0.96–1.22)	0.185	0.0	0.599
Collett 2010	1.16 (1.03–1.32)	0.019	0.0	0.599
Li 2012	1.11 (1.00–1.24)	0.052	0.0	0.447
Cheung 2012	1.09 (0.98–1.22)	0.106	0.0	0.698
Krynitz 2013	1.10 (0.98–1.23)	0.121	0.0	0.483
Piselli 2013	1.14 (1.02–1.27)	0.021	0.0	0.684

Supplementary Table 16: The details of sensitivity analysis for ovarian cancer

Excluding study	SIR and 95% CI	*P* value	Heterogeneity (%)	*P* value for heterogeneity
Vajdic 2006	1.65 (1.26–2.17)	< 0.001	0.0	0.447
Villeneuve 2007	1.62 (1.20–2.17)	0.001	8.3	0.359
Collett 2010	1.75 (1.25–2.47)	0.001	0.0	0.452
Cheung 2012	1.49 (1.13–1.95)	0.004	0.0	0.825
Krynitz 2013	1.50 (1.11–2.03)	0.008	0.0	0.434
Piselli 2013	1.62 (1.24–2.12)	< 0.001	2.3	0.394

Supplementary Table 17: The details of sensitivity analysis for uterus cancer

Excluding study	SIR and 95% CI	*P* value	Heterogeneity (%)	*P* value for heterogeneity
Hoshida 1997	1.12 (0.83–1.51)	0.449	0.0	0.921
Vajdic 2006	1.17 (0.86–1.58)	0.314	0.0	0.427
Villeneuve 2007	1.28 (0.93–1.76)	0.129	6.4	0.379
Collett 2010	1.34 (0.95–1.89)	0.100	0.4	0.421
Li 2012	1.25 (0.91–1.73)	0.167	12.1	0.337
Cheung 2012	1.23 (0.89–1.70)	0.209	11.3	0.343
Krynitz 2013	1.31 (0.93–1.84)	0.120	5.0	0.389
Piselli 2013	1.24 (0.89–1.74)	0.207	12.2	0.337

Supplementary Table 18: The details of sensitivity analysis for prostate cancer

Excluding study	SIR and 95% CI	*P* value	Heterogeneity (%)	*P* value for heterogeneity
Vajdic 2006	1.15 (0.96–1.38)	0.142	38.5	0.136
Vegso 2007	1.13 (0.96–1.33)	0.152	39.1	0.131
Villeneuve 2007	1.15 (0.96–1.36)	0.122	37.2	0.145
Collett 2010	1.11 (0.90–1.38)	0.320	44.6	0.094
Li 2012	1.10 (0.93–1.30)	0.256	39.6	0.127
Cheung 2012	1.12 (0.95–1.33)	0.182	42.9	0.105
Krynitz 2013	1.11 (0.89–1.39)	0.339	44.5	0.094
Piselli 2013	1.05 (0.93–1.19)	0.397	0.0	0.743

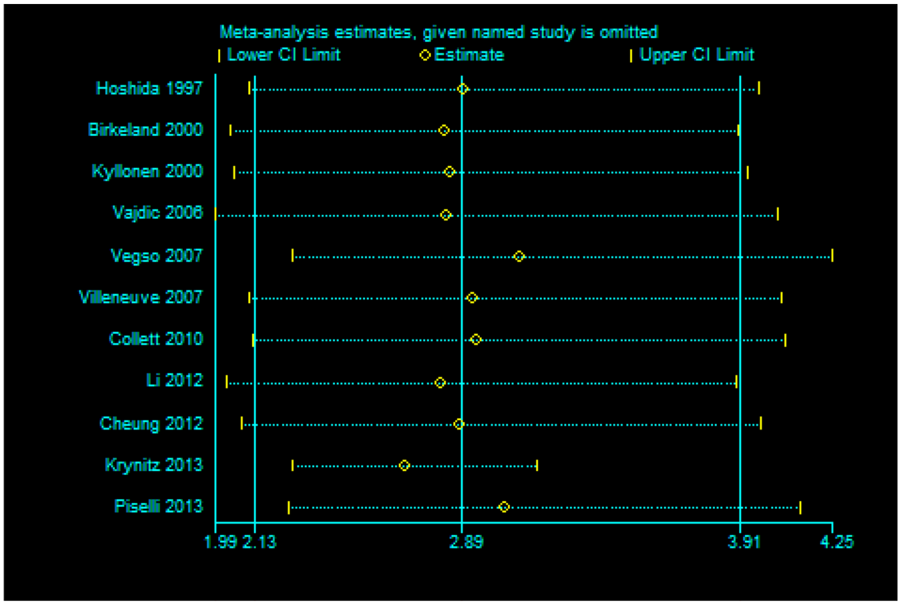

Supplementary Figure 1: Sensitivity analysis for all cancers.

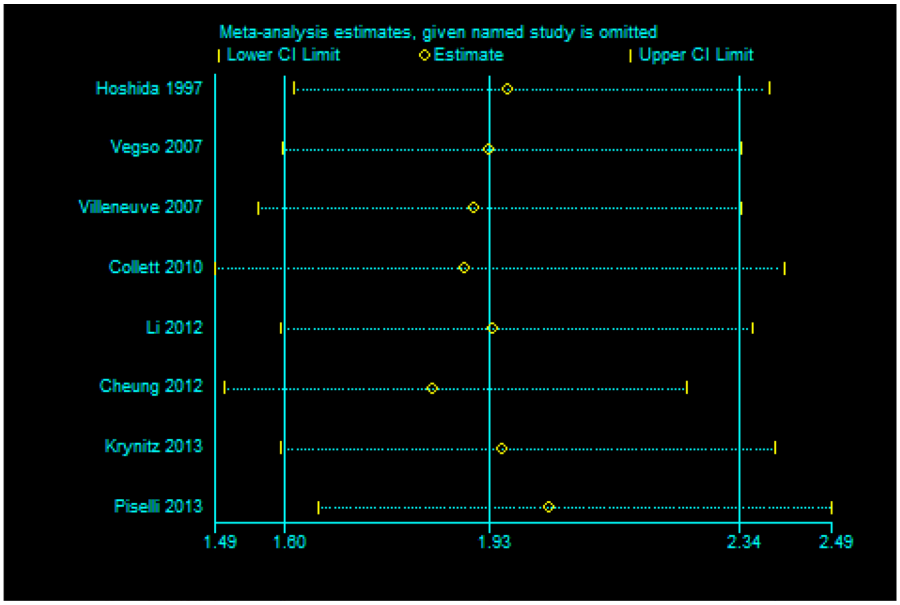

Supplementary Figure 2: Sensitivity analysis for gastric cancer.

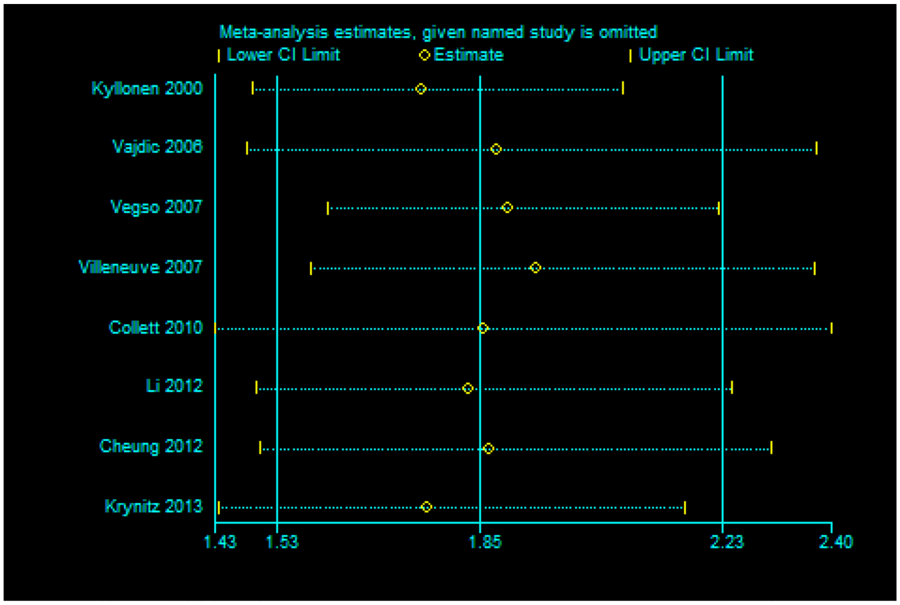

Supplementary Figure 3: Sensitivity analysis for colon cancer.

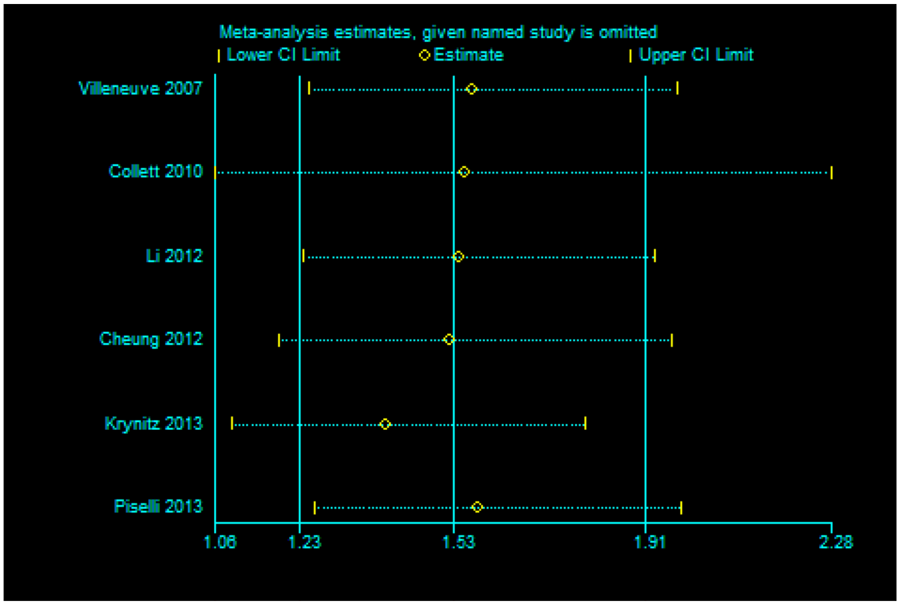

Supplementary Figure 4: Sensitivity analysis for pancreatic cancer.

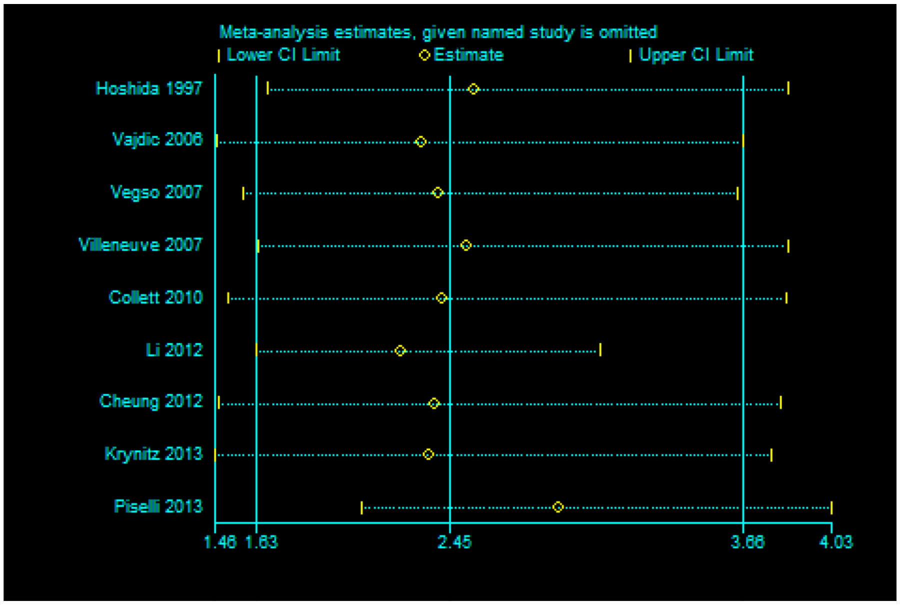

Supplementary Figure 5: Sensitivity analysis for hepatocellular carcinoma.

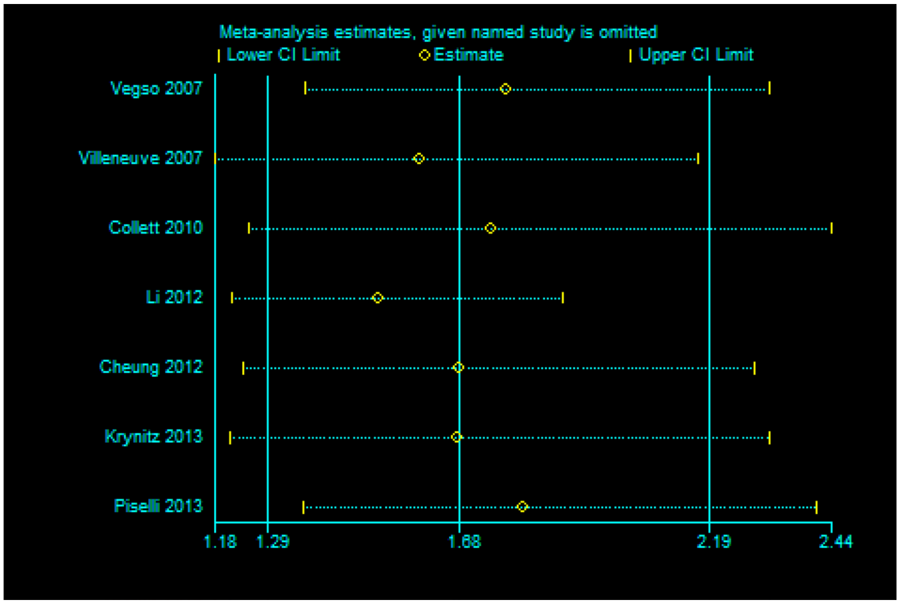

Supplementary Figure 6: Sensitivity analysis for lung cancer.

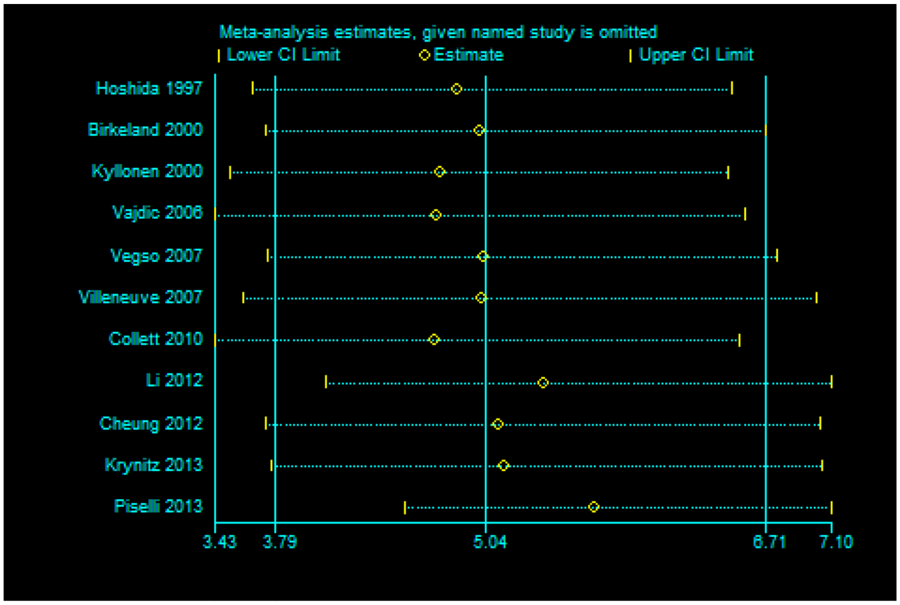

Supplementary Figure 7: Sensitivity analysis for thyroid cancer.

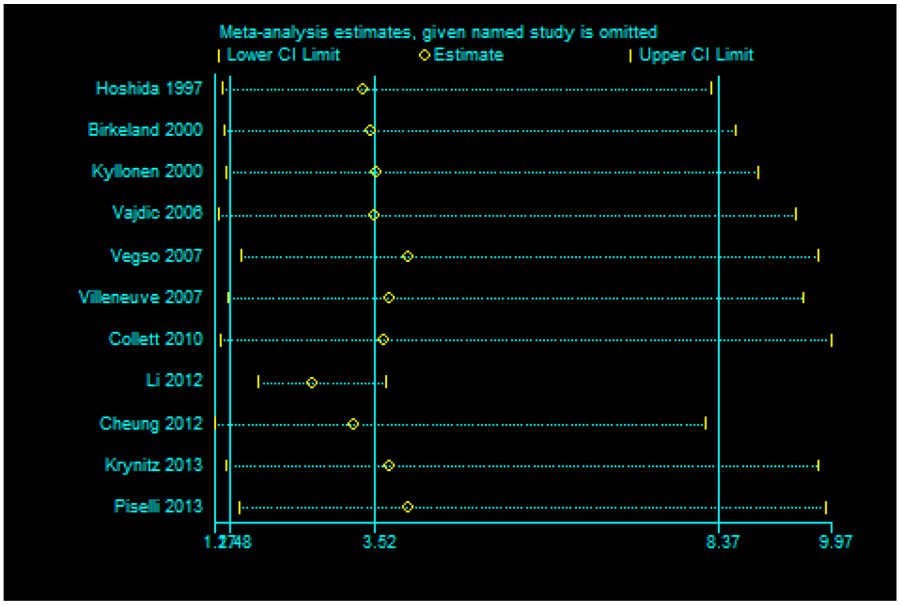

Supplementary Figure 8: Sensitivity analysis for urinary bladder cancer.

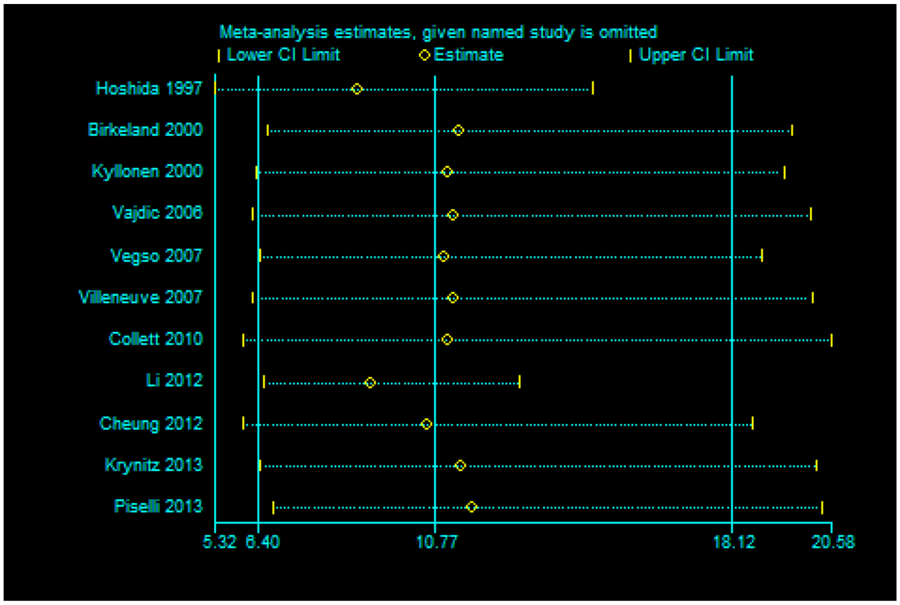

Supplementary Figure 9: Sensitivity analysis for renal cell cancer.

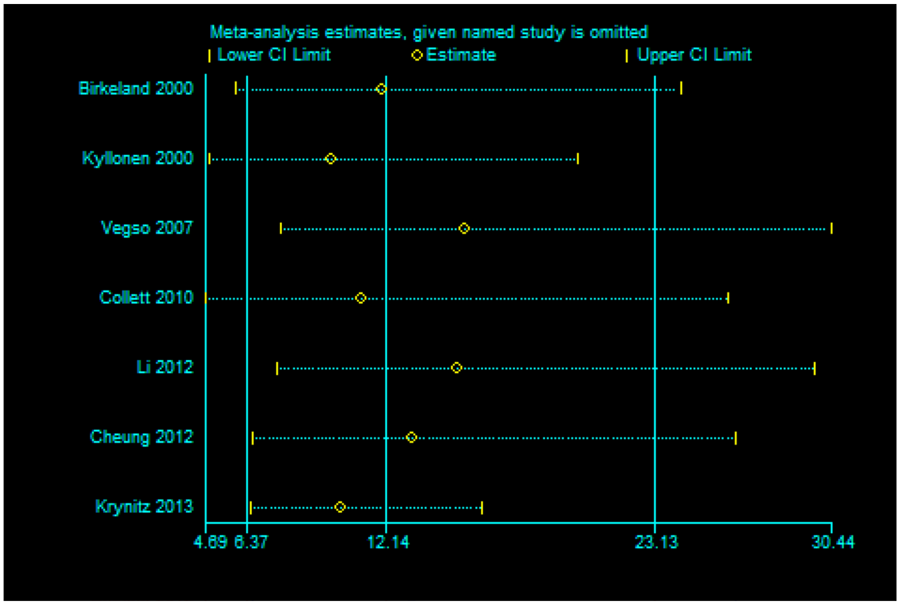

Supplementary Figure 10: Sensitivity analysis for non-melanoma skin cancer.

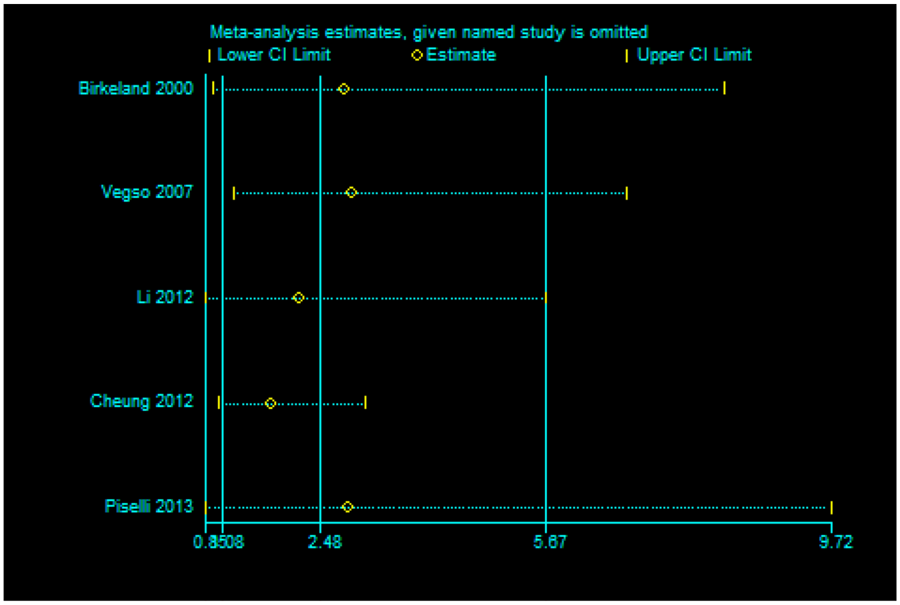

Supplementary Figure 11: Sensitivity analysis for melanoma.

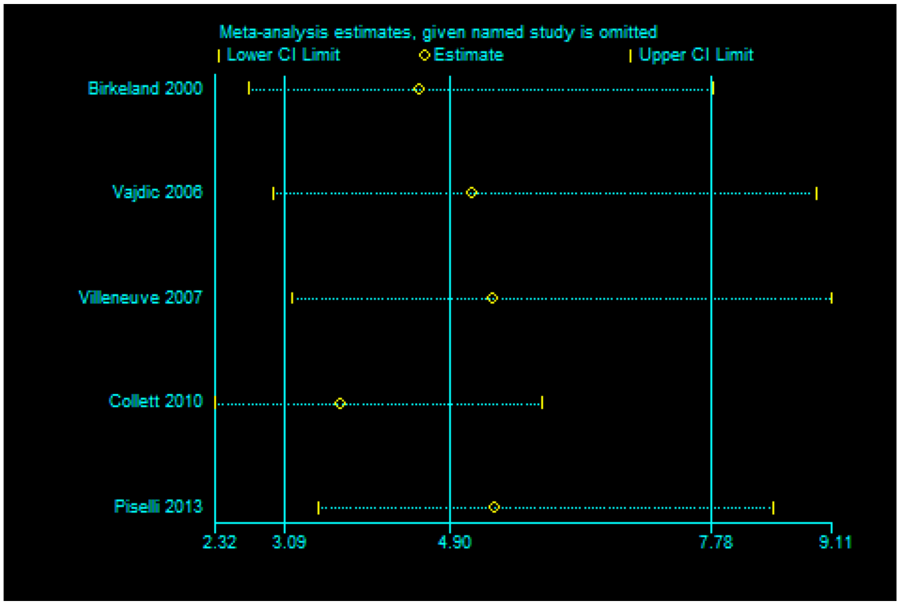

Supplementary Figure 12: Sensitivity analysis for Hodgkin's lymphoma.

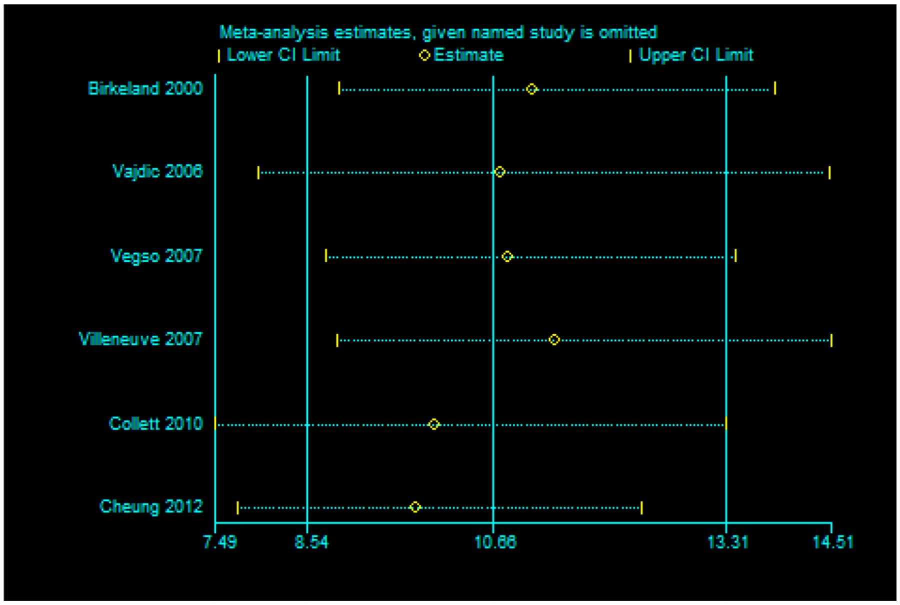

Supplementary Figure 13: Sensitivity analysis for non-Hodgkin lymphoma.

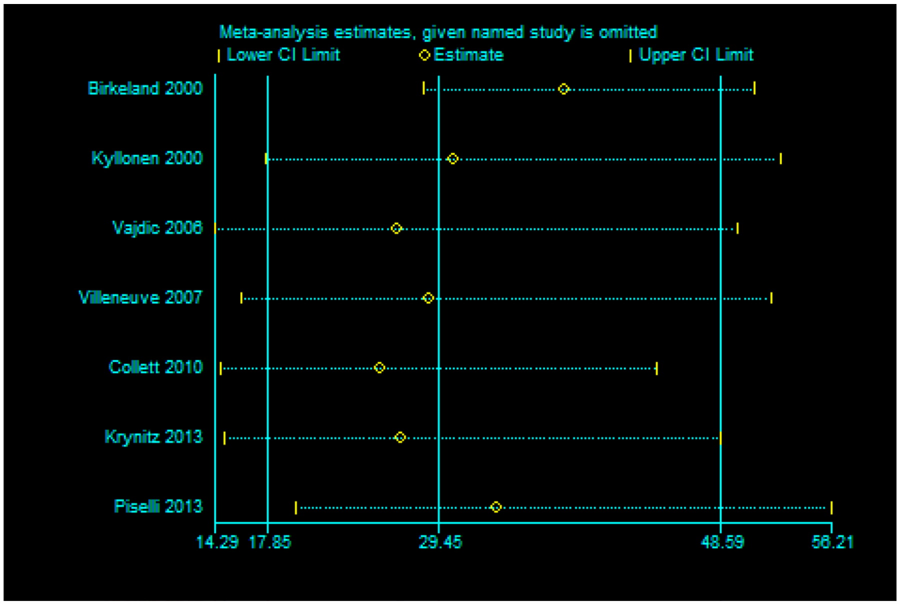

Supplementary Figure 14: Sensitivity analysis for lip cancer.

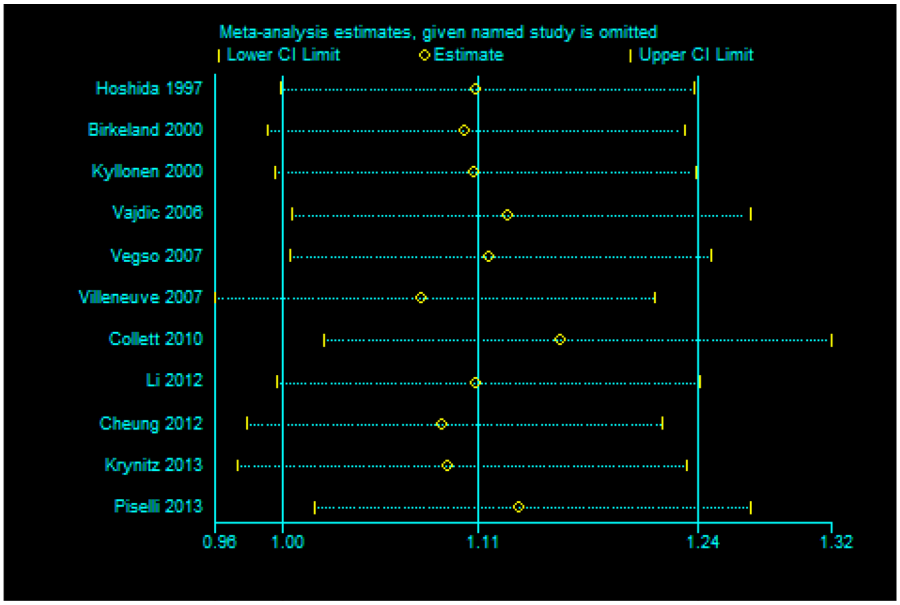

Supplementary Figure 15: Sensitivity analysis for breast cancer.

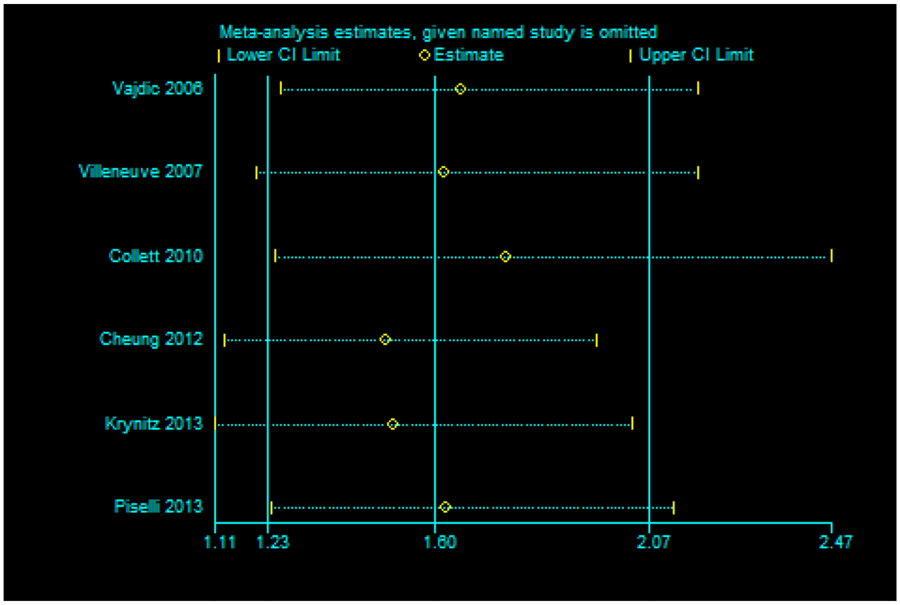

Supplementary Figure 16: Sensitivity analysis for ovarian cancer.

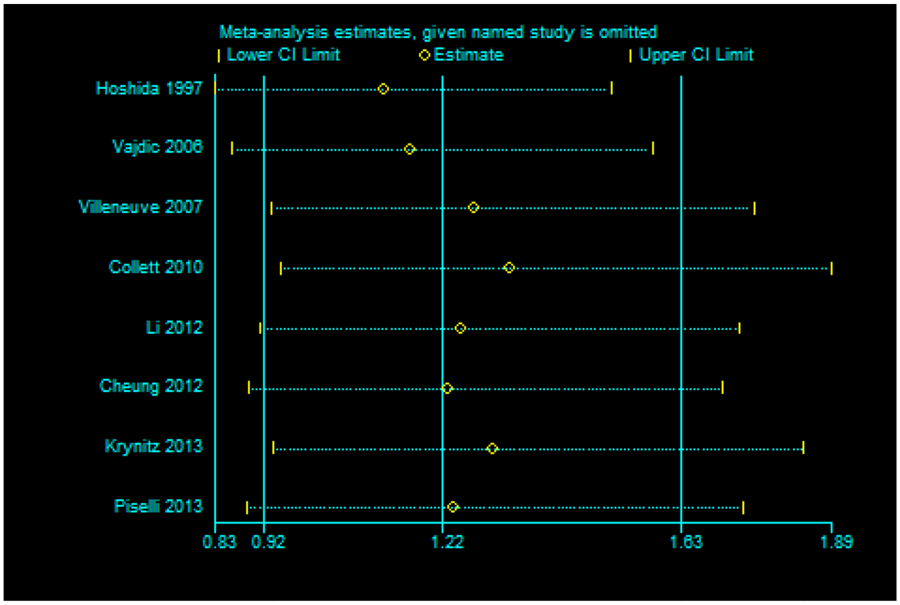

Supplementary Figure 17: Sensitivity analysis for uterus cancer.

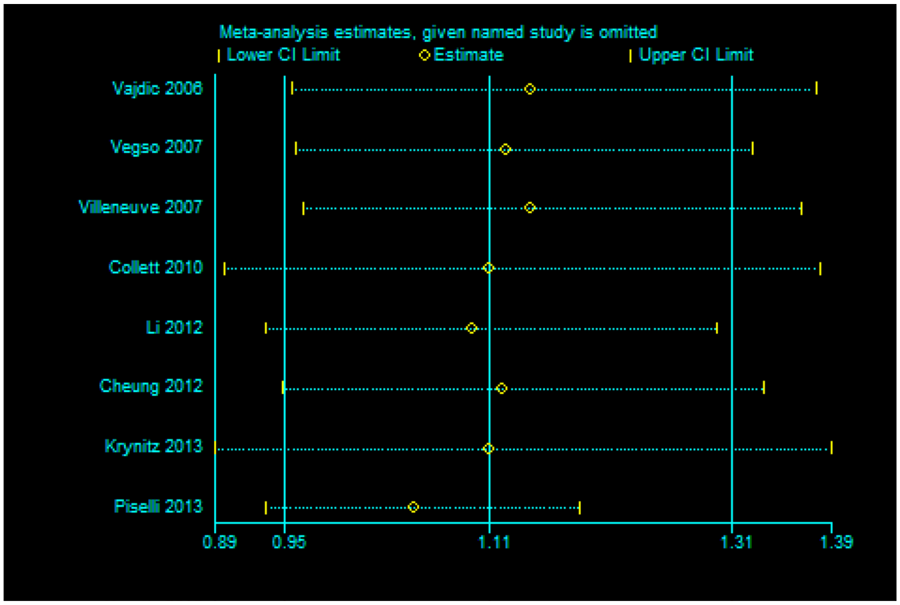

Supplementary Figure 18: Sensitivity analysis for prostate cancer.

Supplementary 3

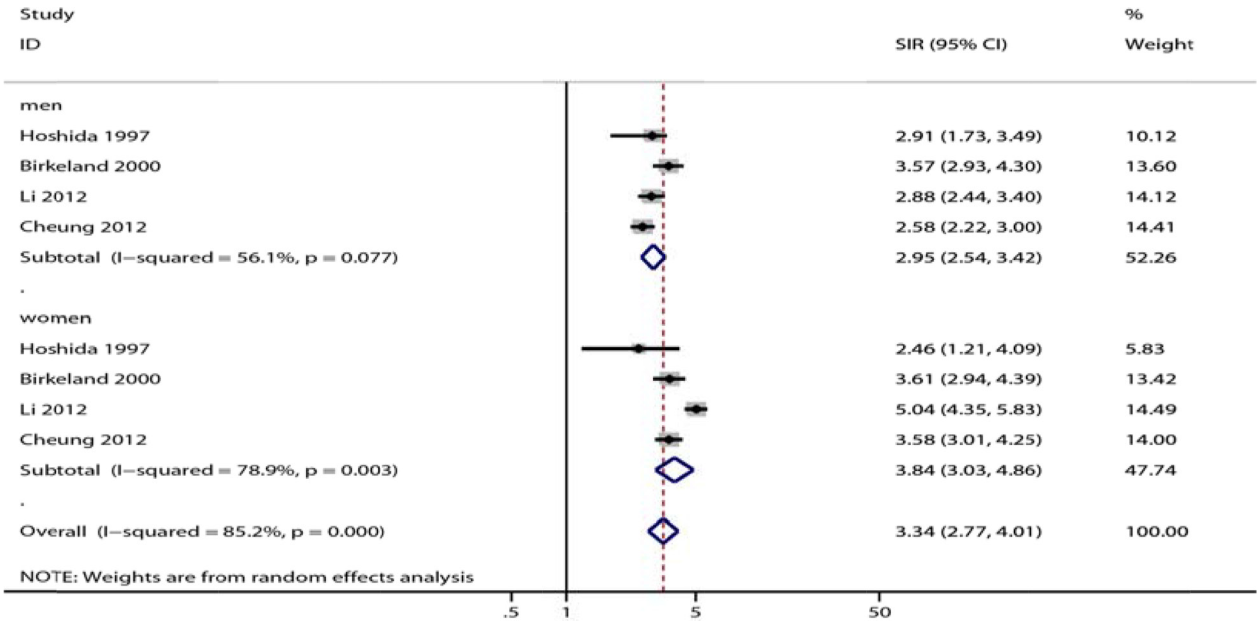

Supplementary Figure 1: All cancer risk in renal transplant recipients in men and women separately.

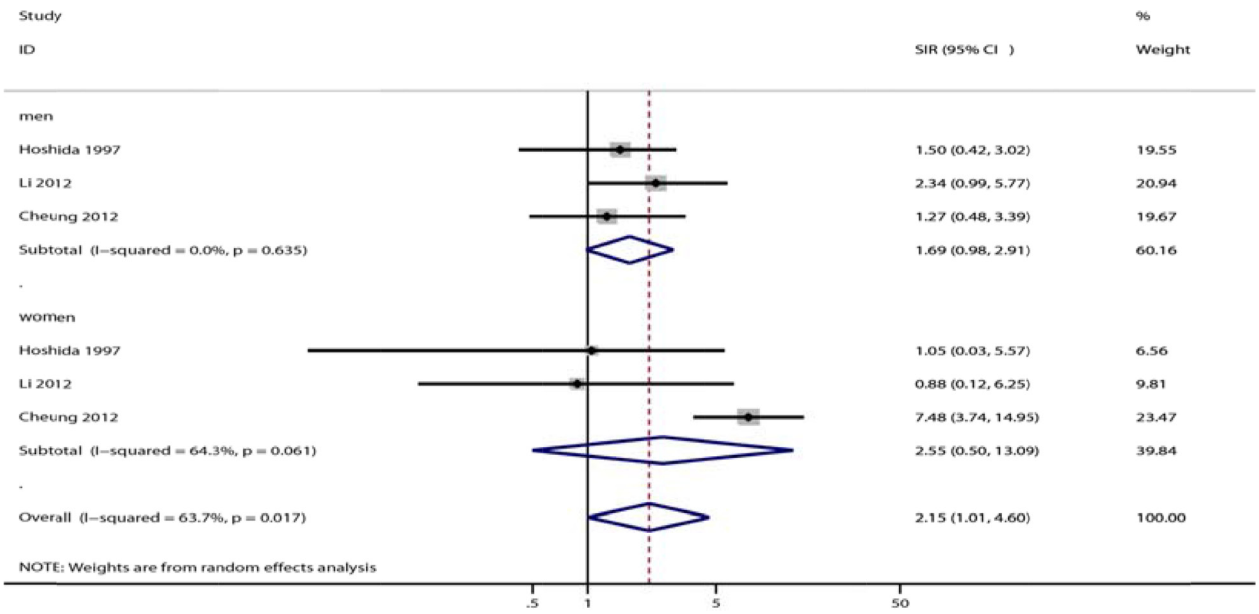

Supplementary Figure 2: Gastric cancer risk in renal transplant recipients in men and women separately.

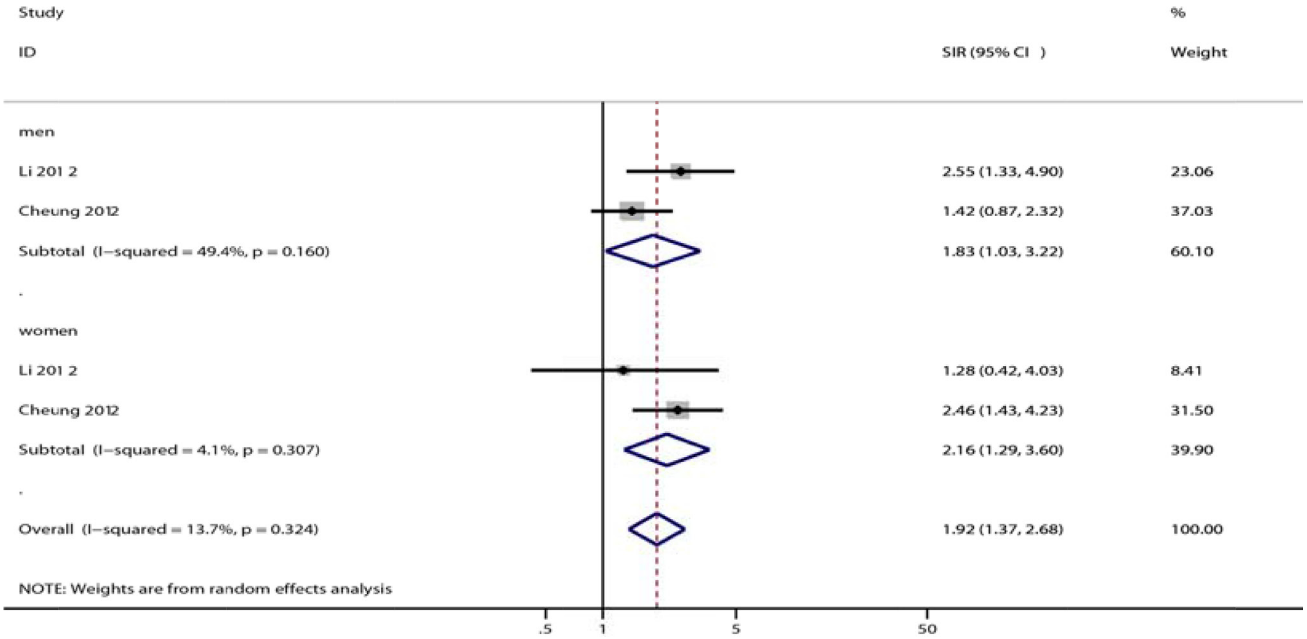

Supplementary Figure 3: Colon cancer risk in renal transplant recipients in men and women separately.

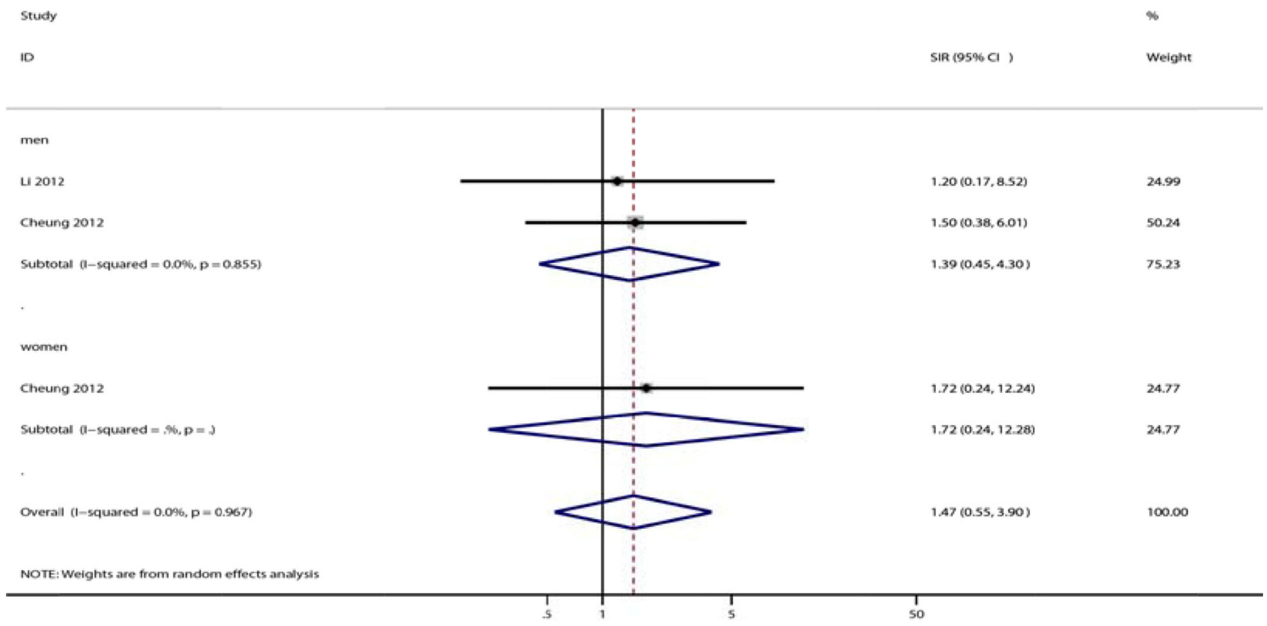

Supplementary Figure 4: Pancreatic cancer risk in renal transplant recipients in men and women separately.

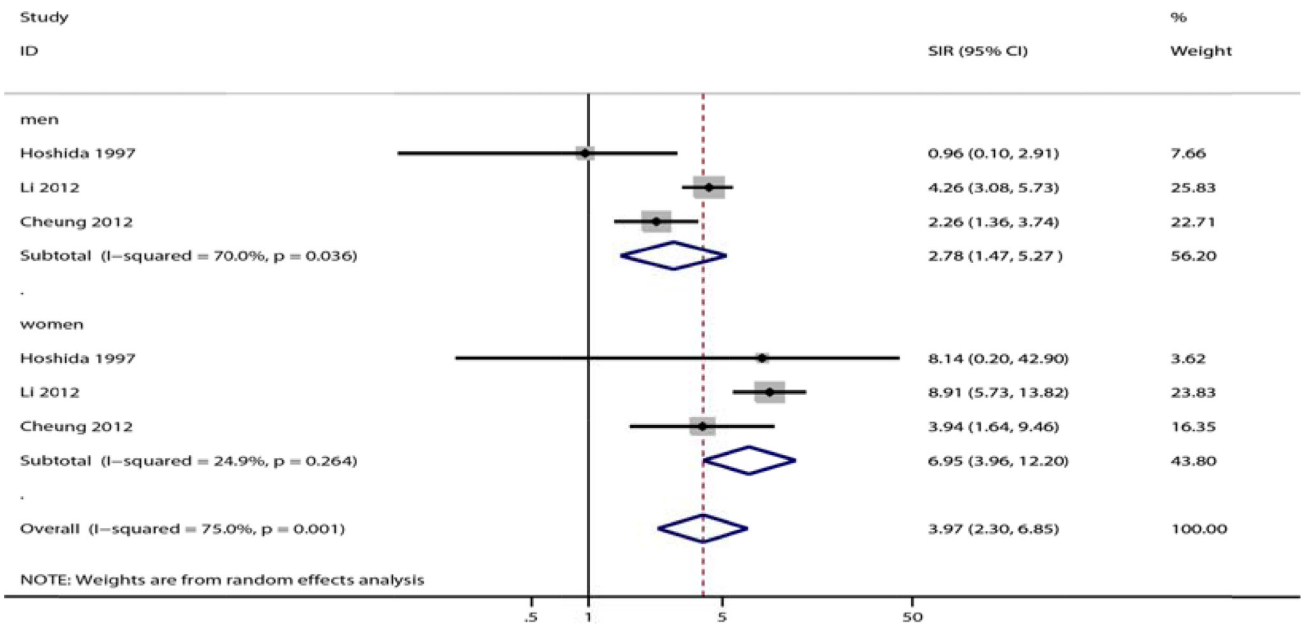

Supplementary Figure 5: Hepatocellular carcinoma risk in renal transplant recipients in men and women separately.

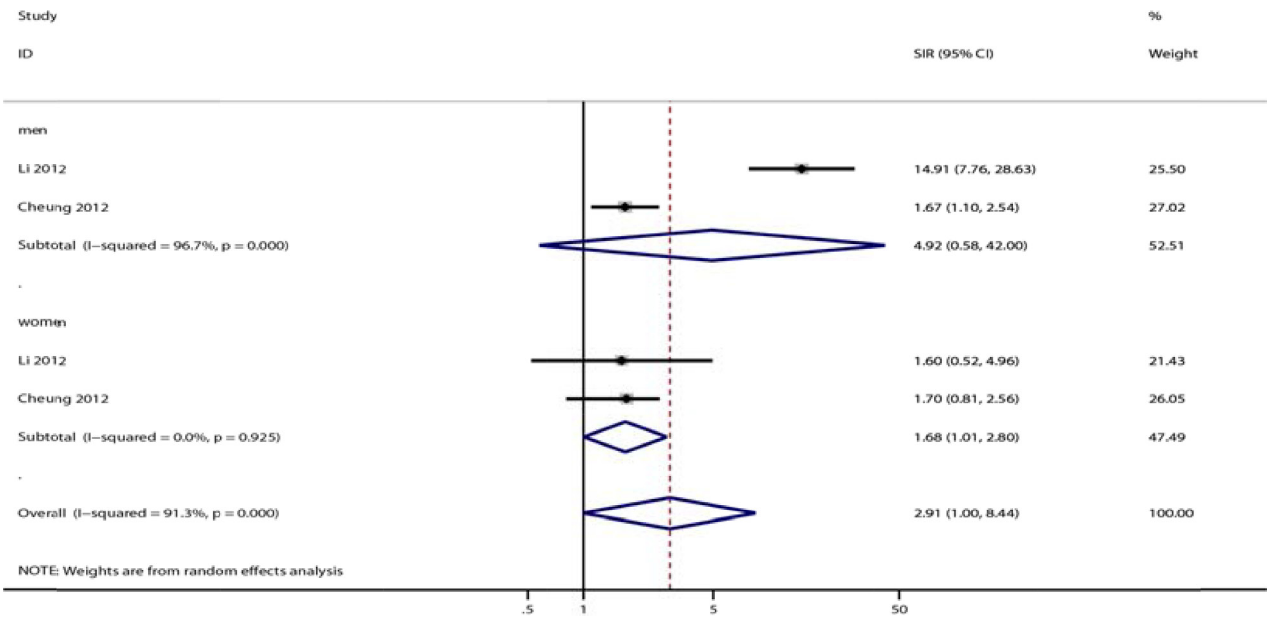

Supplementary Figure 6: Lung cancer risk in renal transplant recipients in men and women separately.

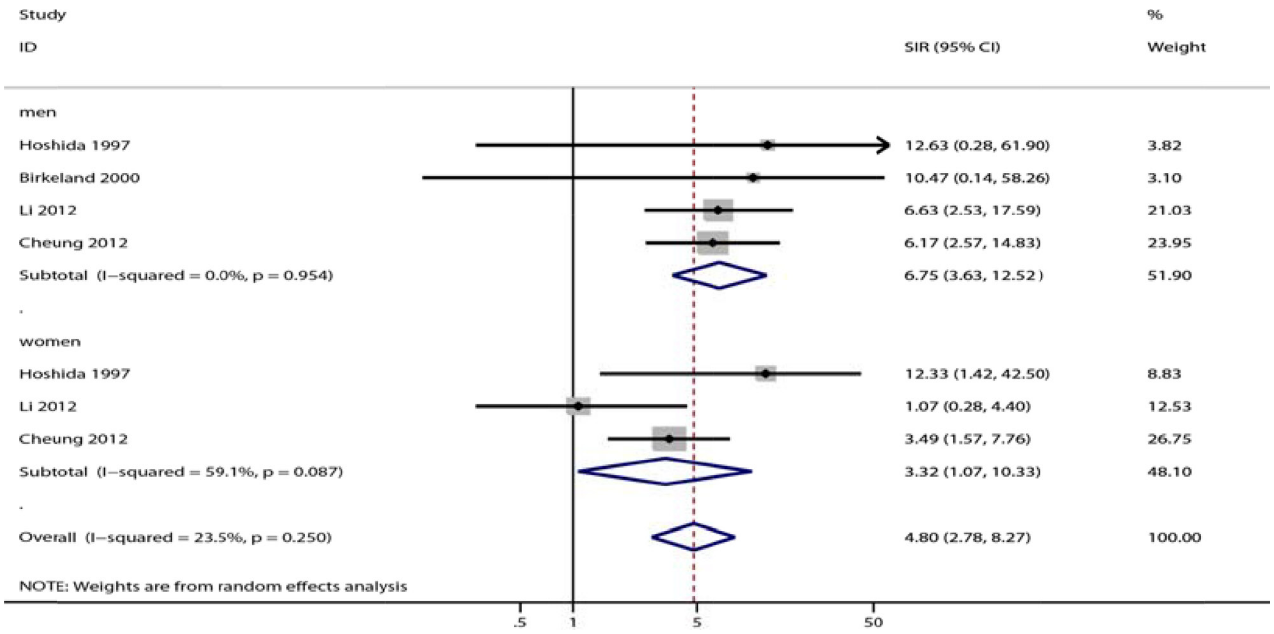

Supplementary Figure 7: Thyroid cancer risk in renal transplant recipients in men and women separately.

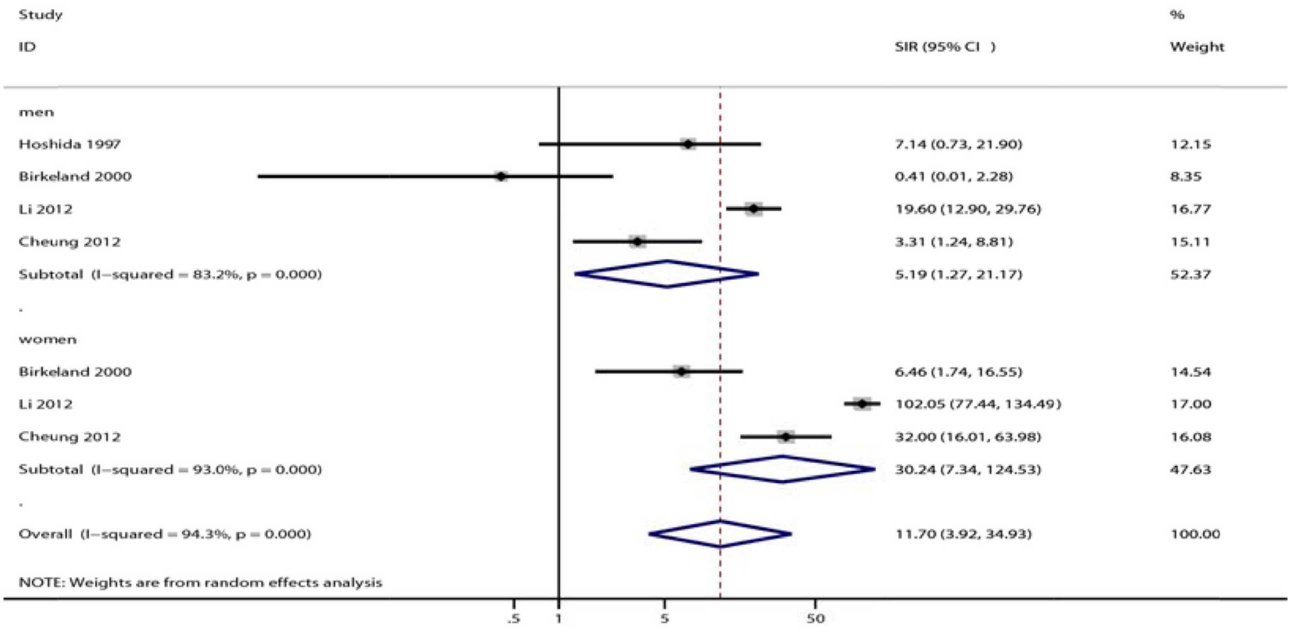

Supplementary Figure 8: Urinary bladder cancer risk in renal transplant recipients in men and women separately.

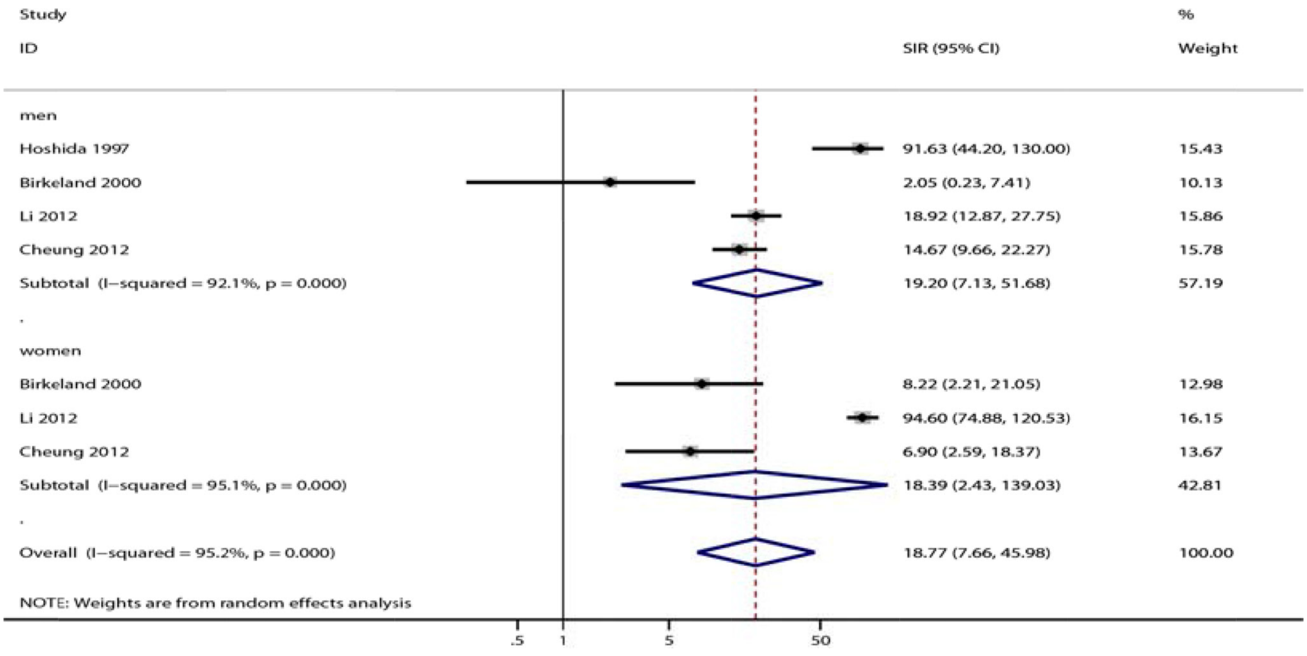

Supplementary Figure 9: Renal cell cancer risk in renal transplant recipients in men and women separately.

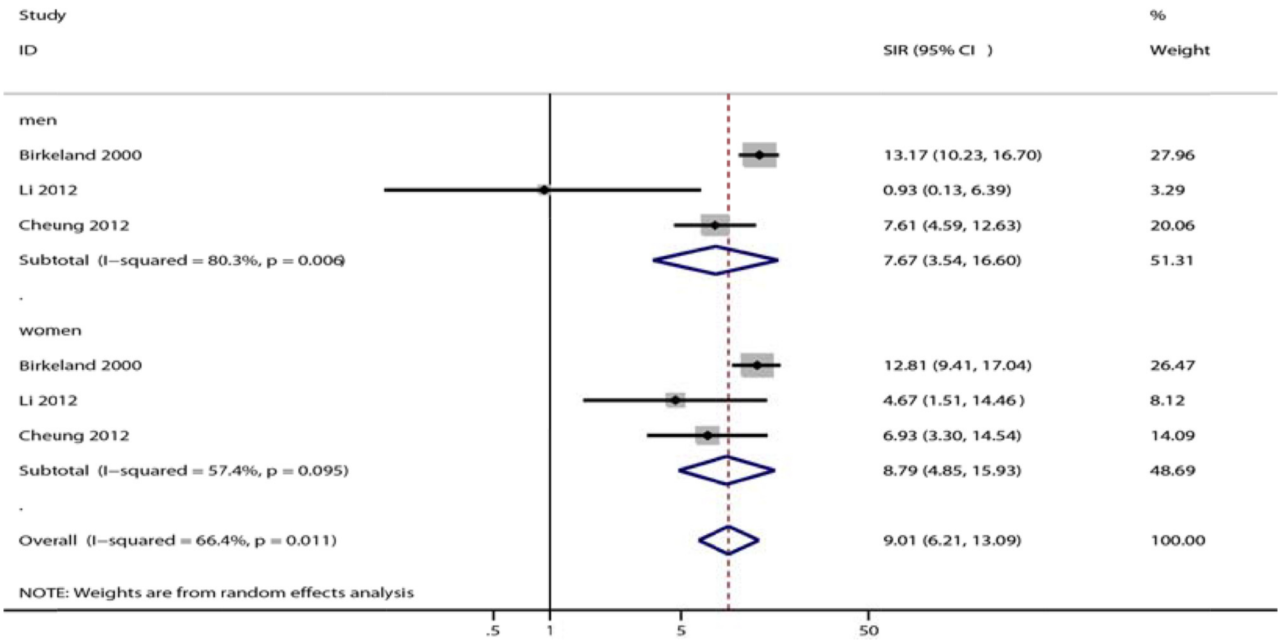

Supplementary Figure 10: Non-melanoma skin cancer risk in renal transplant recipients in men and women separately.

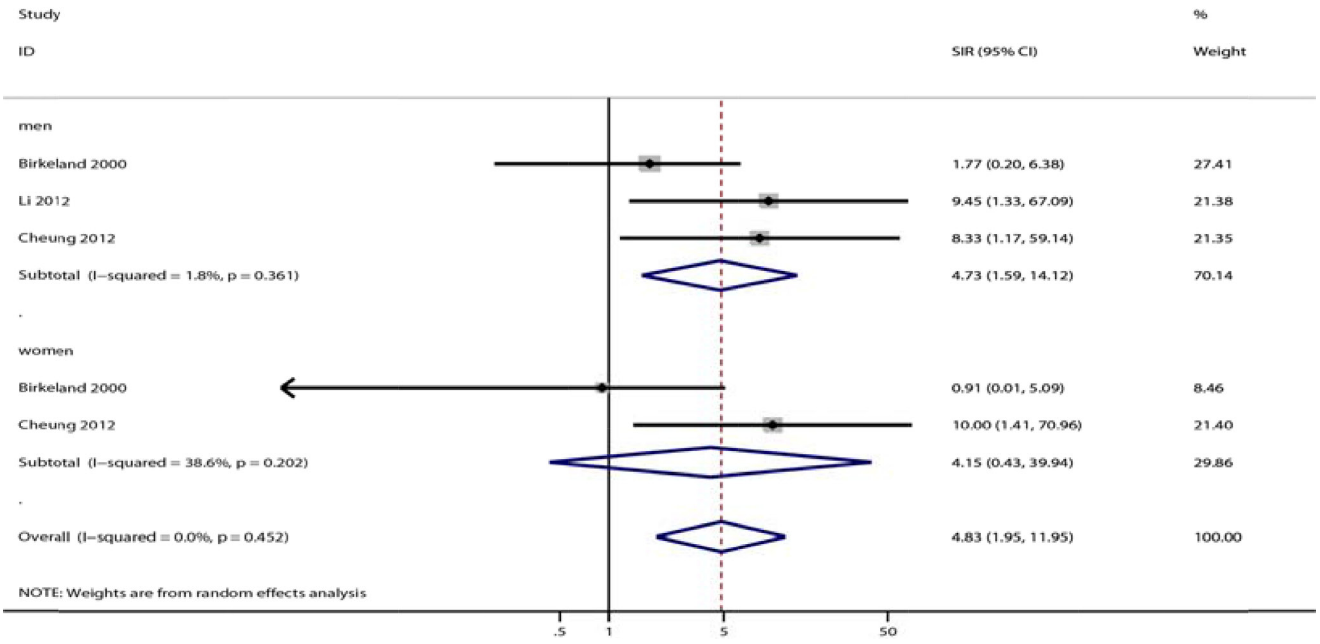

Supplementary Figure 11: Melanoma risk in renal transplant recipients in men and women separately.

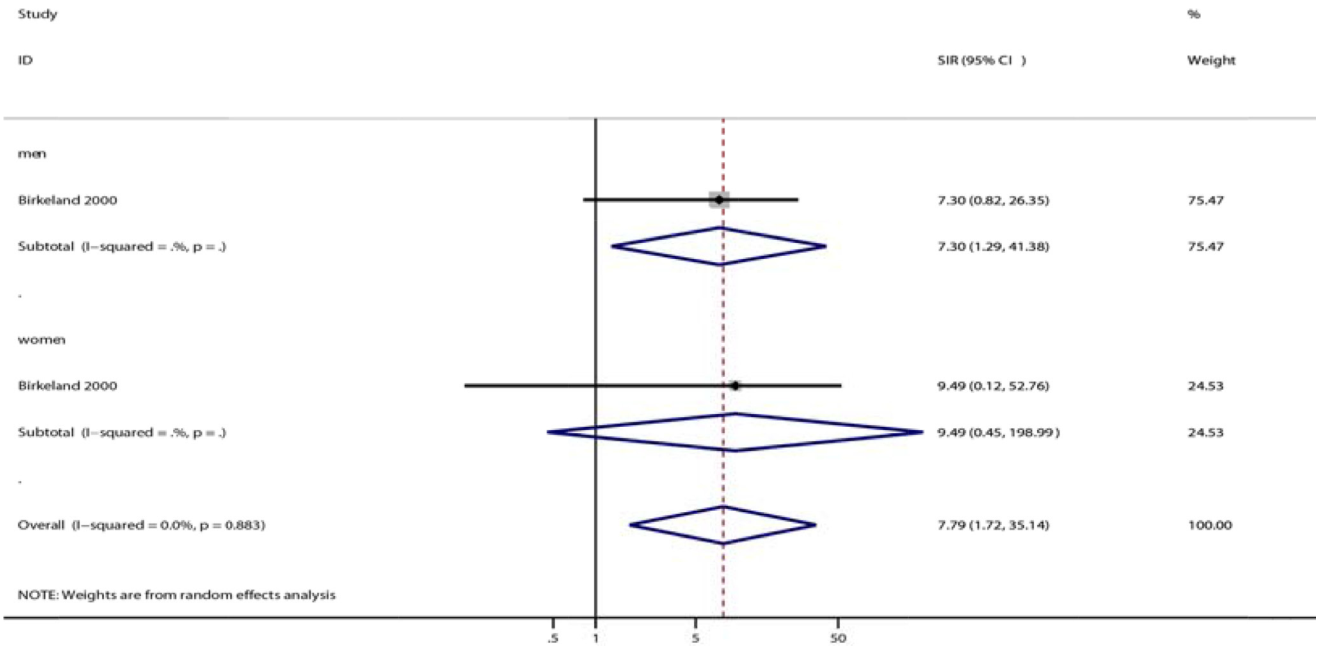

Supplementary Figure 12: Hodgkin's lymphoma risk in renal transplant recipients in men and women separately.

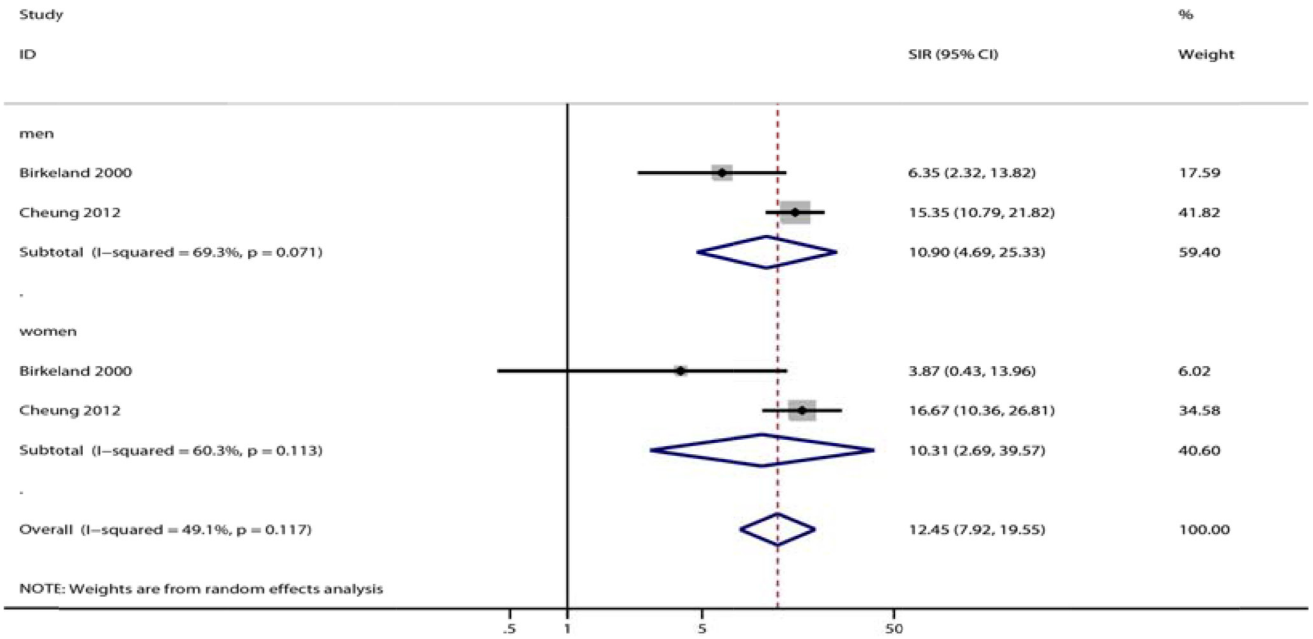

Supplementary Figure 13: Non-Hodgkin lymphoma risk in renal transplant recipients in men and women separately.

Supplementary 4

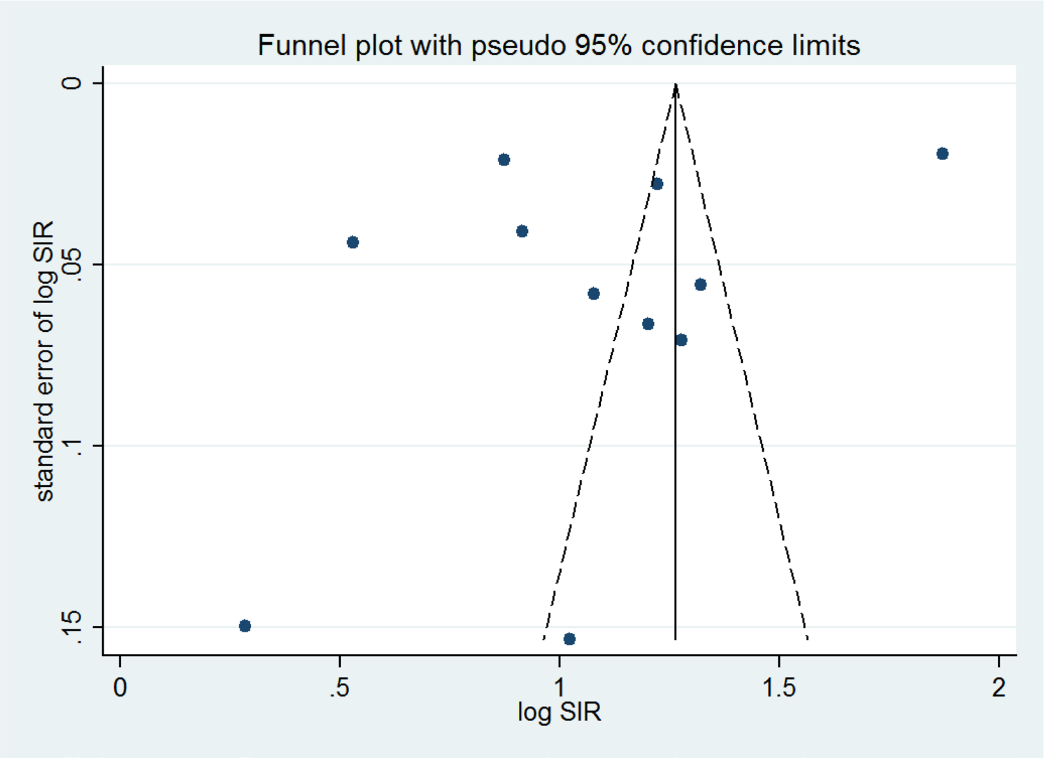

Supplementary Figure 1: NFunnel plot for all cancer (*P* value for Egger: 0.300; P value for Begg: 0.876).

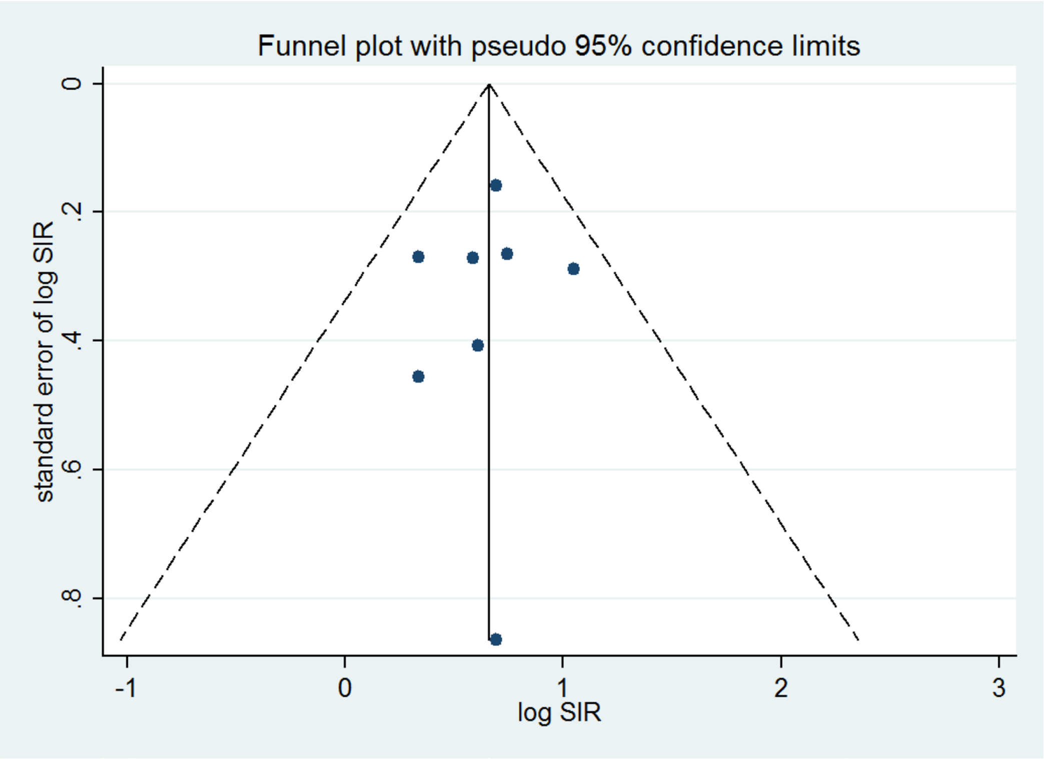

Supplementary Figure 2: Funnel plot for gastric cancer (*P* value for Egger: 0.690; *P* value for Begg: 0.902).

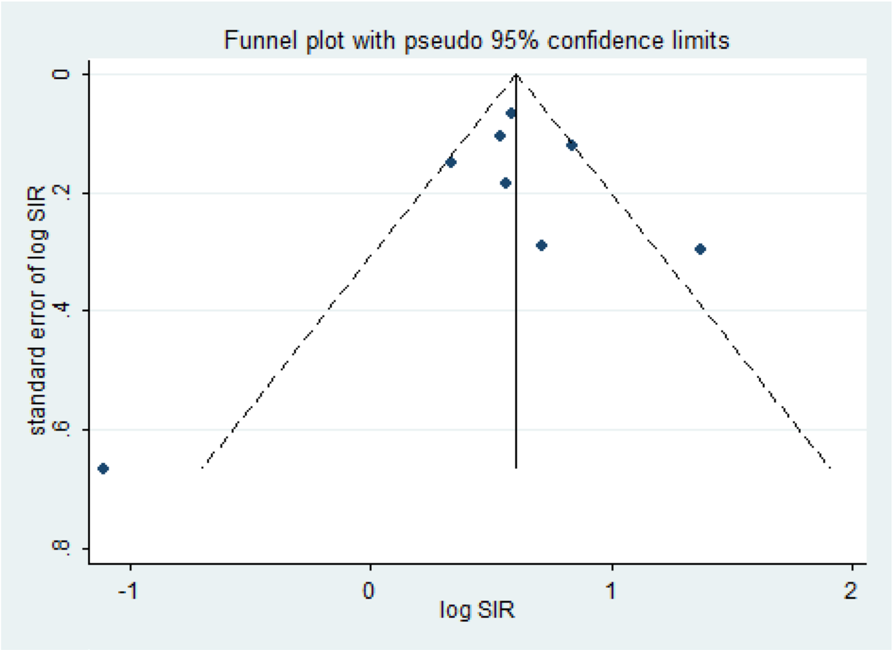

Supplementary Figure 3: Funnel plot for colon cancer (*P* value for Egger: 0.853; *P* value for Begg: 0.902).

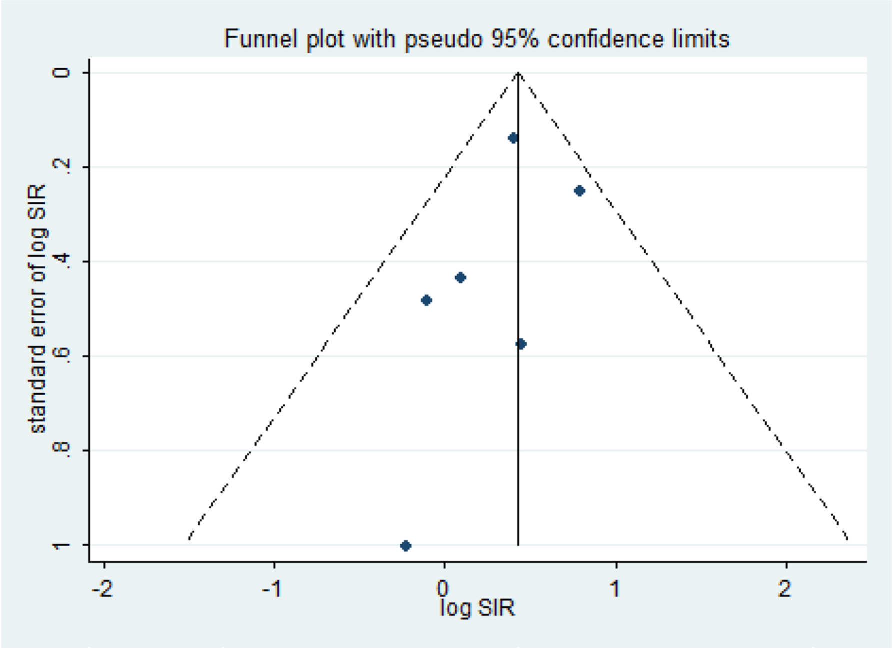

Supplementary Figure 4: Funnel plot for pancreatic cancer (*P* value for Egger: 0.401; *P* value for Begg: 0.707).

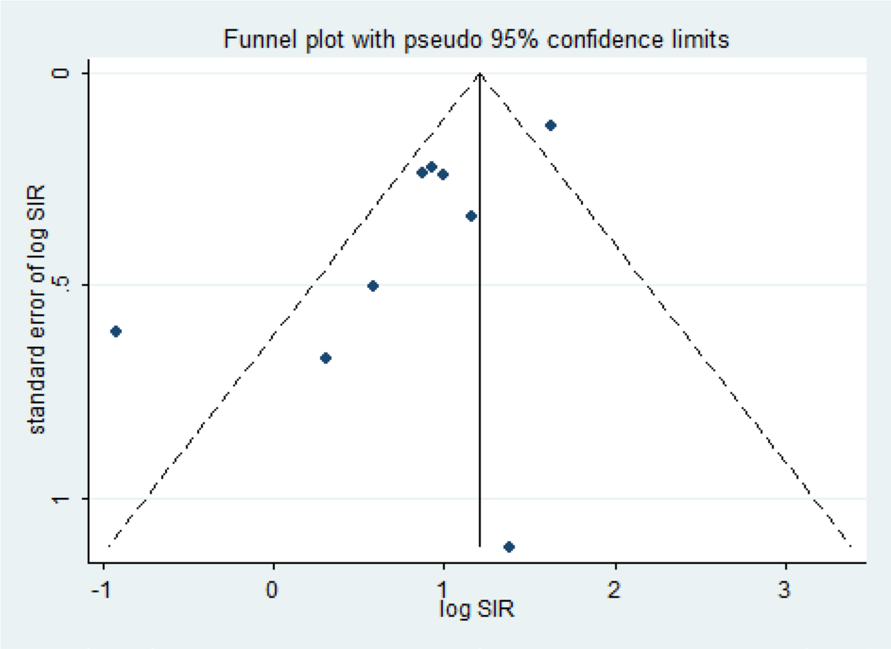

Supplementary Figure 5: Funnel plot for hepatocellular carcinoma (*P* value for Egger: 0.027; *P* value for Begg: 0.917).

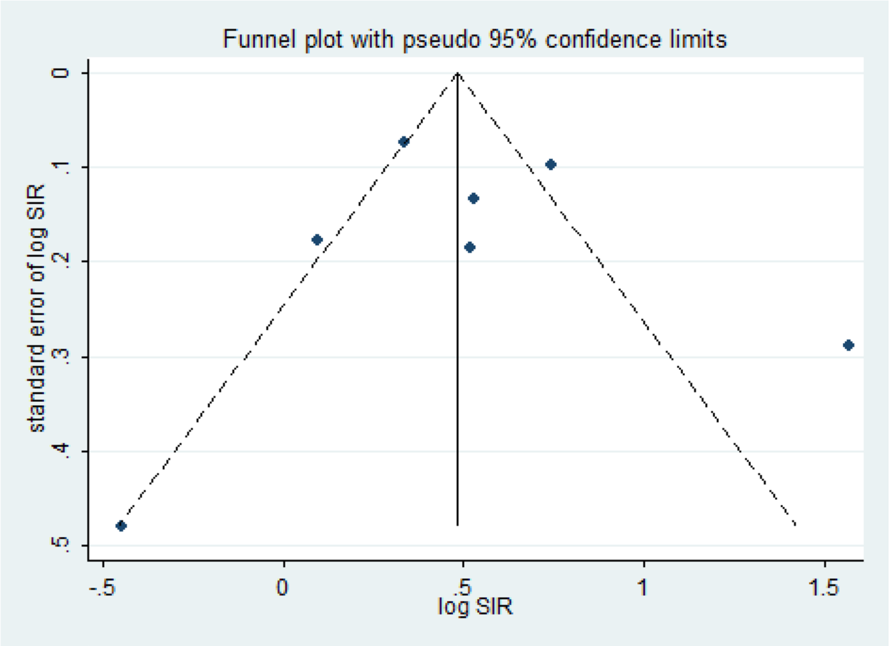

Supplementary Figure 6: Funnel plot for lung cancer (*P* value for Egger: 0.806; *P* value for Begg: 0.764).

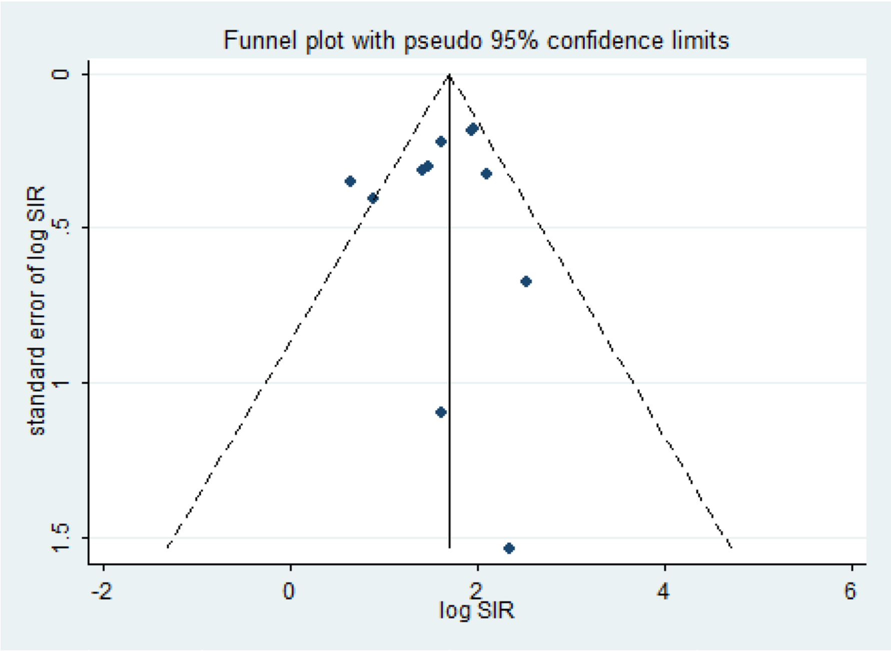

Supplementary Figure 7: Funnel plot for thyroid cancer (*P* value for Egger: 0.528; *P* value for Begg: 0.276).

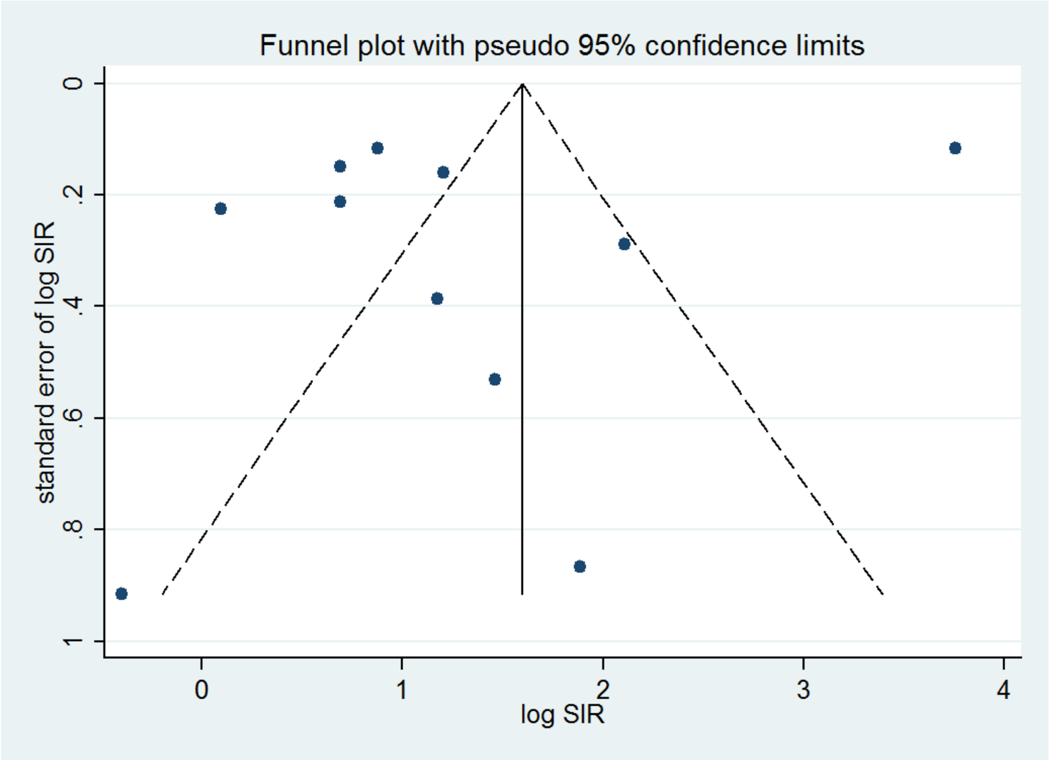

Supplementary Figure 8: Funnel plot for urinary bladder cancer (*P* value for Egger: 0.494; *P* value for Begg: 0.276).

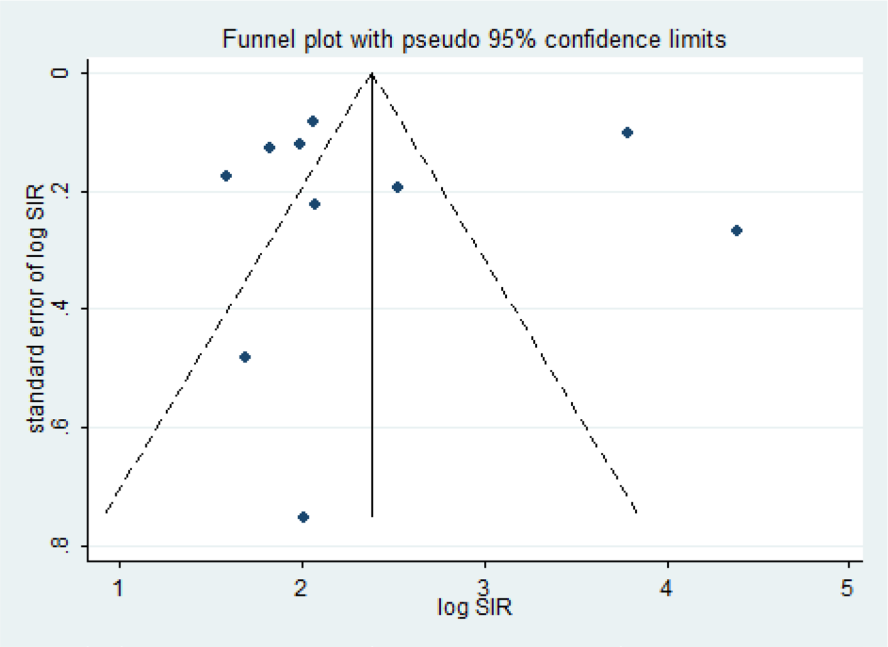

Supplementary Figure 9: Funnel plot for renal cell cancer (*P* value for Egger: 0.971; *P* value for Begg: 0.350).

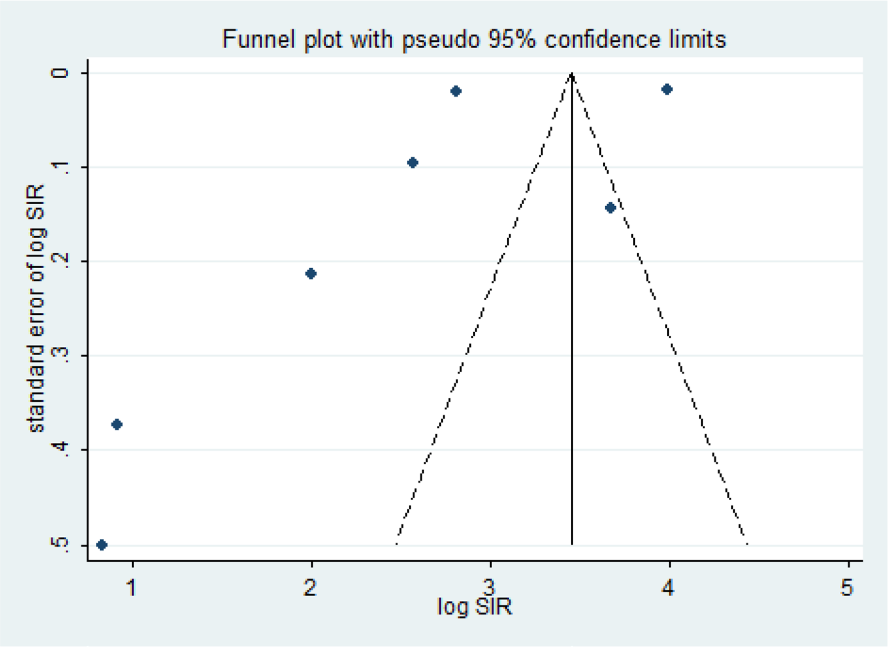

Supplementary Figure 10: Funnel plot for non-melanoma skin cancer (*P* value for Egger: 0.490; *P* value for Begg: 0.764).

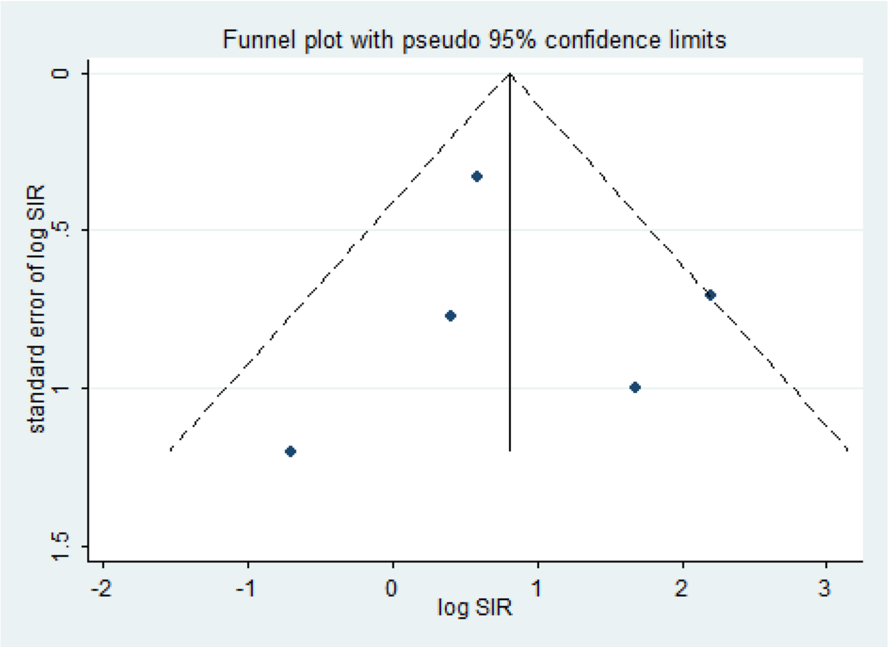

Supplementary Figure 11: Funnel plot for melanoma (*P* value for Egger: 0.791; *P* value for Begg: 0.806).

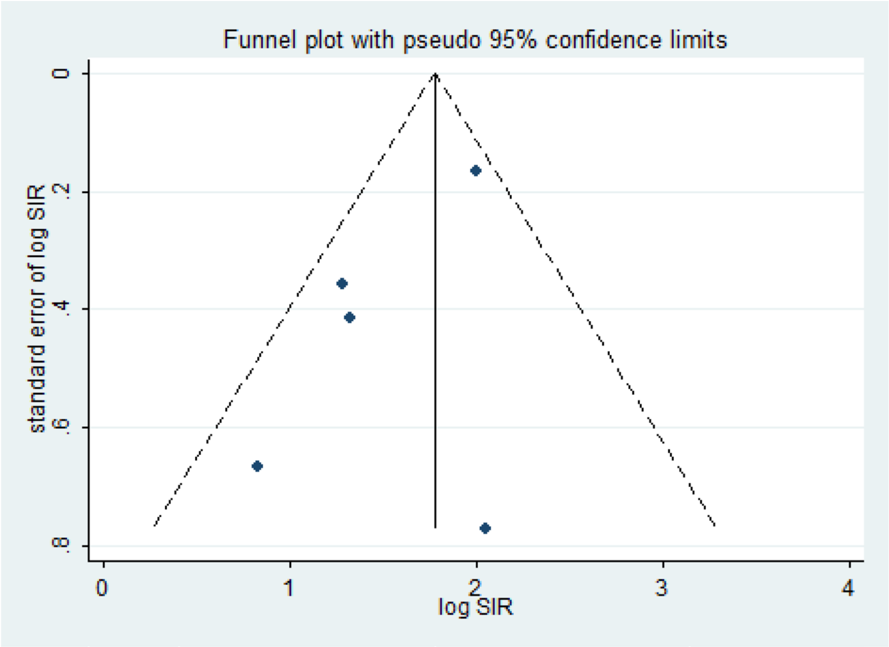

Supplementary Figure 12: Funnel plot for Hodgkin's lymphoma (*P* value for Egger: 0.184; *P* value for Begg: 1.000).

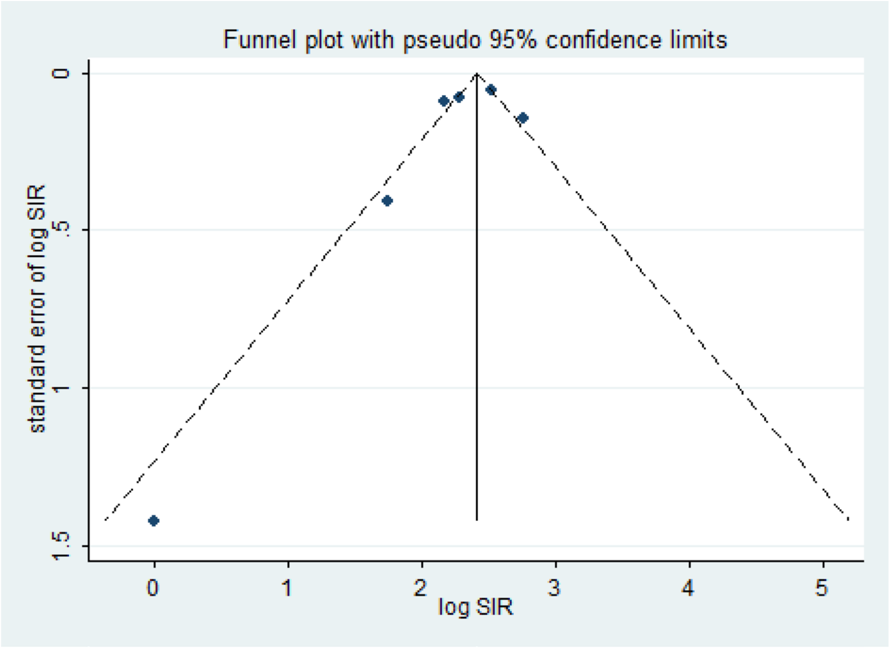

Supplementary Figure 13: Funnel plot for non-Hodgkin's lymphoma (*P* value for Egger: 0.366; *P* value for Begg: 0.707).

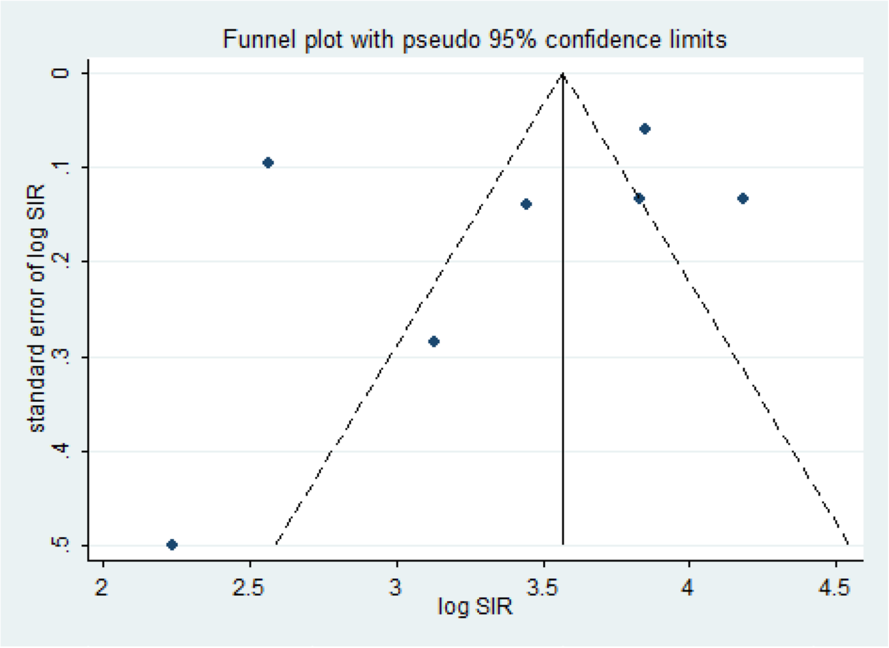

Supplementary Figure 14: Funnel plot for lip cancer (*P* value for Egger: 0.583; *P* value for Begg: 0.230).

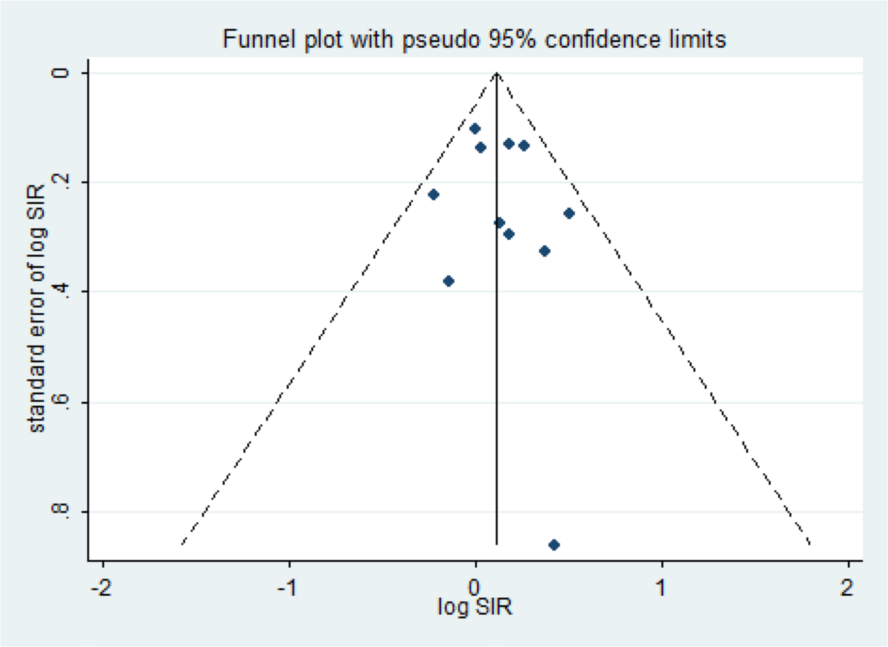

Supplementary Figure 15: Funnel plot for breast cancer (*P* value for Egger: 0.523; *P* value for Begg: 0.755).

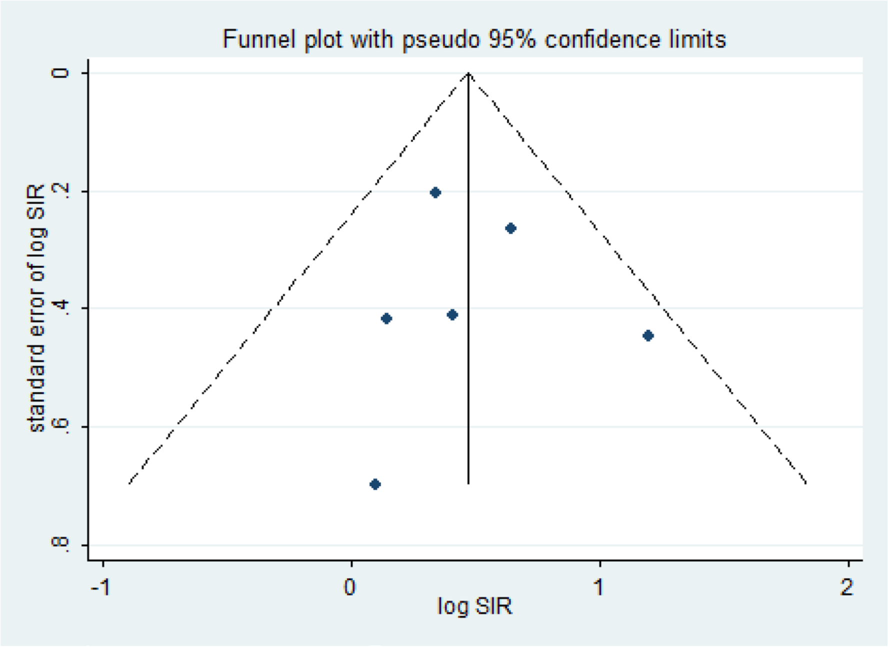

Supplementary Figure 16: Funnel plot for ovarian cancer (*P* value for Egger: 0.859; *P* value for Begg: 0.707).

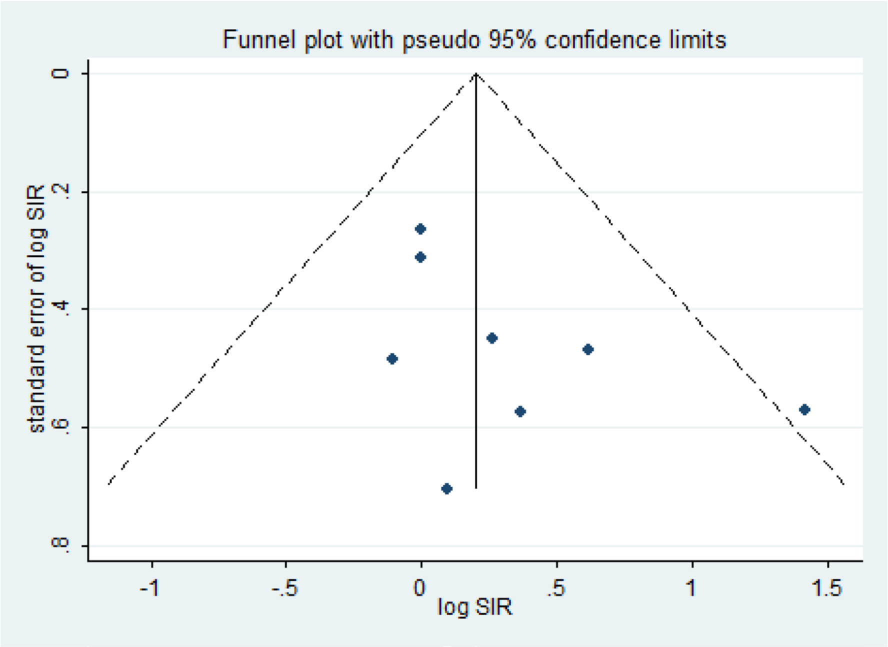

Supplementary Figure 17: Funnel plot for uterus cancer (*P* value for Egger: 0.158; *P* value for Begg: 0.174).

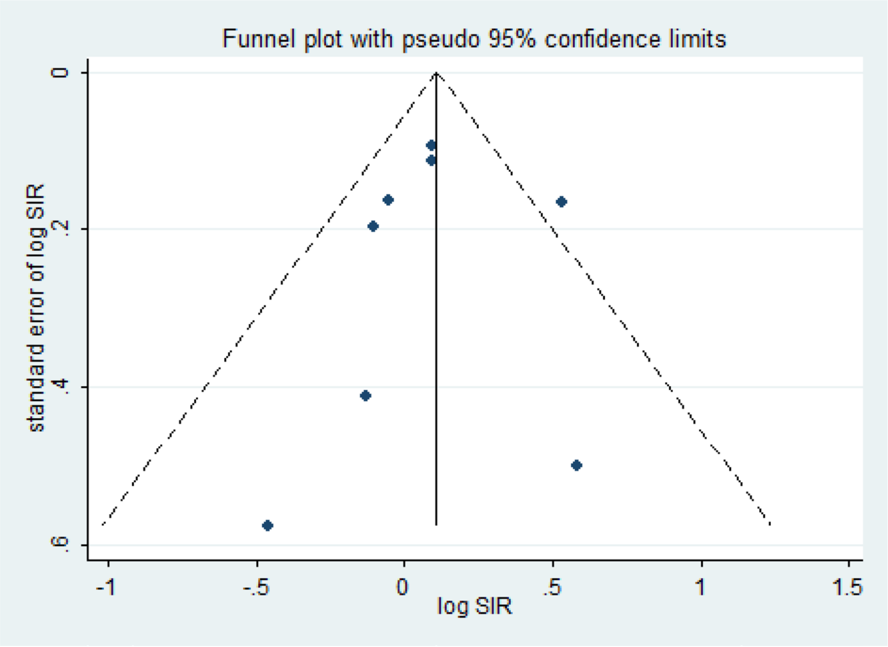

Supplementary Figure 18: Funnel plot for prostate cancer (*P* value for Egger: 0.835; *P* value for Begg: 0.902).
